# Supplementary material for: Design, Synthesis and Biological Evaluation of Novel 1,3,5-Triazines: Effect of Aromatic Ring Decoration on Affinity to 5-HT7 Receptor
Source: Int J Mol Sci. 2022 Nov 1;23(21):13308. doi: 10.3390/ijms232113308 (PMC9656787; doi:10.3390/ijms232113308)

Design, Synthesis and Biological Evaluation of Novel 1,3,5-Triazines: Effect of Aromatic Ring Decoration on Affinity to 5-HT7 Receptor

Damian Kułaga 1,* , Anna Karolina Drabczyk 1 , Grzegorz Satała 2, Gniewomir Latacz 3 ,
Anna Boguszewska-Czubara 4 , Damian Pla˙zuk 5 and Jolanta Ja´skowska 1

1 Department of Organic Chemistry and Technology, Faculty of Chemical Engineering and Technology, Cracow University of Technology, ul. Warszawska 24, 31-155 Kraków, Poland
2 Department ofMedicinal Chemistry,Maj Institute of Pharmacology, Polish Academy of Sciences, ul. Sm˛etna 12, 31-343 Kraków, Poland
3 Department of Technology and Biotechnology of Drugs, Jagiellonian University Medical College, ul. Medyczna 9, 30-688 Kraków, Poland
4 Department of Medical Chemistry, Medical University of Lublin, ul. Chod´zki 4a, 20-093 Lublin, Poland
5 Laboratory of Molecular Spectroscopy, Department of Organic Chemistry, Faculty of Chemistry, University of Lodz, ul. Tamka 12, 91-403 Łód´z, Poland
* Correspondence: damian.kulaga@pk.edu.pl

Supplementary Material

1. Characterization of the intermediates……………………………………………………………………2-4
2. Proposed elimination of ethyl linker for ligands 20-22 – HPLC-MS spectra…………………………5-10
3. Reaction of 69 with p-fluoroaniline……………………………………………………………………..11-12
2. Characterization of final products (^{1}H NMR, ^{13}C NMR, HPLC-MS)……………………………………13-54
3. Metabolic stability UPLC-MS Spectra for ligands 2 and 12 …………………………………………..55-58
4. Ramachandran plot for 5-HT$_7$ homology model……………………………………………………………59
5. Validation of the5-HT$_7$ homology model………………………………………………………………………60

Characterization of the intermediates

HPLC-MS analysis of all intermediated was carried out according to B method

<u>compounds **29 – 34**</u>

*3-formyl-1H-indole-5-carbonitrile (**29**)*
pale yellow solid (97% yield); mp.: 247-248 °C; ESI+MS calc. for $C_{10}H_6N_2O$ m/z=170; found m/z=171 $[M+H]^+$

*5-fluoro-1H-indole-3-carbaldehyde (**30**)*
pale grey solid (92% yield); mp.: 159-162 °C

*5-bromo-1H-indole-3-carbaldehyde (**31**)*
pale yellow solid (99% yield); mp.: 197-199 °C; ESI+MS calc. for C_9H_6BrNO m/z=223; found m/z=222 $[M-H]^-$

*5-chloro-1H-indole-3-carbaldehyde (**32**)*
pale yellow solid (95% yield); mp.: 164-168 °C; ESI+MS calc. for C_9H_6ClNO m/z=179; found m/z=180 $[M+H]^+$

*5-methoxy-1H-indole-3-carbaldehyde (**33**)*
pale gray solid (100% yield); mp.: 172-174 °C; ESI+MS calc. for $C_{10}H_9NO_2$ m/z=175; found m/z=176 $[M+H]^+$

*5-methyl-1H-indole-3-carbaldehyde (**34**)*
pale gray solid (90% yield); mp.: 126-128 °C; ESI+MS calc. for $C_{10}H_9NO$ m/z=159; found m/z=160 $[M+H]^+$

<u>compounds **35 – 40**</u>

*3-(2-nitrovinyl)-1H-indole-5-carbonitrile (**35**)*
orange solid (90% yield); mp.: 236-240 °C; ESI+MS calc. for $C_{11}H_7N_3O_2$ m/z=213; found m/z=212 $[M-H]^+$

*5-fluoro-3-(2-nitrovinyl)-1H-indole (**36**)*
red solid (92% yield); mp.: 140-142 °C; ESI+MS calc. for $C_{10}H_7FN_2O_2$ m/z=206; found m/z=205 $[M-H]^-$

*5-bromo-3-(2-nitrovinyl)-1H-indole (**37**)*
red solid (91% yield); mp.: 193-196 °C; ESI+MS calc. for $C_{10}H_7BrN_2O_2$ m/z=267; found m/z=267

*5-chloro-3-(2-nitrovinyl)-1H-indole (**38**)*
dark red solid (93% yield); mp.: 169-172 °C; ESI+MS calc. for $C_{10}H_7ClN_2O_2$ m/z=222; found m/z=221 $[M-H]^-$

*5-methoxy-3-(2-nitrovinyl)-1H-indole (**39**)*
red solid (95% yield); mp.: 150-154 °C

*5-methyl-3-(2-nitrovinyl)-1H-indole (**40**)*
red solid (96% yield); mp.: 120-124°C; ESI+MS calc. for $C_{11}H_{10}N_2O_2$ m/z=202; found m/z=201 $[M-H]^-$

compounds **42 – 46**

*2-(5-fluoro-1H-indol-3-yl)ethanamine (**42**)*
brown oil (52% yield); ESI+MS calc. for $C_{10}H_{11}FN_2$ m/z=178; found m/z=179 $[M+H]^+$

*2-(5-bromo-1H-indol-3-yl)ethanamine (**43**)*
brown oil (49% yield); ESI+MS calc. for $C_{10}H_{11}BrN_2$ m/z=239; found m/z=240 $[M+H]^+$

*2-(5-chloro-1H-indol-3-yl)ethanamine (**44**)*
dark brown oil (55% yield); ESI+MS calc. for $C_{10}H_{11}ClN_2$ m/z=194; found m/z=195 $[M+H]^+$

*2-(5-methoxy-1H-indol-3-yl)ethanamine (**45**)*
brown oil (68% yield); ESI+MS calc. for $C_{11}H_{14}N_2O$ m/z=190; found m/z=191 $[M+H]^+$

*2-(5-methyl-1H-indol-3-yl)ethanamine (**46**)*
brown oil (43% yield); ESI+MS calc. for $C_{11}H_{14}N_2$ m/z=174; found m/z=175 $[M+H]^+$

compounds **61 – 68**

*N^1-(2-chlorophenyl)ethane-1,2-diamine hydrochloride (**61**)*
gray solid (96% yield); mp.: 200-201 °C; ESI+MS calc. for $C_8H_{11}ClN_2$ m/z=170; found m/z=171 $[M+H]^+$

*N^1-(3-chlorophenyl)ethane-1,2-diamine hydrochloride (**62**)*
gray solid (83% yield); mp.: 198-199 °C; ESI+MS calc. for $C_8H_{11}ClN_2$ m/z=170; found m/z=171 $[M+H]^+$

*N^1-(4-chlorophenyl)ethane-1,2-diamine hydrochloride (**63**)*
gray solid (90% yield); mp.: 216-218 °C; ESI+MS calc. for $C_8H_{11}ClN_2$ m/z=170; found m/z=171 $[M+H]^+$

*N^1-(2-fluorophenyl)ethane-1,2-diamine hydrochloride (**64**)*
gray solid (77% yield); mp.: 240-244 °C; ESI+MS calc. for $C_8H_{11}FN_2$ m/z=154; found m/z=155 $[M+H]^+$

*N^1-(3-fluorophenyl)ethane-1,2-diamine hydrochloride (**65**)*
beige solid (83% yield); mp.: 233-235 °C; ESI+MS calc. for $C_8H_{11}FN_2$ m/z=154; found m/z=155 $[M+H]^+$

*N^1-(4-fluorophenyl)ethane-1,2-diamine hydrochloride (**66**)*
beige solid (93% yield); mp.: 231-234 °C; ESI+MS calc. for $C_8H_{11}FN_2$ m/z=154; found m/z=155 $[M+H]^+$

*N^1-(3-methoxyphenyl)ethane-1,2-diamine hydrochloride (**67**)*
beige solid (87% yield); mp.: 187-191 °C; ESI+MS calc. for $C_9H_{14}N_2O$ m/z=166; found m/z=167 $[M+H]^+$

*N^1-(4-methoxyphenyl)ethane-1,2-diamine hydrochloride (**68**)*
purple solid (70% yield); mp.: 190-192 °C; ESI+MS calc. for $C_9H_{14}N_2O$ m/z=166; found m/z=167 $[M+H]^+$

compounds **75 – 78**

*2-(2-aminoethyl)isoindoline-1,3-dione hydrochloride (**75**)*
white solid (60% yield); mp.: 201-203 oC; ESI+MS calc. for $C_{10}H_{10}N_2O_2$ m/z=190; found m/z=191 $[M+H]^+$

*2-(1H-benzo[d]imidazol-1-yl)ethanamine hydrochloride (**76**)*
yellow solid (66% yield); mp.: 139-142 ^{o}C

*2-(2-methyl-1H-benzo[d]imidazol-1-yl)ethanamine hydrochloride (**77**)*
white solid (72% yield); mp.: 251-254 ^{o}C

*2-(2-(trifluoromethyl)-1H-benzo[d]imidazol-1-yl)ethanamine hydrochloride (**78**)*
white solid (78% yield); mp.: 221-224 ^{o}C

Scheme 4, compound **20** (HPLC-MS analysis according to B method)

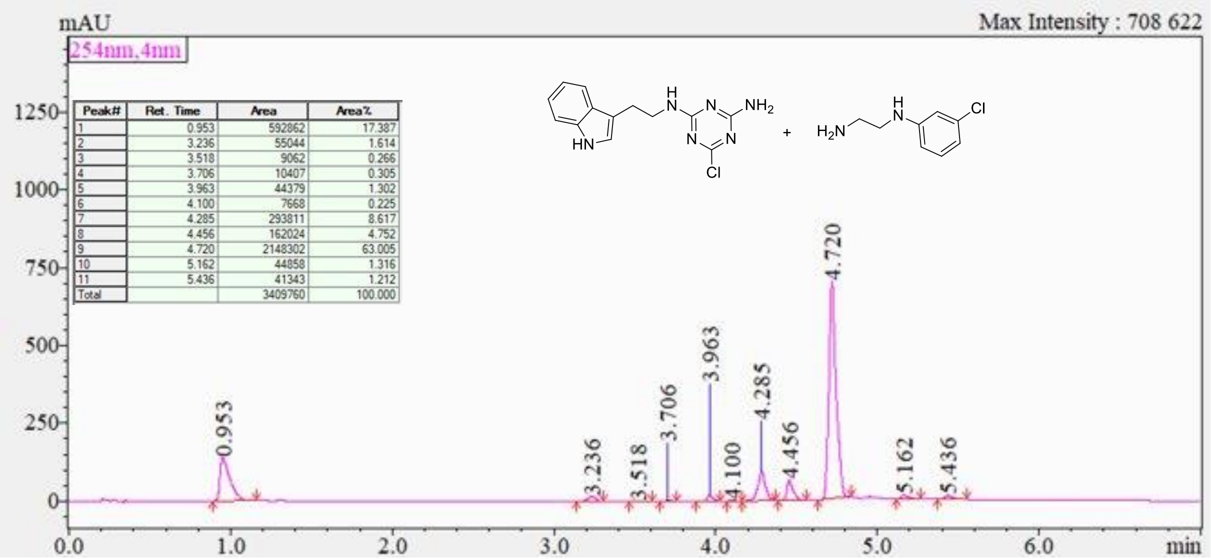

Peak#	Ret. Time	Area	Area%
1	0.953	592862	17.387
2	3.236	55044	1.614
3	3.518	9062	0.266
4	3.706	10407	0.305
5	3.963	44379	1.302
6	4.100	7668	0.225
7	4.285	293811	8.617
8	4.456	162024	4.752
9	4.720	2148302	63.005
10	5.162	44858	1.316
11	5.436	41343	1.212
Total		3409760	100.000

Peak 1 – t = 0.953 min

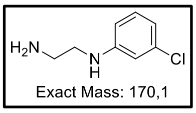

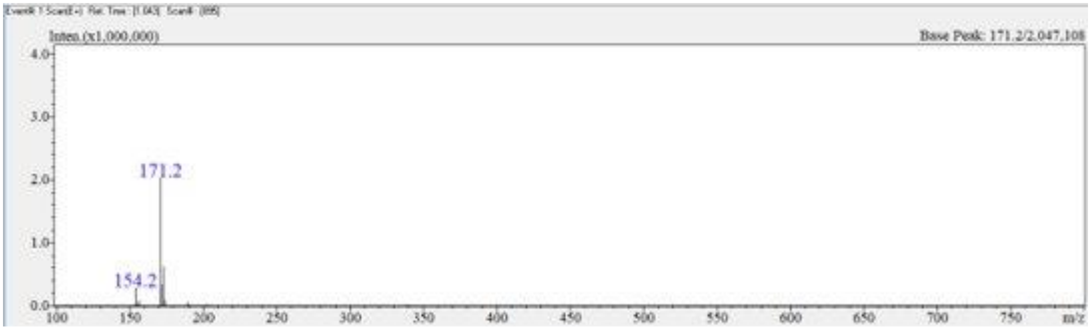

peak 7 – t = 4.285 min

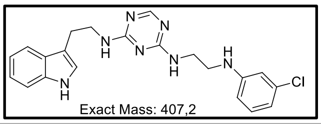

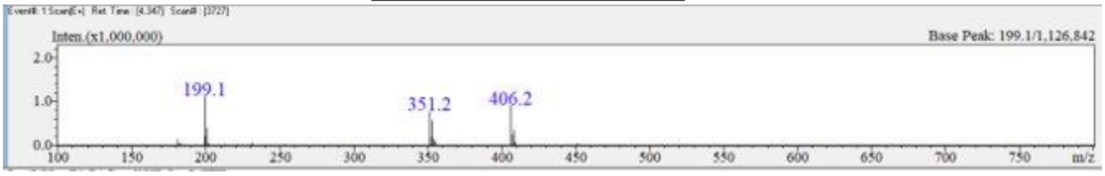

peak 8 – t = 4.456 min

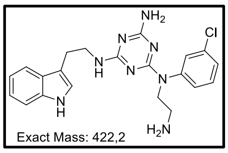

or

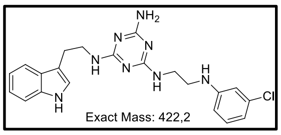

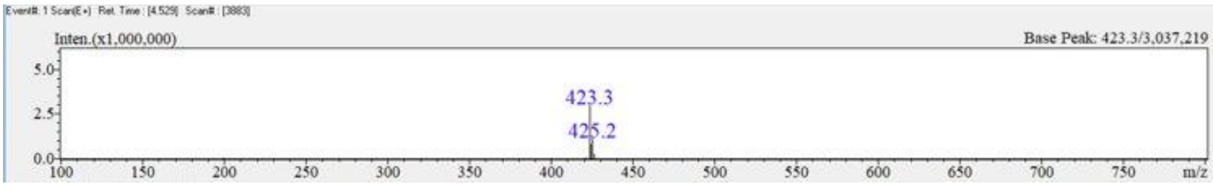

peak 9 – t = 4.720 min

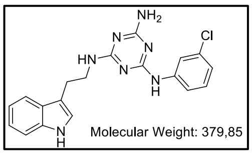

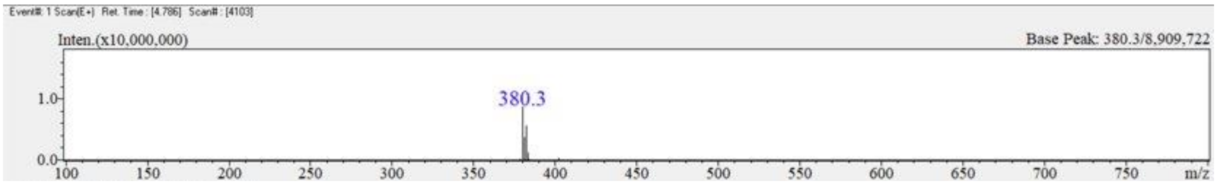

Scheme 4, compound **21** (HPLC-MS analysis according to B method)

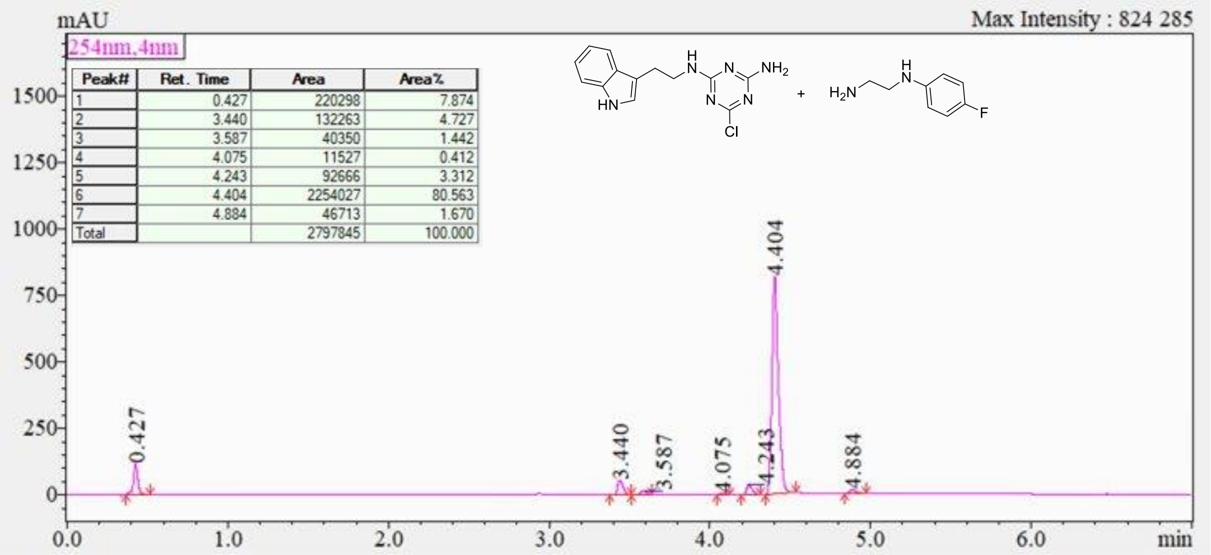

Peak#	Ret. Time	Area	Area%
1	0.427	220298	7.874
2	3.440	132263	4.727
3	3.587	40350	1.442
4	4.075	11527	0.412
5	4.243	92666	3.312
6	4.404	2254027	80.563
7	4.884	46713	1.670
Total		2797845	100.000

peak 1 – t = 0.427 min

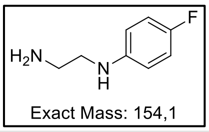

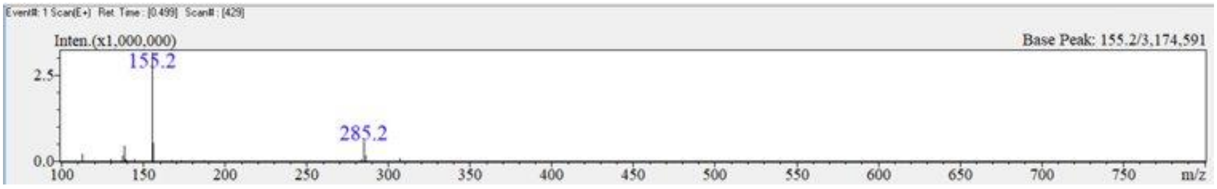

peak 2 – t = 3.440 min

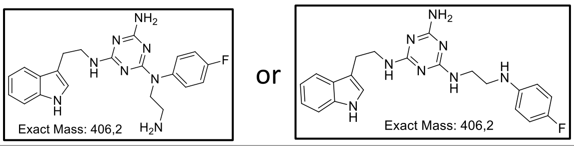

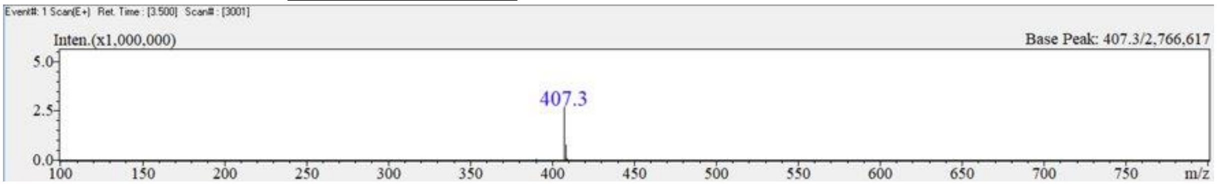

peak 5 – t = 4,243 min

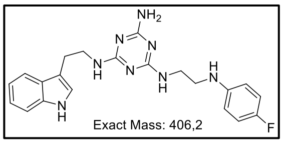

or

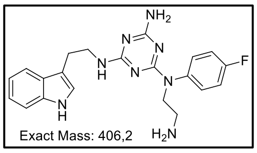

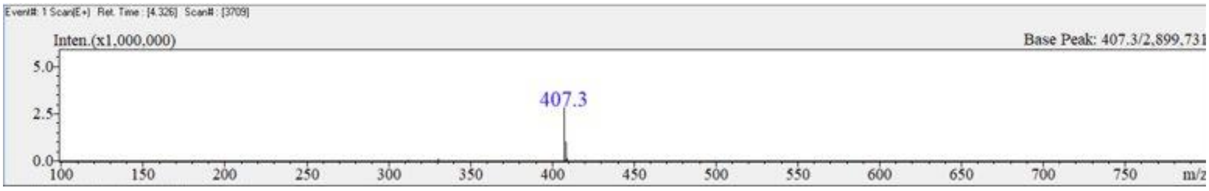

peak 6 – t = 4.404 min

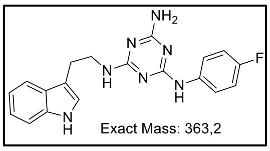

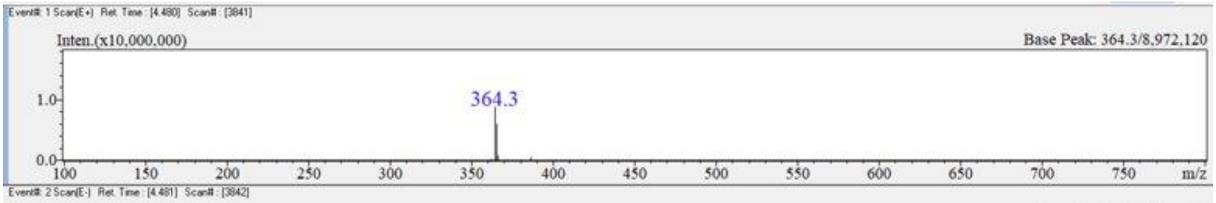

Scheme 4, compound **22** (HPLC-MS analysis according to B method)

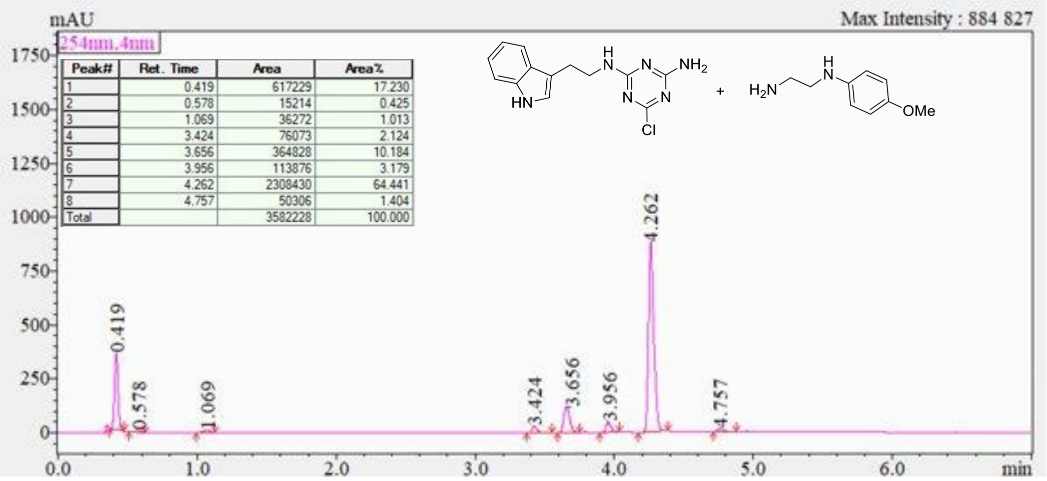

Peak#	Ret. Time	Area	Area%
1	0.419	617229	17.230
2	0.578	15214	0.425
3	1.069	36272	1.013
4	3.424	76073	2.124
5	3.656	364828	10.184
6	3.956	113876	3.179
7	4.262	2308430	64.441
8	4.757	50306	1.404
Total		3582228	100.000

peak 1 – t = 0.419 min

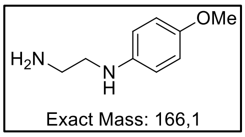

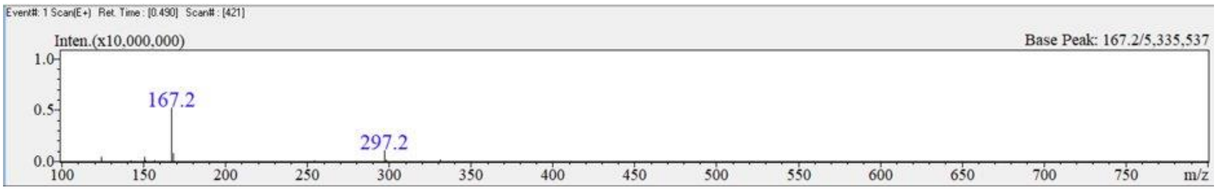

peak 4 – t = 3,424 min

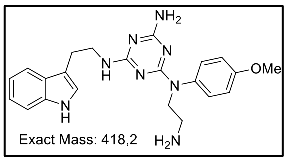

or

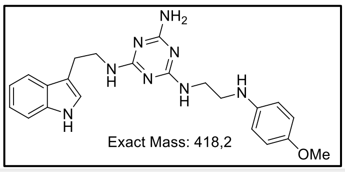

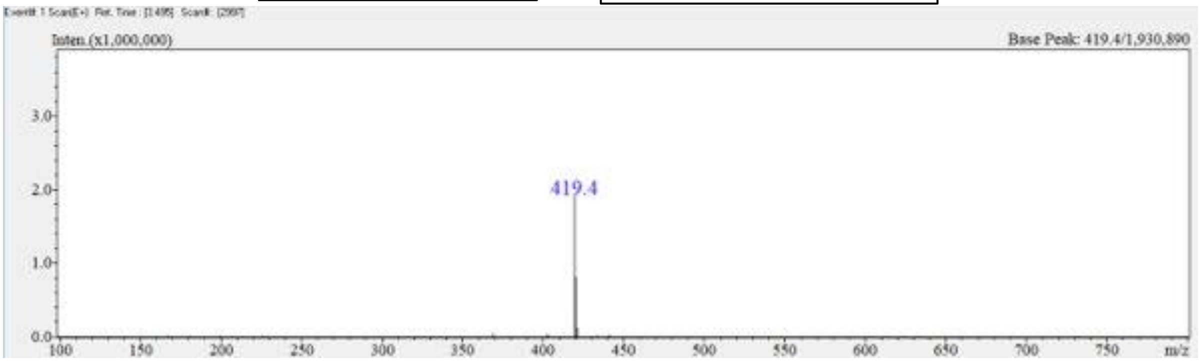

peak 6 – t = 3.956 min

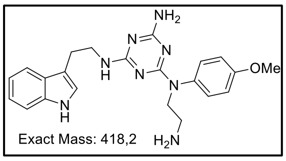

or

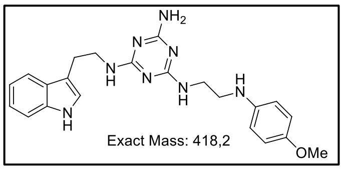

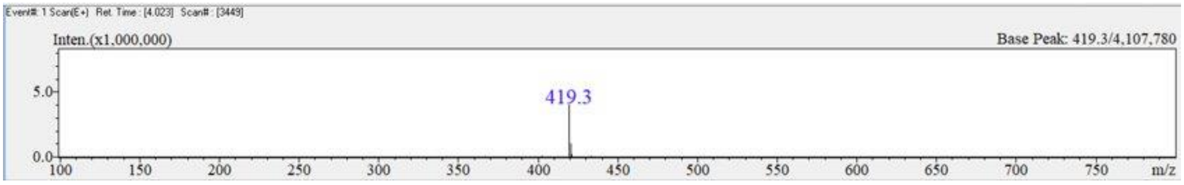

peak 7 – t = 4.262 min

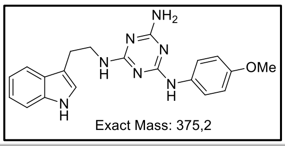

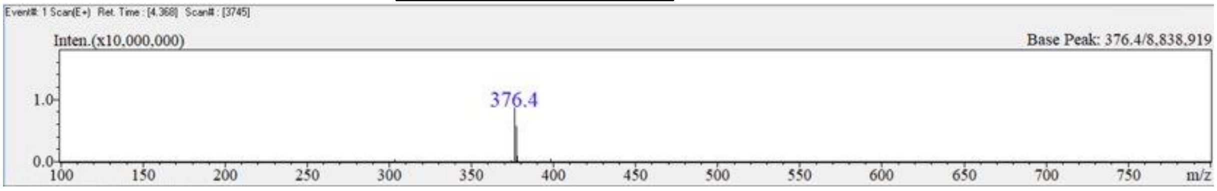

Scheme 5. Reaction of **69** with *p*-fluoroaniline in the presence of K_2CO_3 without purification (HPLC-MS analysis according to B method)

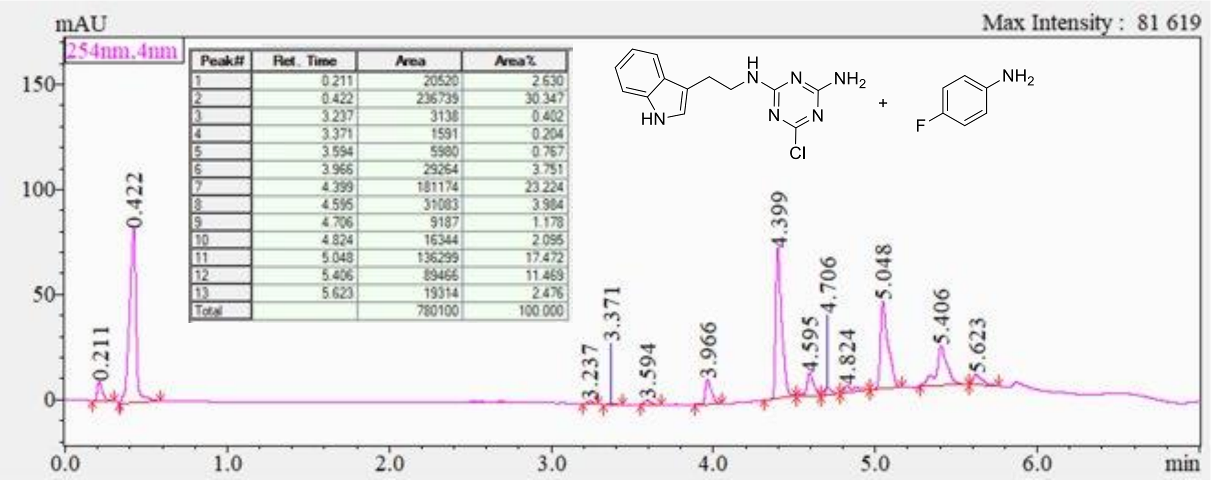

Peak#	Ret. Time	Area	Area%
1	0.211	20520	2.630
2	0.422	236739	30.347
3	3.237	3138	0.402
4	3.371	1591	0.204
5	3.594	5980	0.767
6	3.966	29264	3.751
7	4.399	181174	23.224
8	4.595	31083	3.984
9	4.706	9187	1.178
10	4.824	16344	2.095
11	5.048	136299	17.472
12	5.406	89466	11.469
13	5.623	19314	2.476
Total		780100	100.000

peak 7 – t = 4.399 min

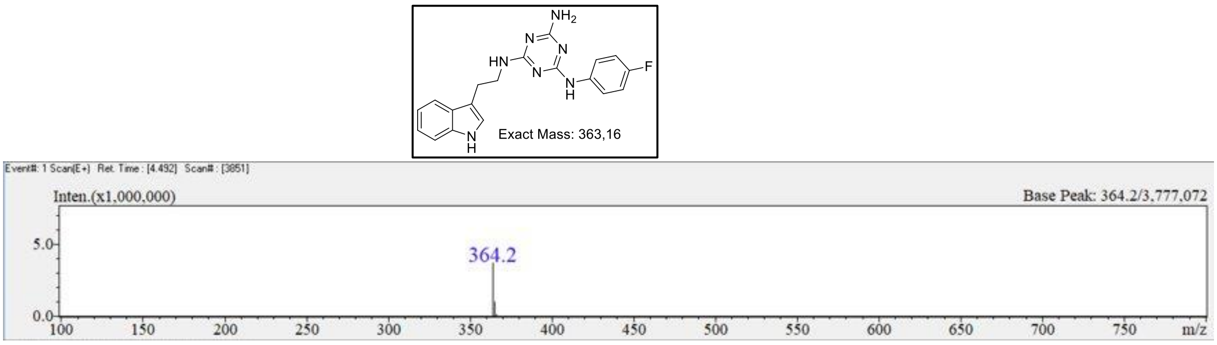

Scheme 5. Reaction of **69** with *p*-fluoroaniline in the presence of Na_2CO_3 without purification (HPLC-MS analysis according to B method)

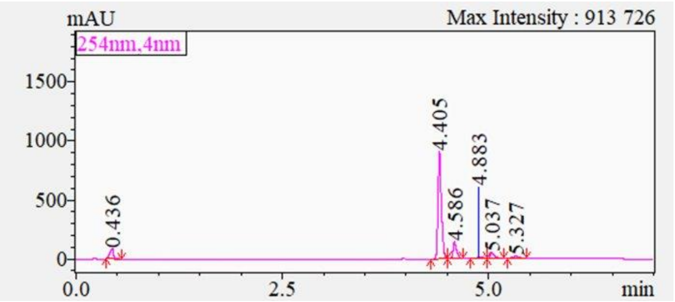

Peak#	Ret. Time	Area	Area%
1	0.436	260815	7.580
2	4.405	2540241	73.831
3	4.586	355717	10.339
4	4.883	62663	1.821
5	5.037	148992	4.330
6	5.327	72176	2.098
Total		3440603	100.000

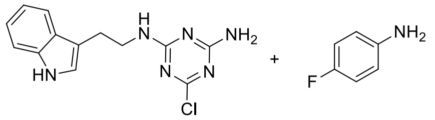

peak 2 – t = 4.405 min

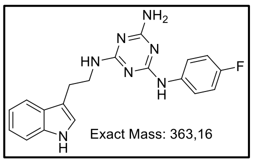

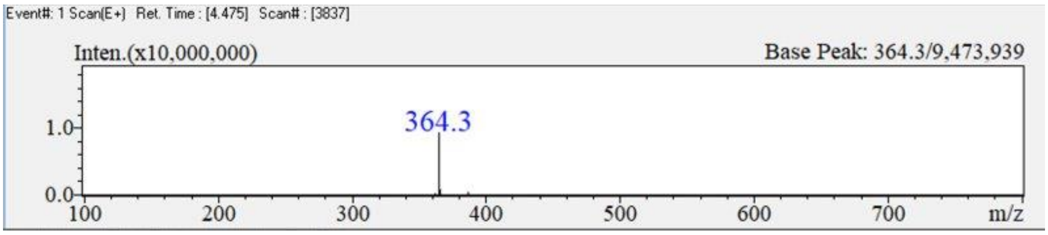

HPLC-MS of compound **1**

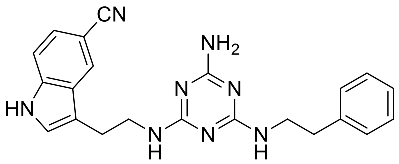

Chemical Formula: $C_{22}H_{22}N_8$
Exact Mass: 398,2

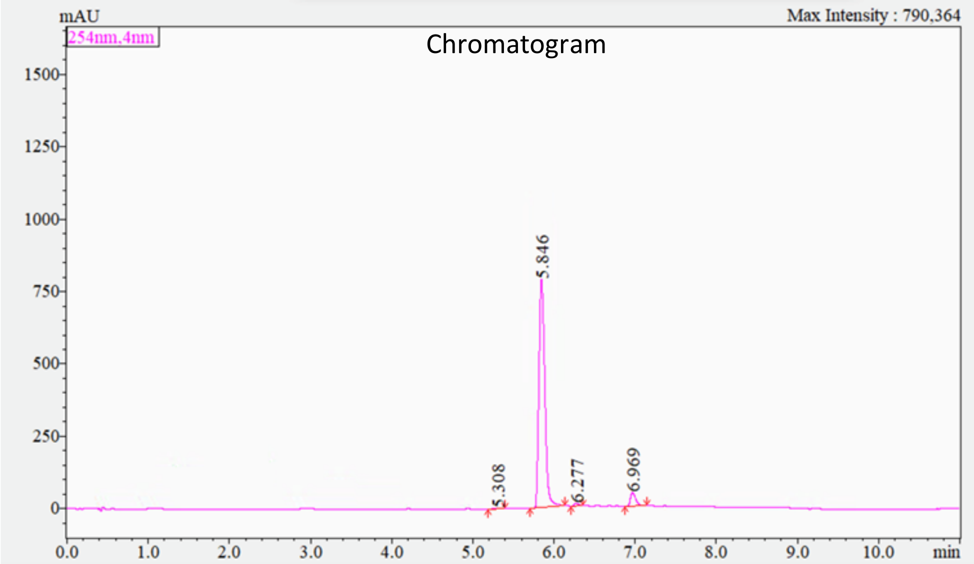

Peak#	Ret. Time	Area	Area%
1	5.308	12480	0.297
2	5.846	3950497	94.009
3	6.277	31003	0.738
4	6.969	208281	4.956
Total		4202261	100.000

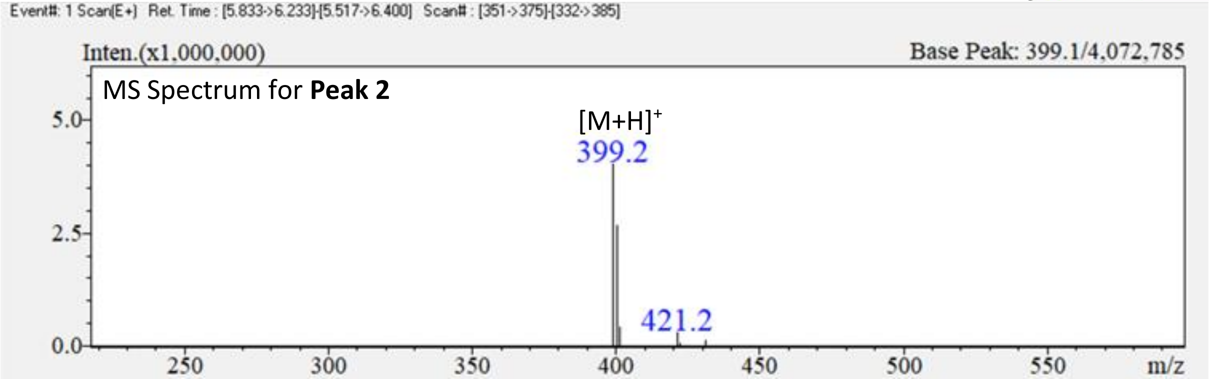

^{1}H NMR of compound **1** in CD_3OD

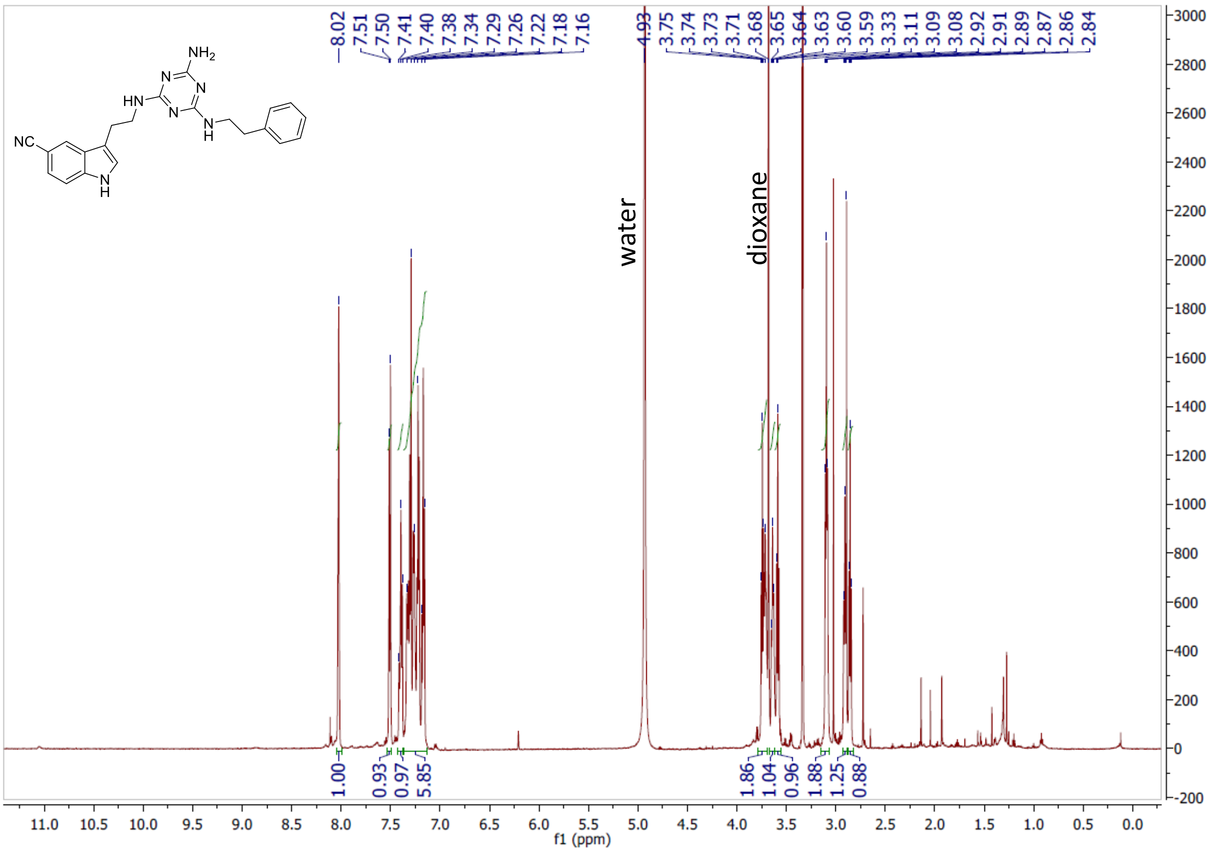

^{13}C NMR of compound **1** in CD_3OD

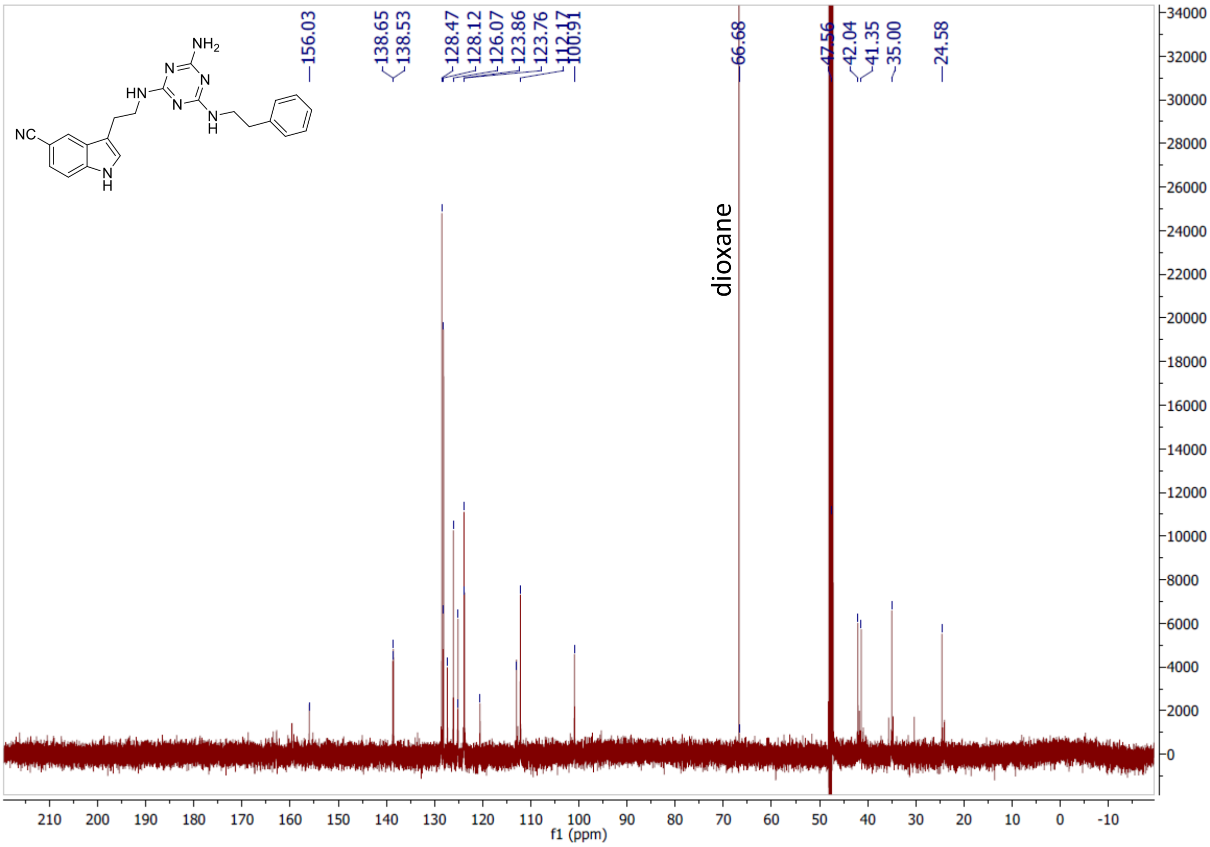

HPLC-MS of compound **2**

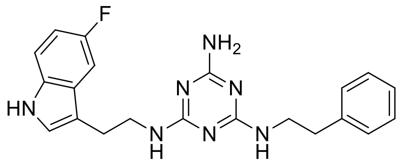

Chemical Formula: $C_{21}H_{22}FN_7$
Exact Mass: 391,2

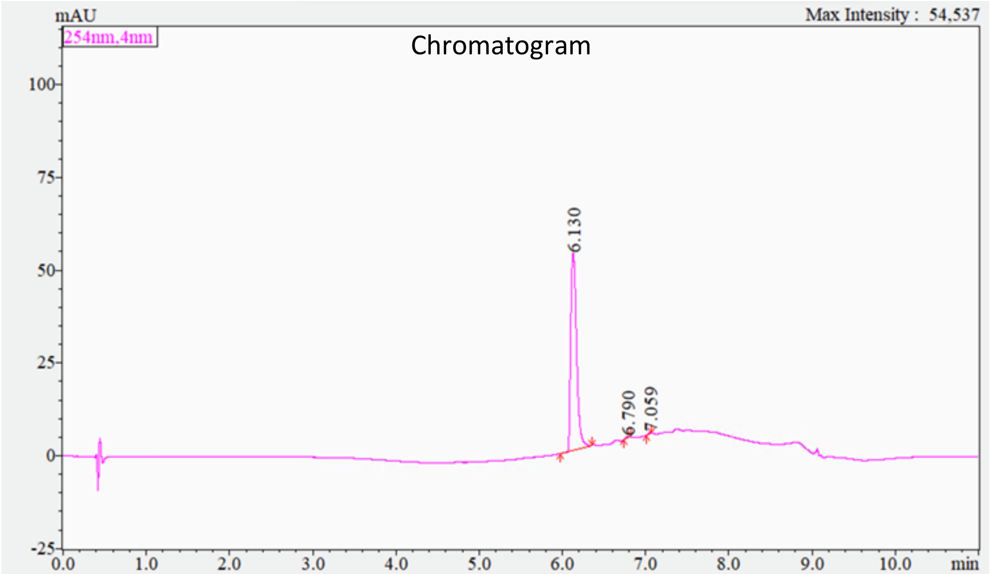

Peak#	Ret. Time	Area	Area%
1	6.130	258737	99.113
2	6.790	1591	0.609
3	7.059	725	0.278
Total		261054	100.000

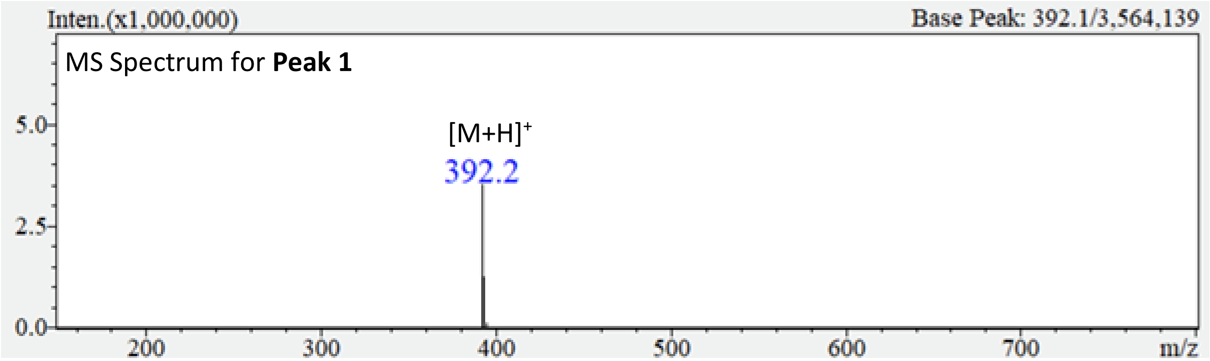

^{1}H NMR of compound **2** in CD_3OD

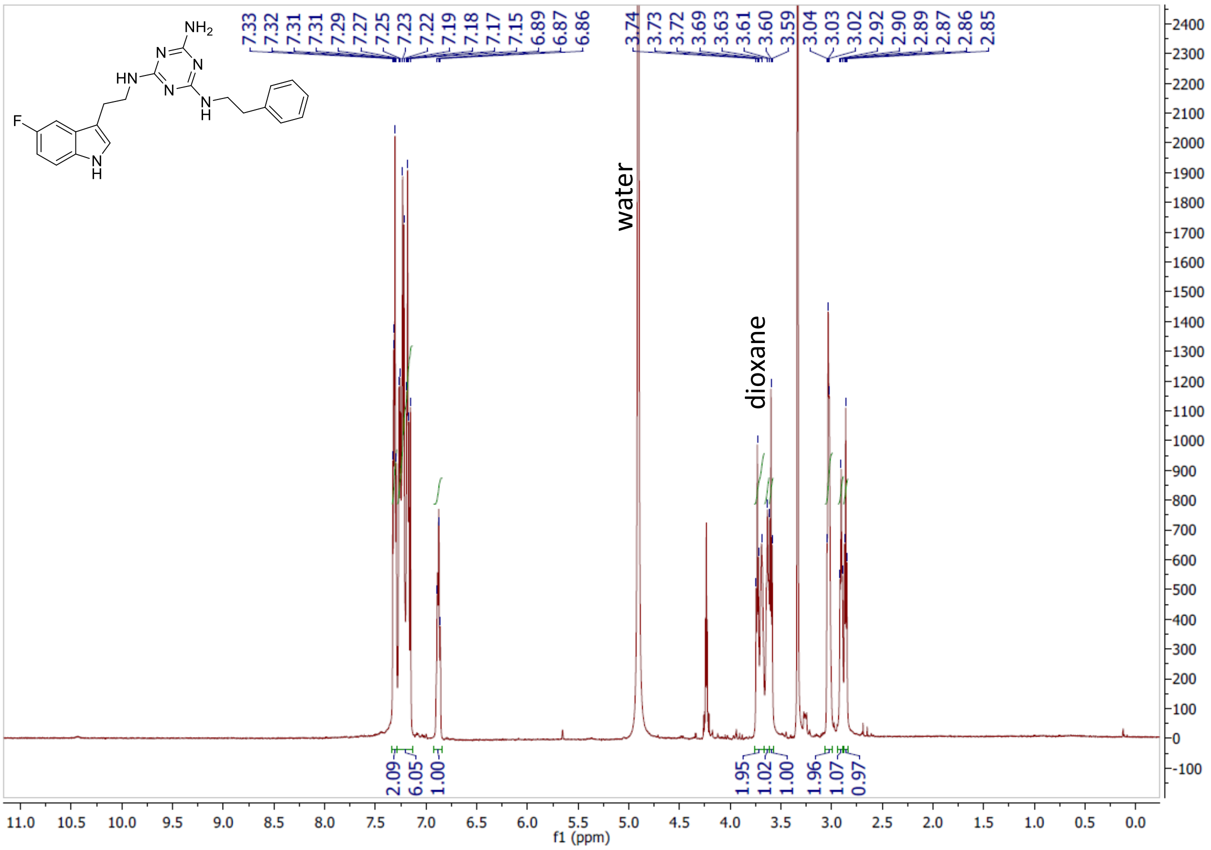

^{13}C NMR of compound **2** in CD_3OD

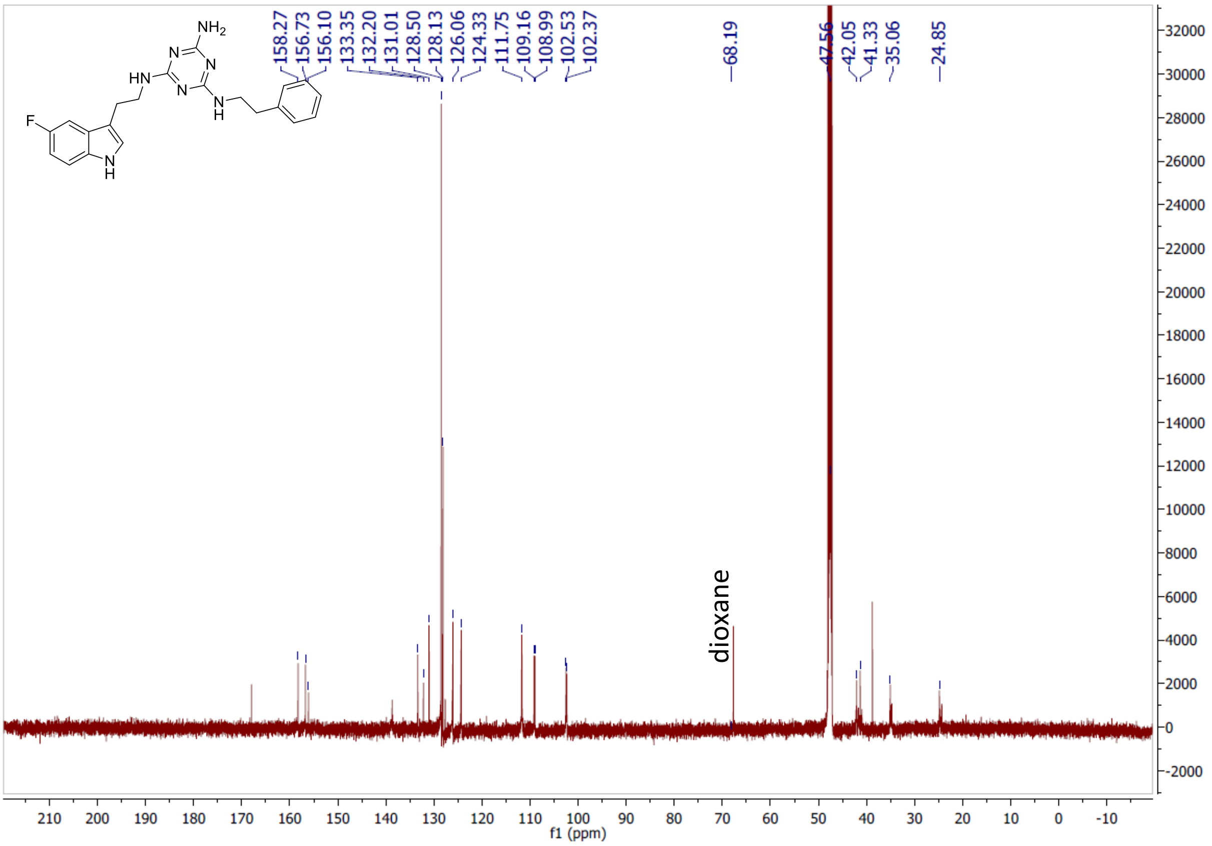

HPLC-MS of compound **3**

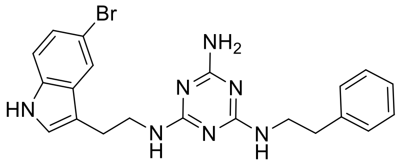

Chemical Formula: $C_{21}H_{22}{}^{79}BrN_7$
Exact Mass: 451,1

Chemical Formula: $C_{21}H_{22}{}^{81}BrN_7$
Exact Mass: 453,1

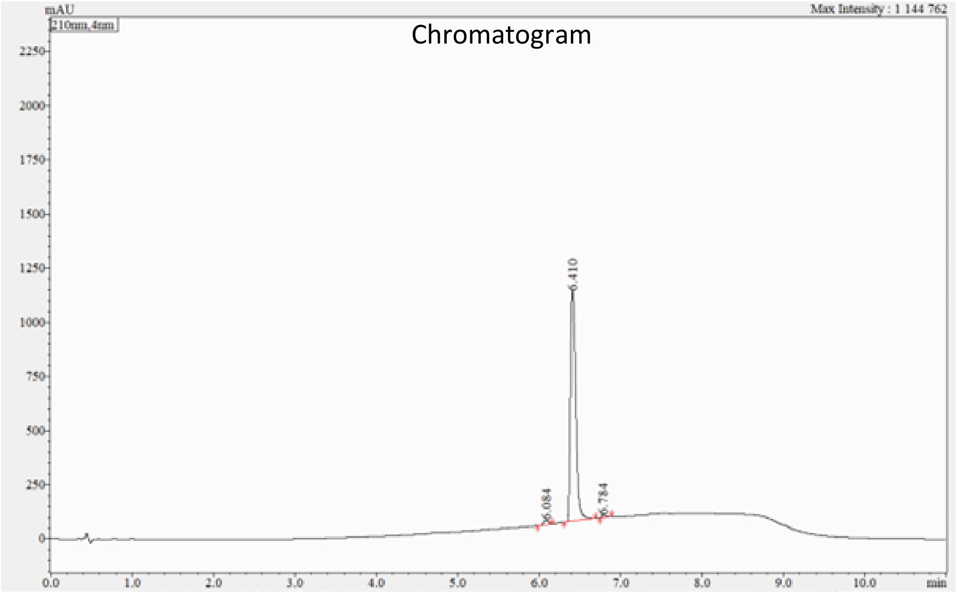

Peak#	Ret. Time	Area	Area%
1	6.084	80552	1.650
2	6.410	4761832	97.537
3	6.784	39697	0.813
Total		4882081	100.000

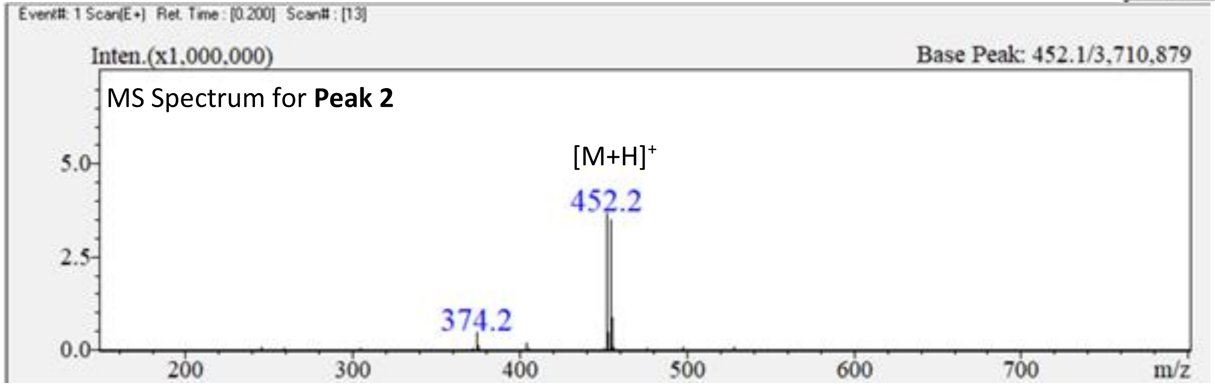

^{1}H NMR of compound **3** in CD_3OD

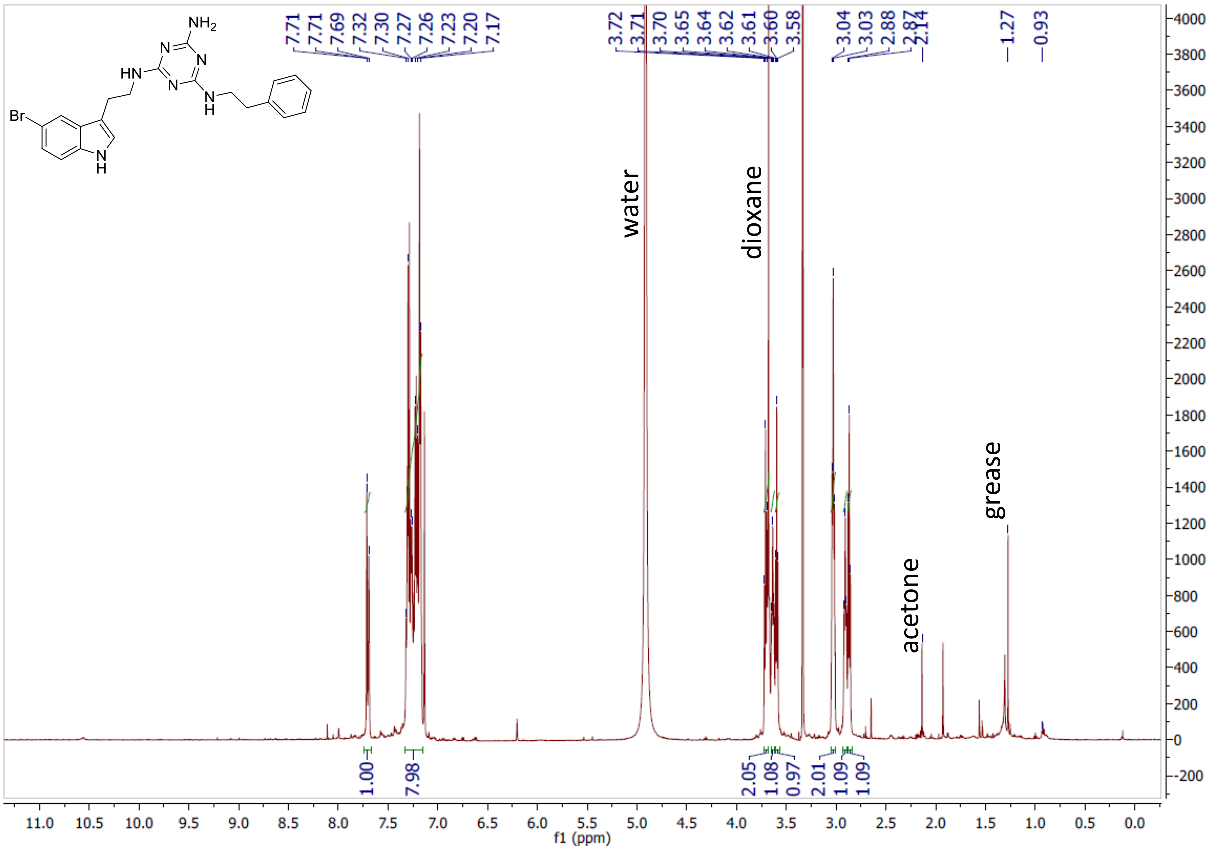

^{13}C NMR of compound **3** in CD_3OD

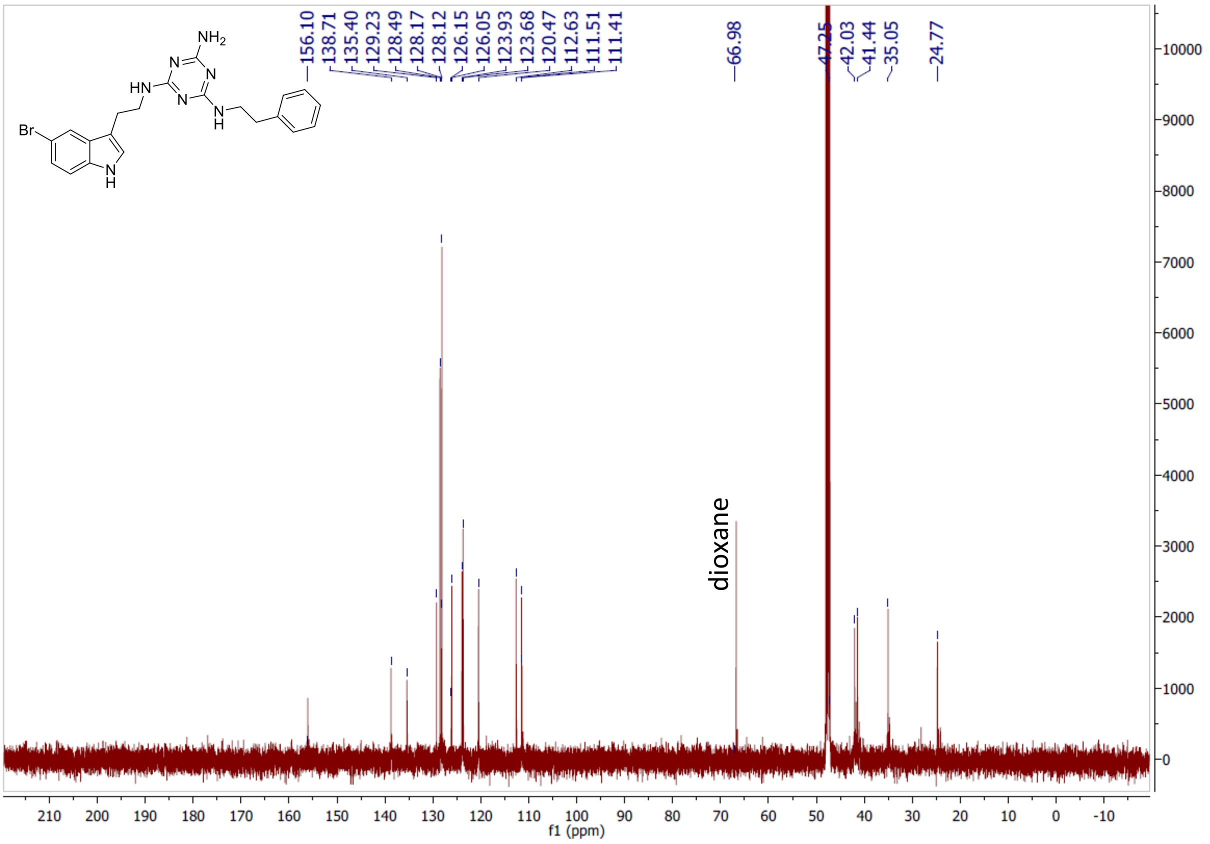

HPLC-MS of compound **4**

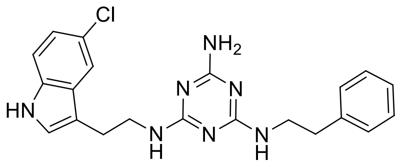

Chemical Formula: $C_{21}H_{22}{}^{35}ClN_7$
Exact Mass: 407,2
Chemical Formula: $C_{21}H_{22}{}^{37}ClN_7$
Exact Mass: 409,2

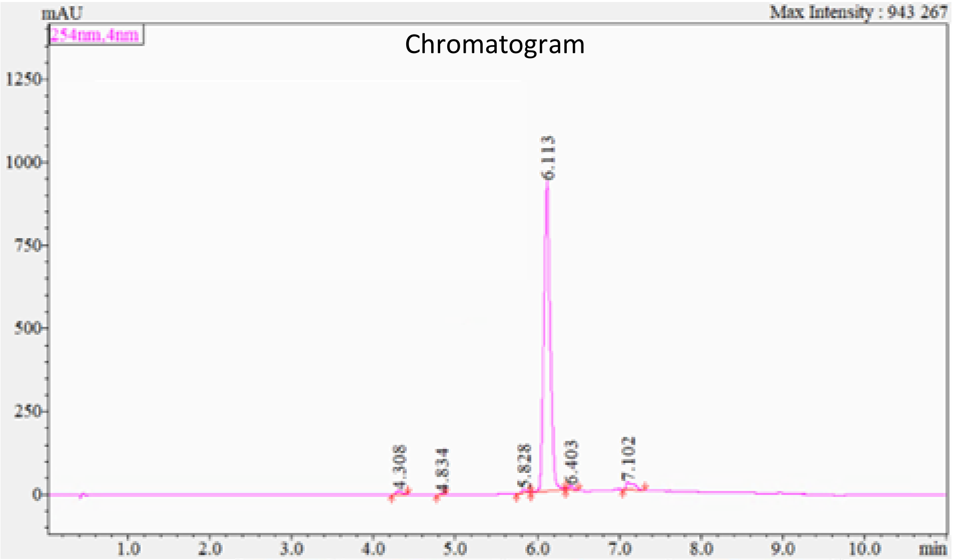

Peak#	Ret. Time	Area	Area%
1	4.308	48715	0.922
2	4.834	12203	0.231
3	5.828	50569	0.957
4	6.113	4964667	93.973
5	6.403	48474	0.918
6	7.102	158447	2.999
Total		5283075	100.000

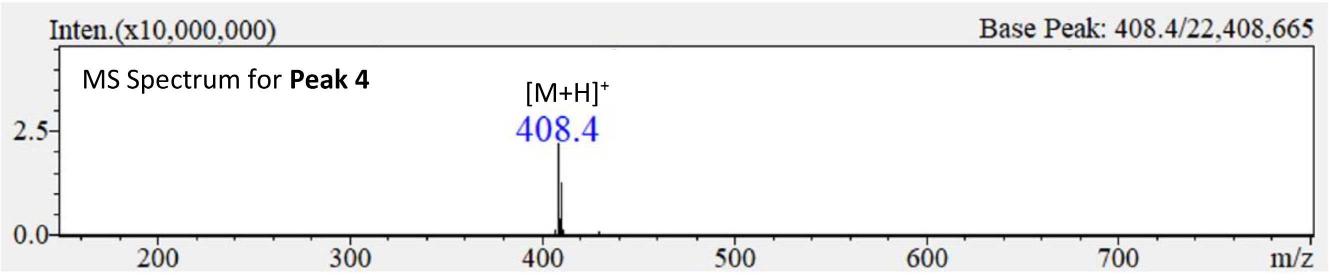

^{1}H NMR of compound **4** in CD_3OD

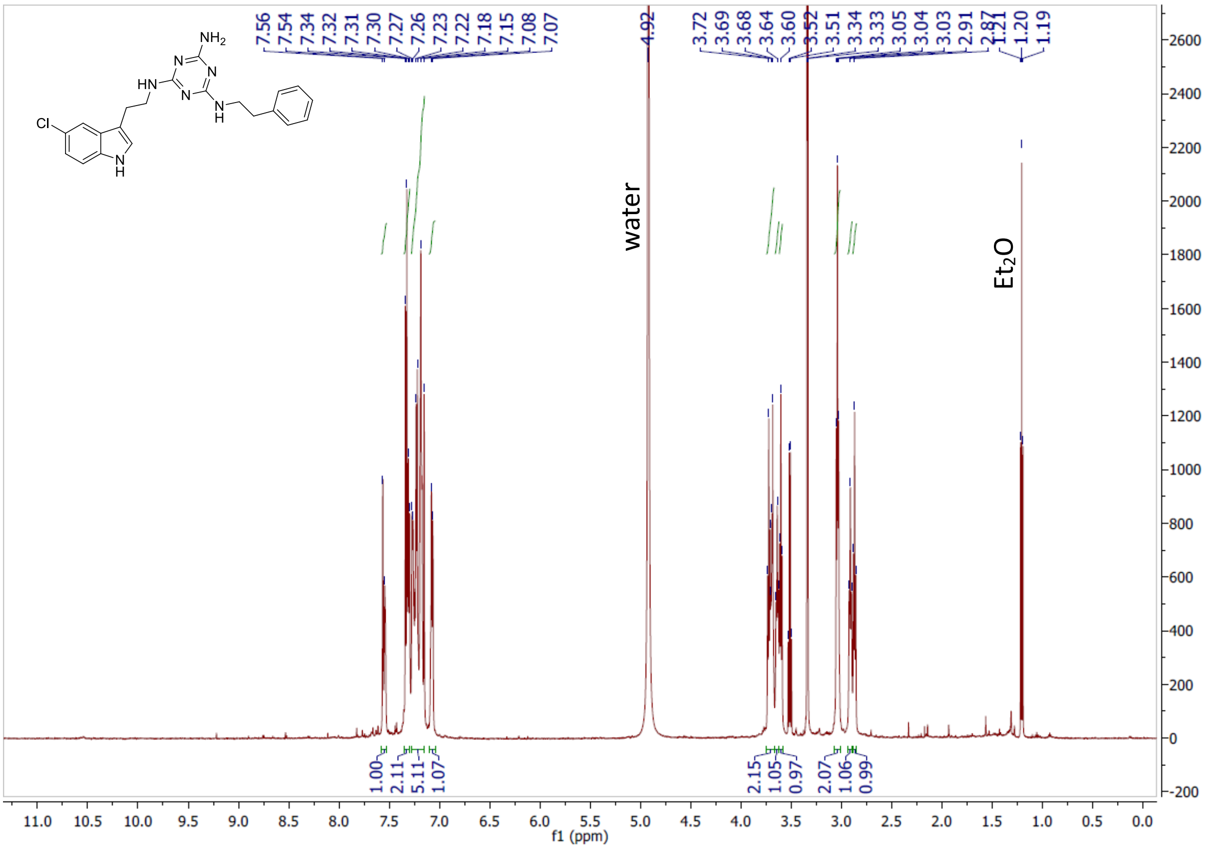

^{13}C NMR of compound **4** in CD_3OD

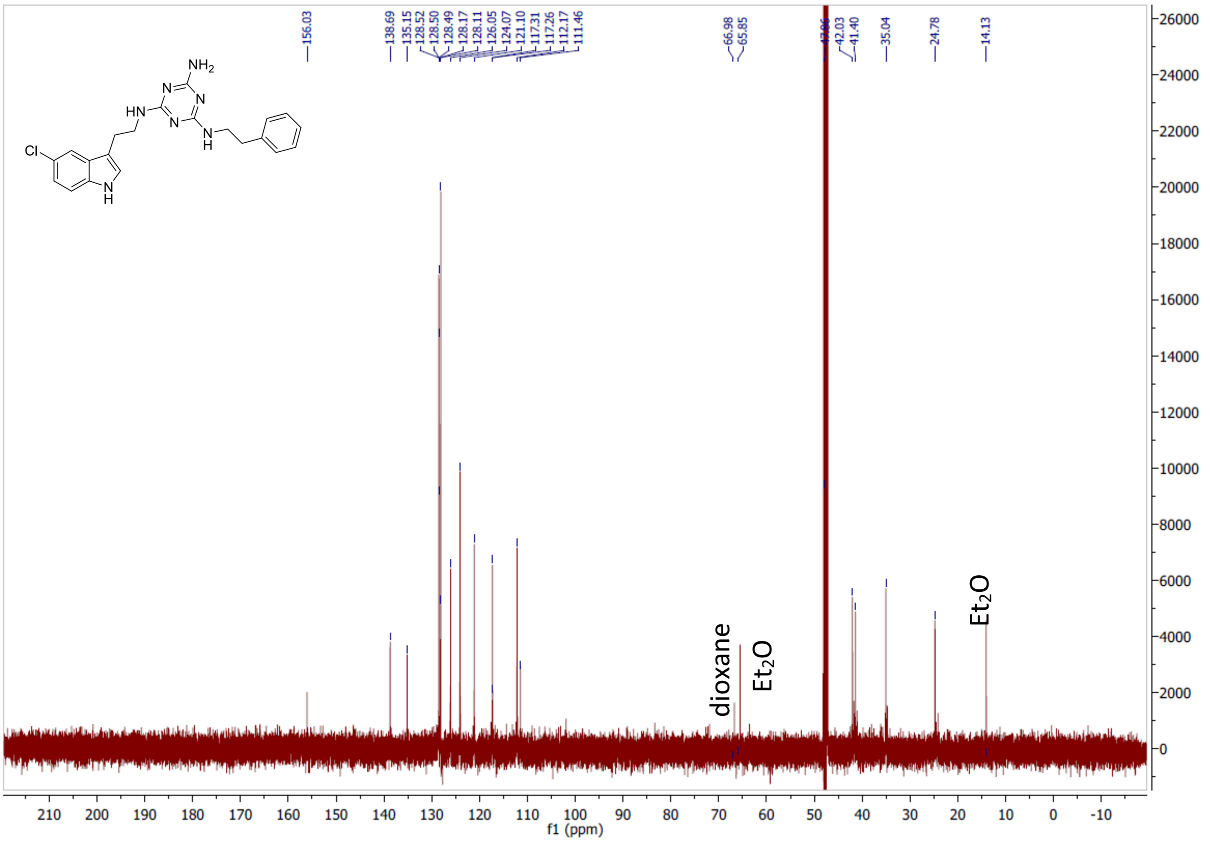

HPLC-MS of compound **5**

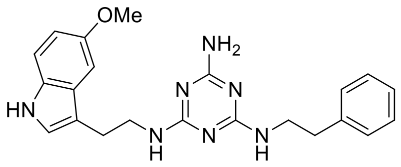

Chemical Formula: $C_{22}H_{25}N_7O$
Exact Mass: 403,2

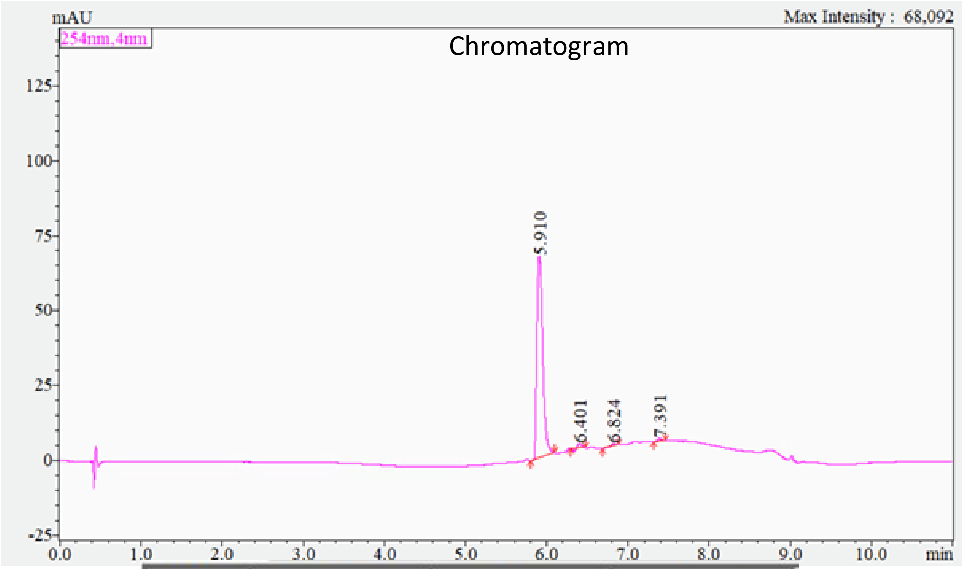

Peak#	Ret. Time	Area	Area%
1	5.910	334114	97.842
2	6.401	3122	0.914
3	6.824	1922	0.563
4	7.391	2324	0.681
Total		341482	100.000

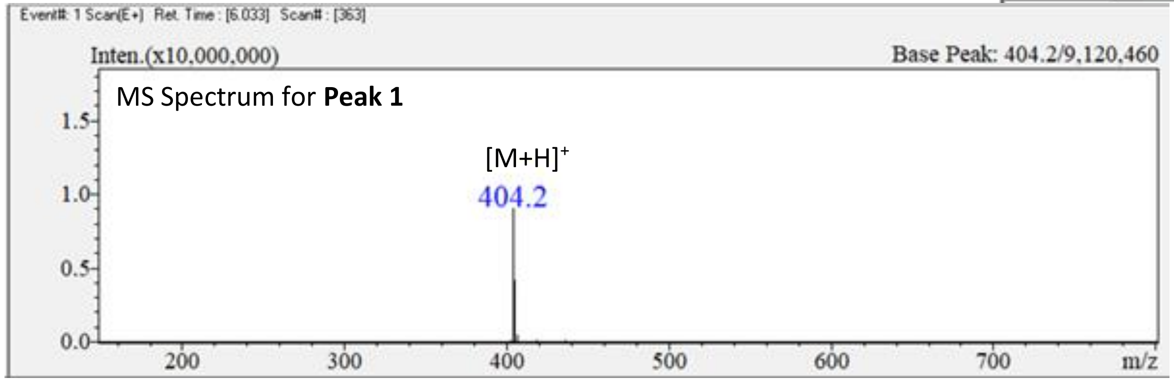

^{1}H NMR of compound **5** in CD_3OD

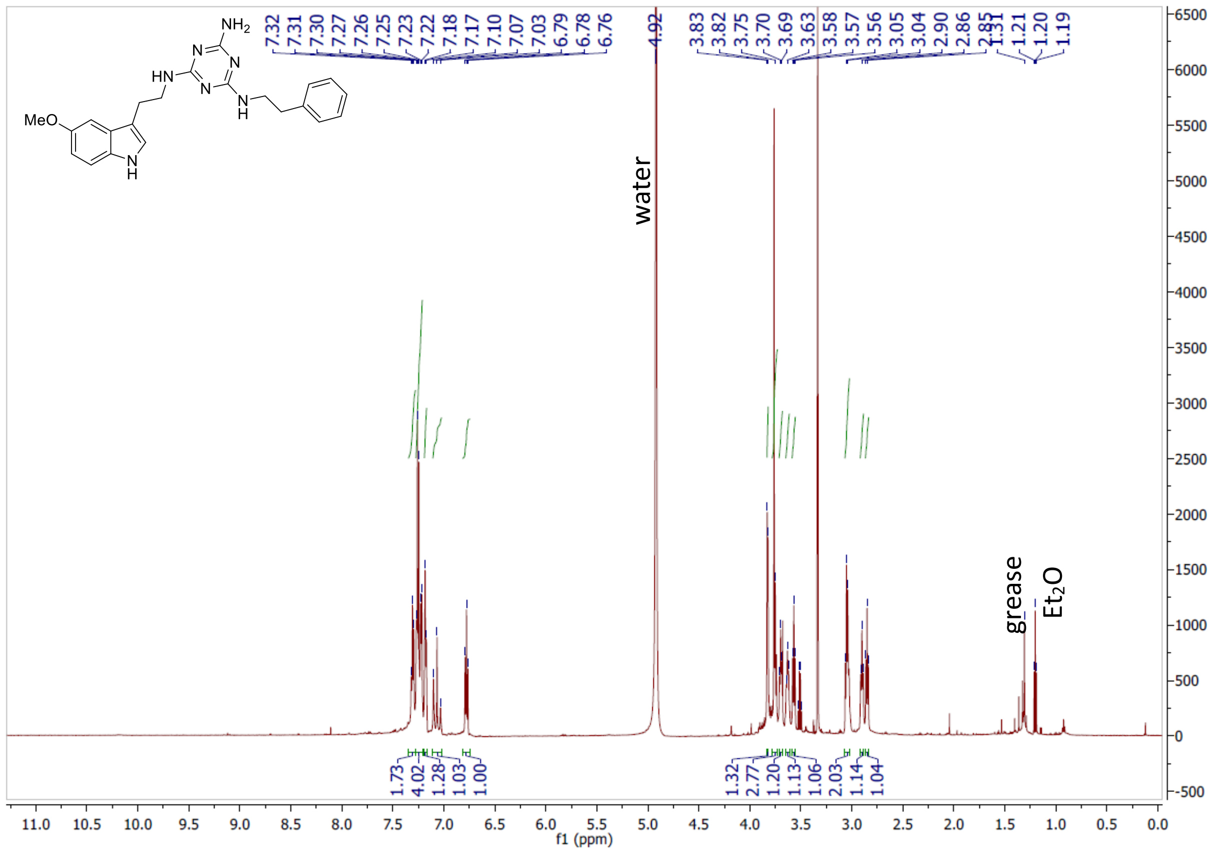

^{13}C NMR of compound **5** in CD_3OD

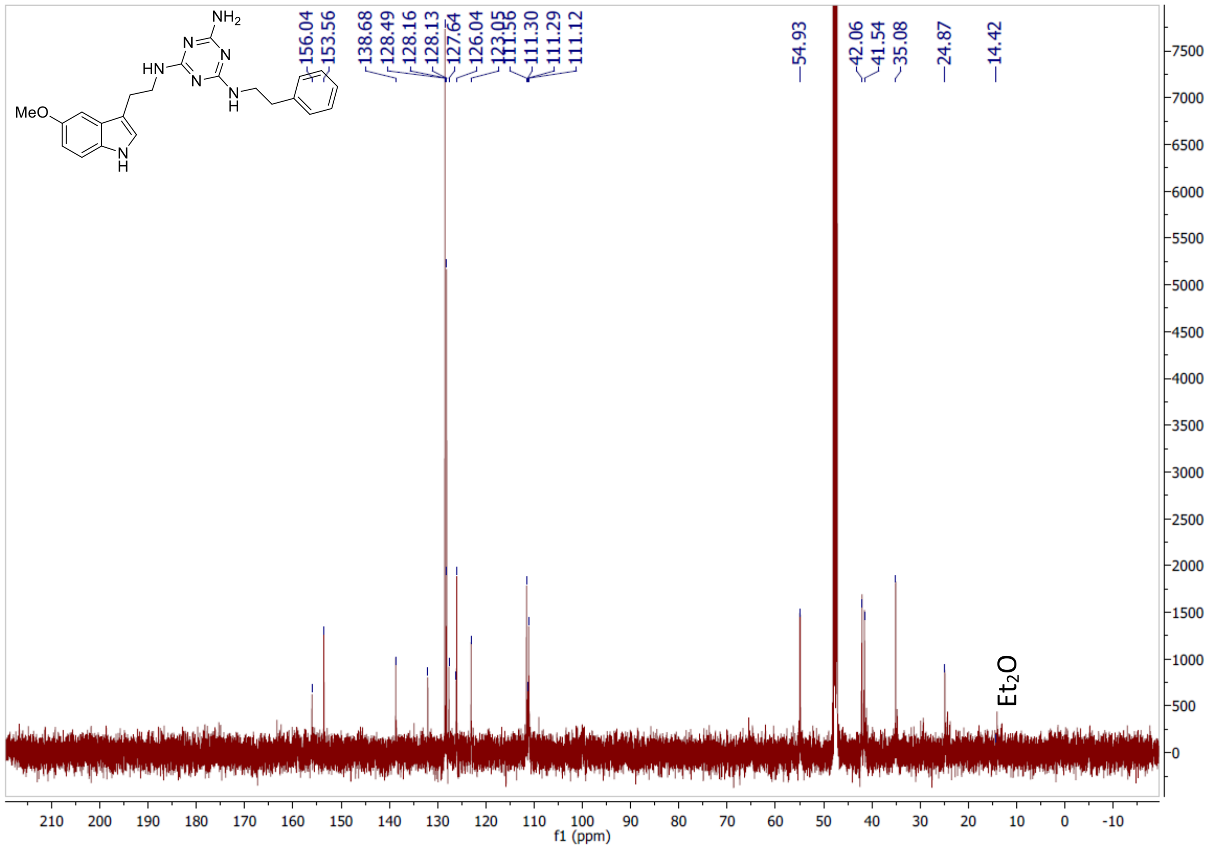

HPLC-MS of compound **6**

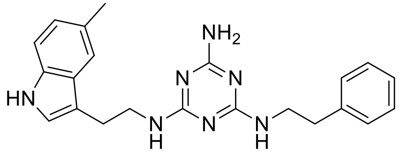

Chemical Formula: $C_{22}H_{25}N_7$
Exact Mass: 387,2

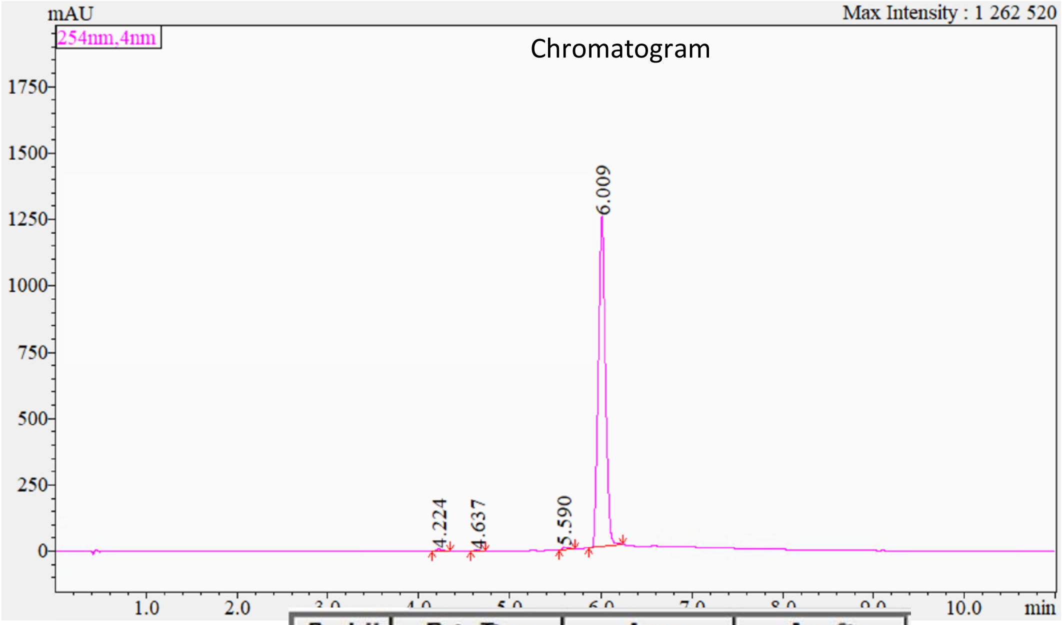

Peak#	Ret. Time	Area	Area%
1	4.224	40571	0.591
2	4.637	25790	0.375
3	5.590	48299	0.703
4	6.009	6753524	98.331
Total		6868184	100.000

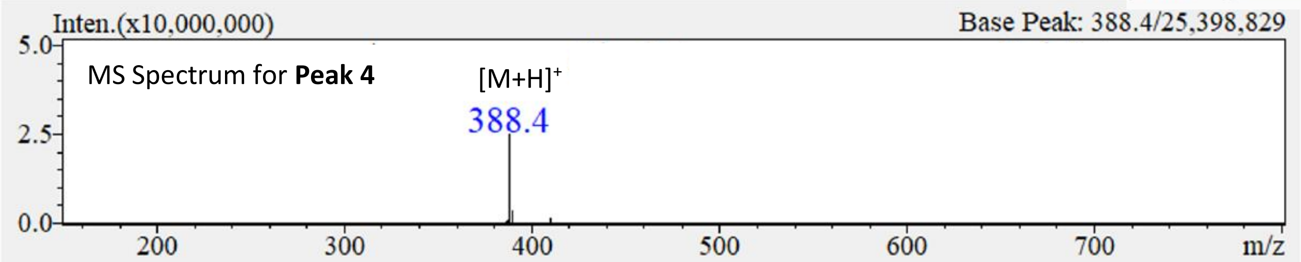

^{1}H NMR of compound **6** in CD_3OD

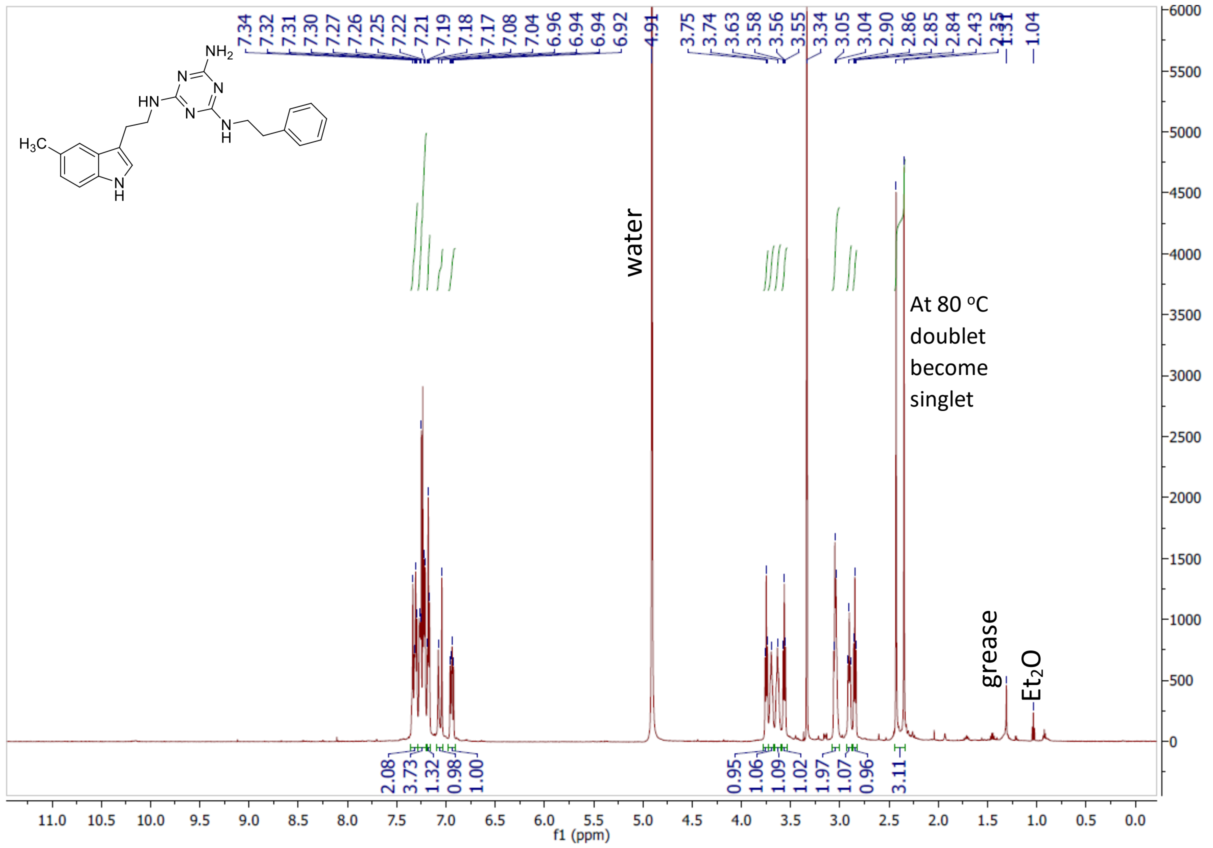

^{13}C NMR of compound **6** in CD_3OD

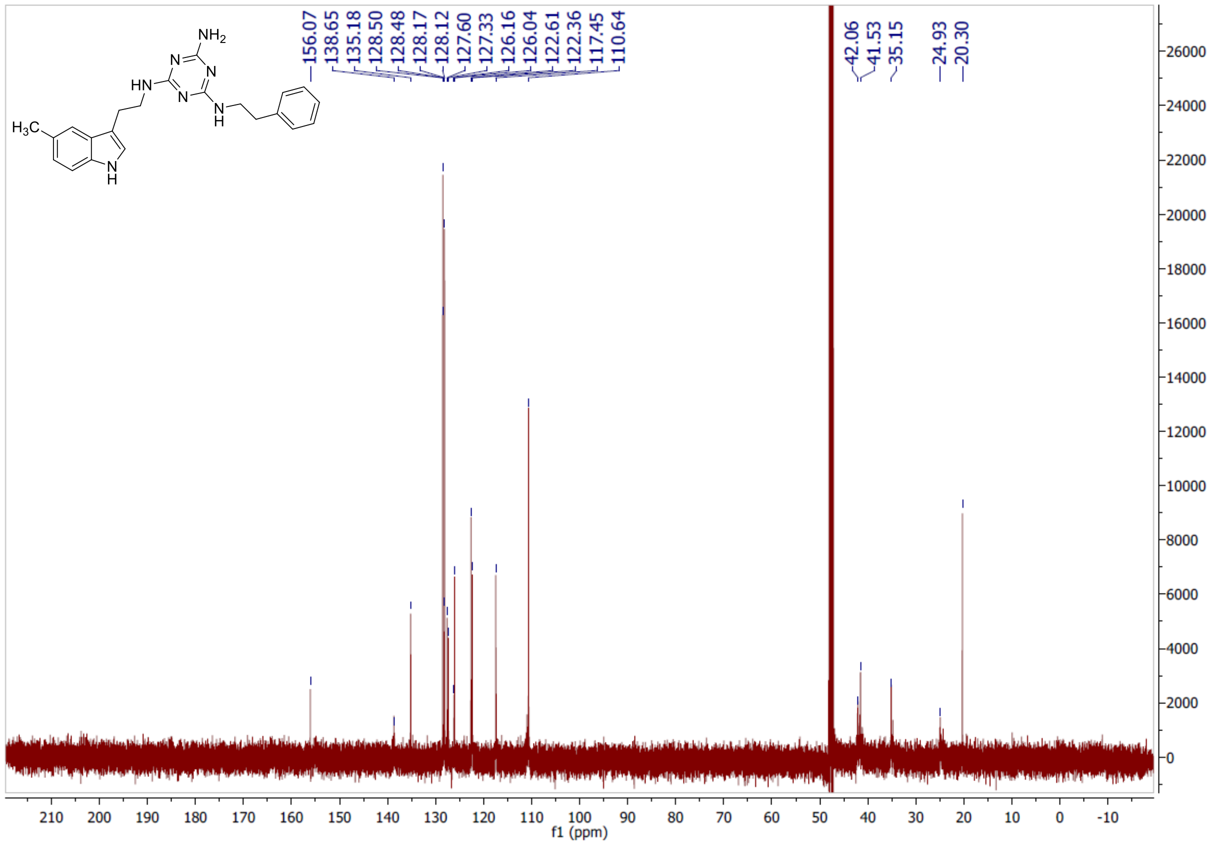

HPLC-MS of compound **7**

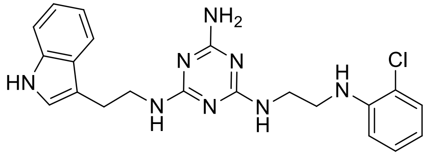

Chemical Formula: $C_{21}H_{23}{}^{35}ClN_8$
Exact Mass: 422,2

Chemical Formula: $C_{21}H_{23}{}^{37}ClN_8$
Exact Mass: 424,2

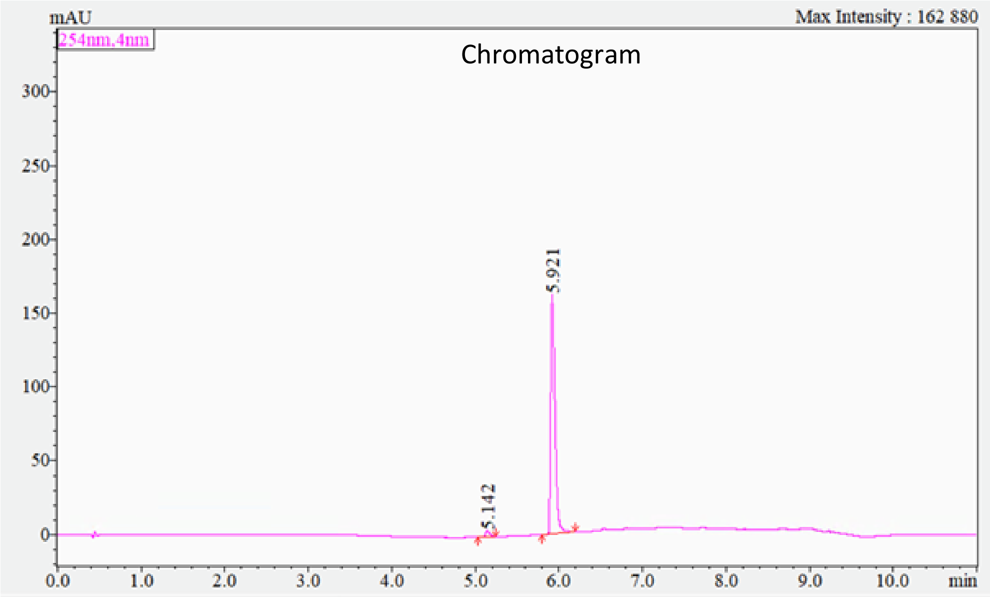

Peak#	Ret. Time	Area	Area%
1	5.142	14143	2.346
2	5.921	588825	97.654
Total		602969	100.000

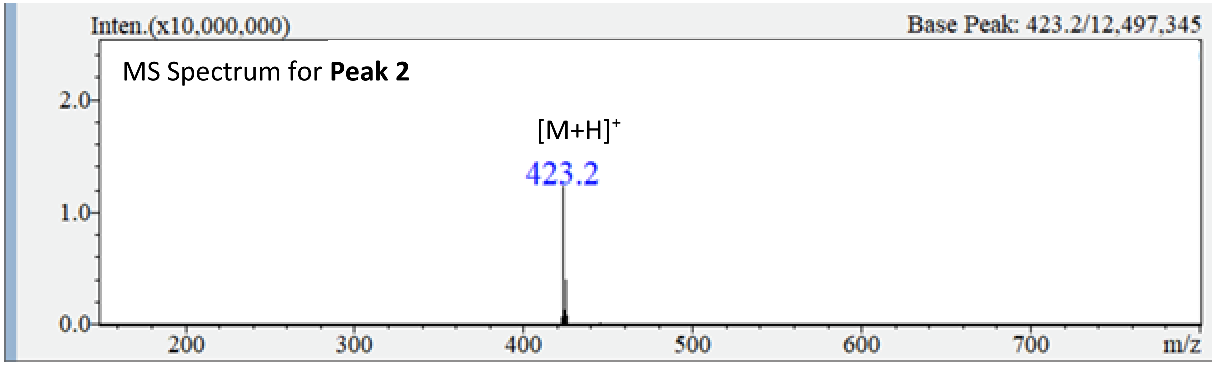

^{1}H NMR of compound **7** in CD_3OD

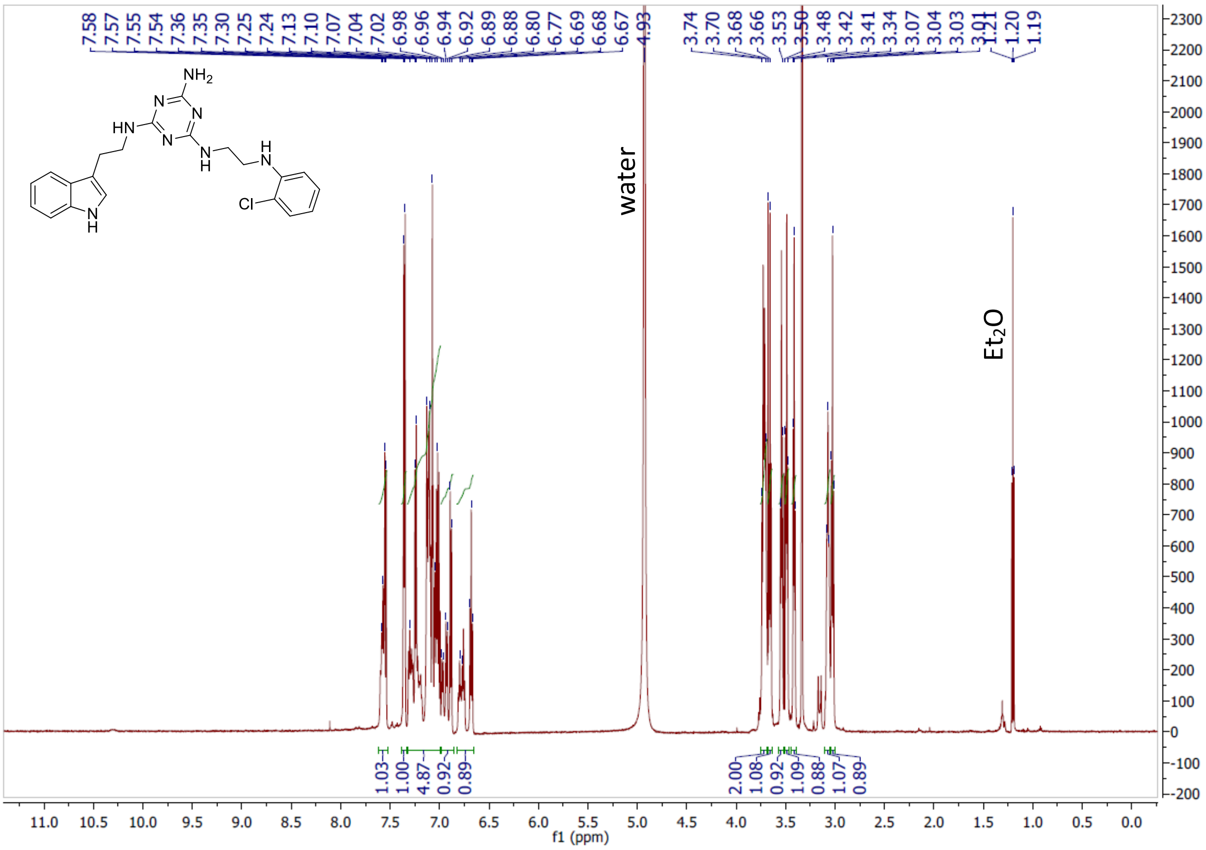

^{13}C NMR of compound **7** in CD_3OD

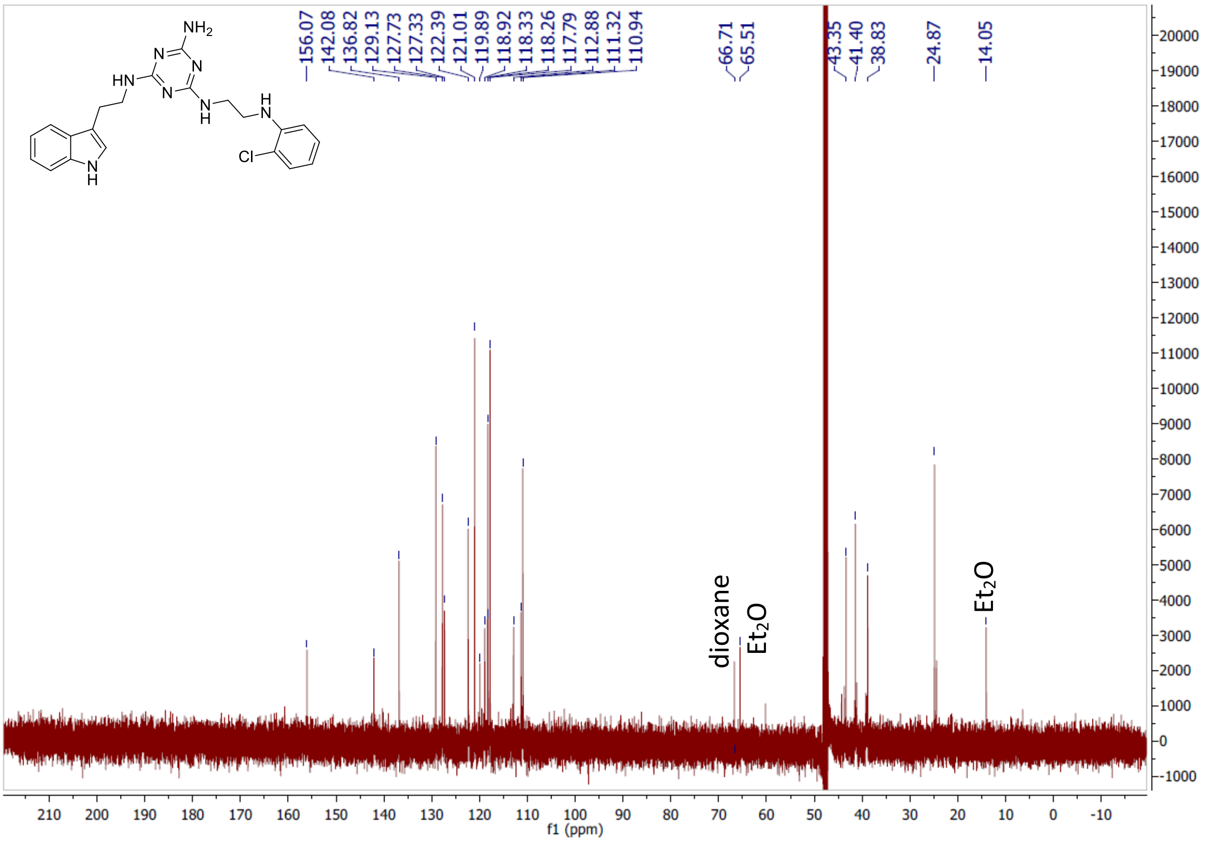

HPLC-MS of compound **8**

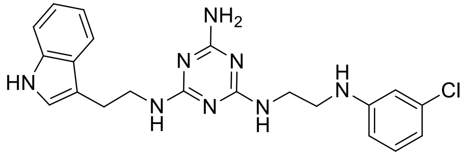

Chemical Formula: $C_{21}H_{23}{}^{35}ClN_8$
Exact Mass: 422,2

Chemical Formula: $C_{21}H_{23}{}^{37}ClN_8$
Exact Mass: 424,2

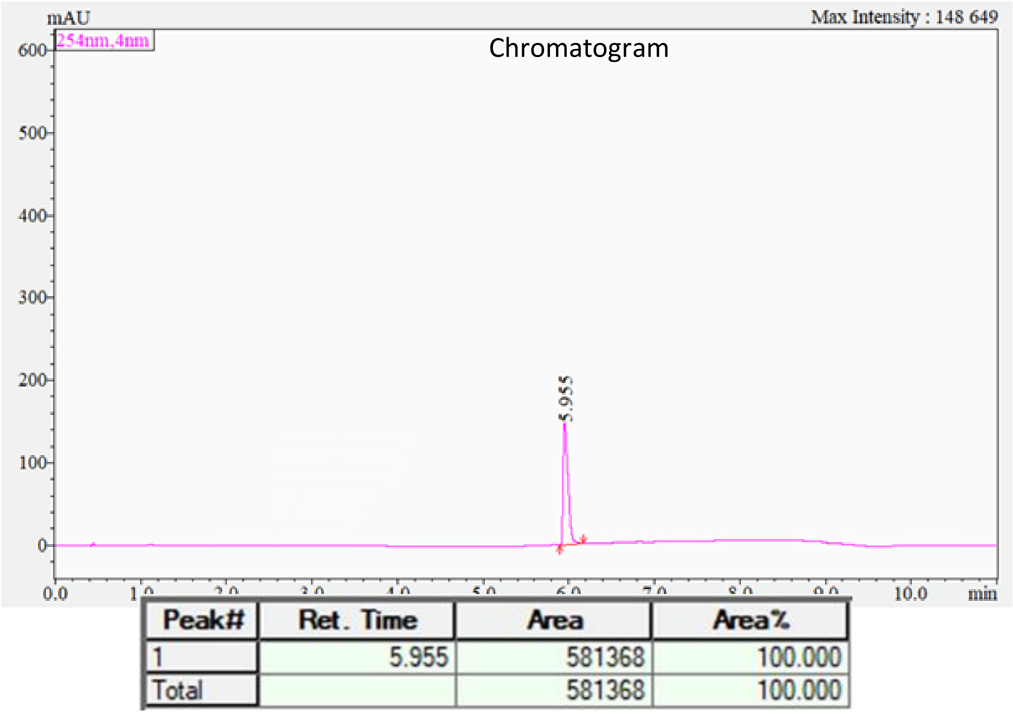

Peak#	Ret. Time	Area	Area%
1	5.955	581368	100.000
Total		581368	100.000

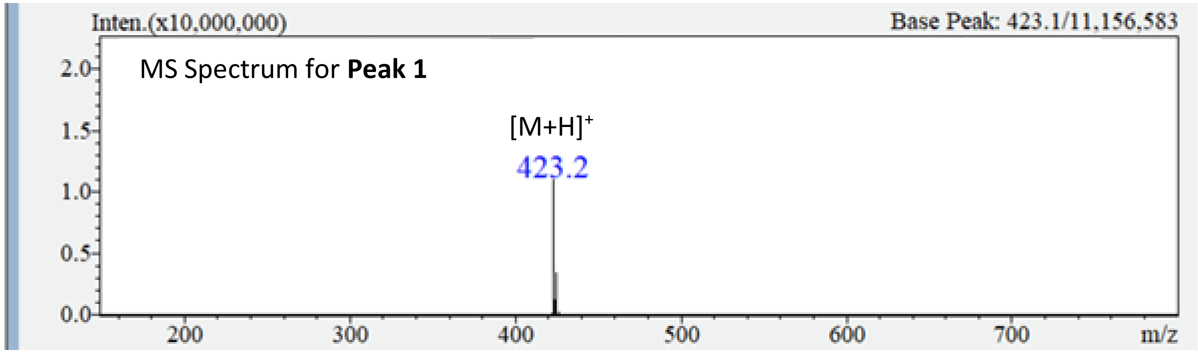

^{1}H NMR of compound **8** in CD_3OD

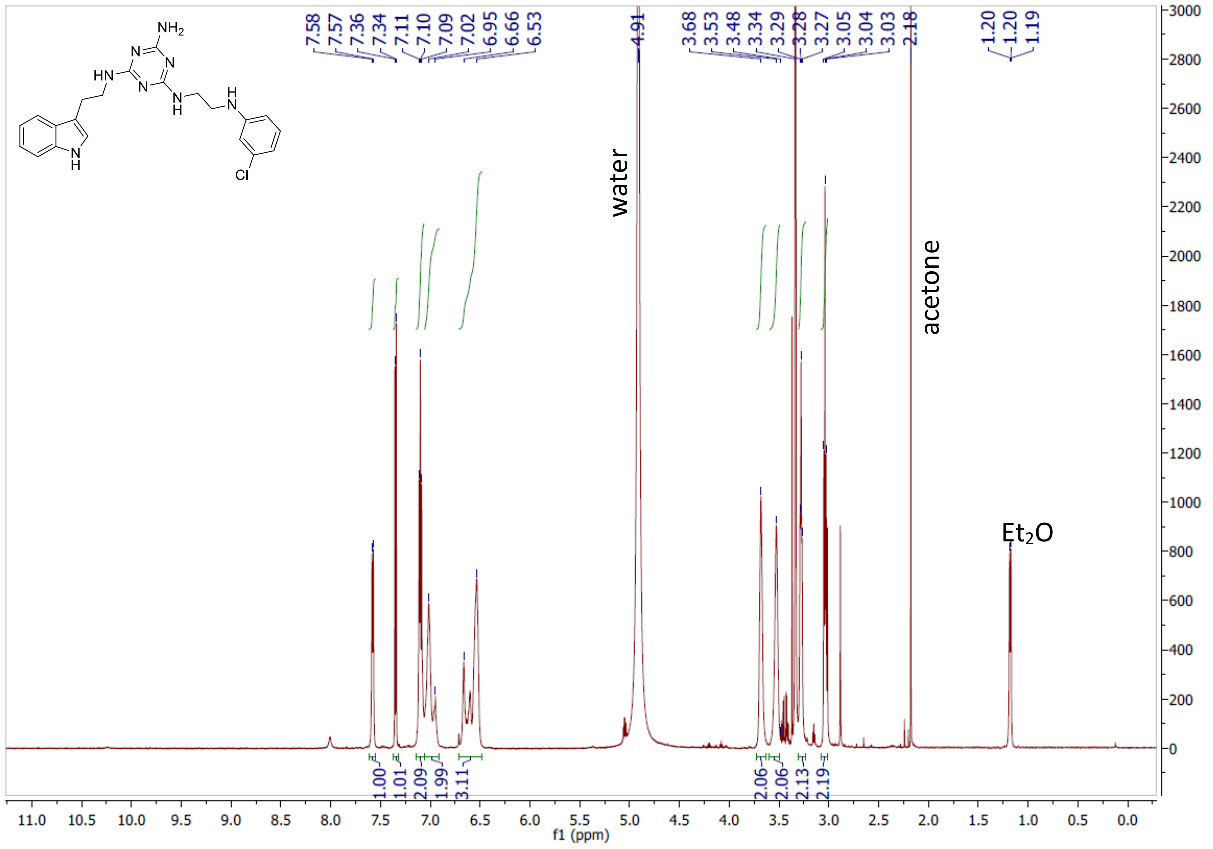

^{13}C NMR of compound **8** in CD_3OD

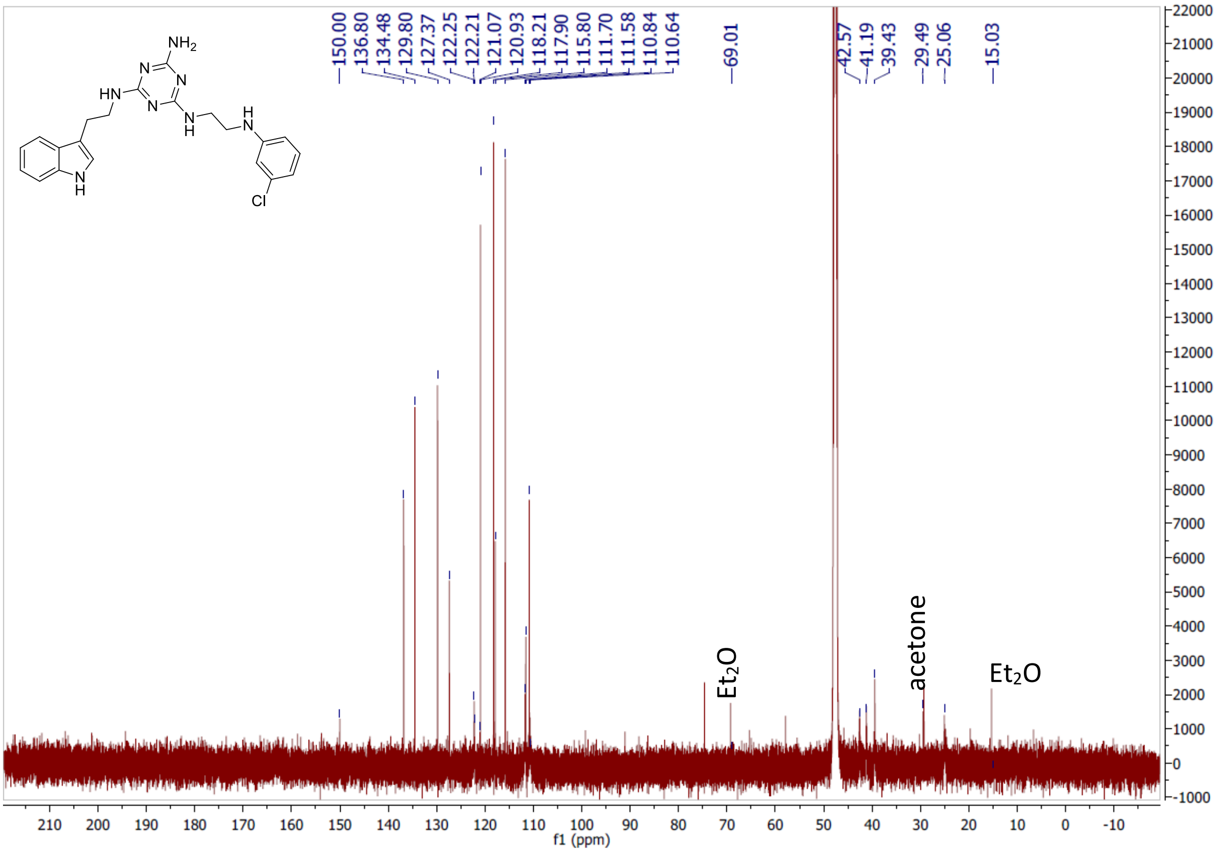

HPLC-MS of compound **9**

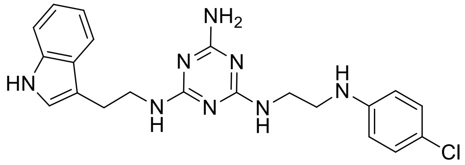

Chemical Formula: $C_{21}H_{23}{}^{35}ClN_8$
Exact Mass: 422,2

Chemical Formula: $C_{21}H_{23}{}^{37}ClN_8$
Exact Mass: 424,2

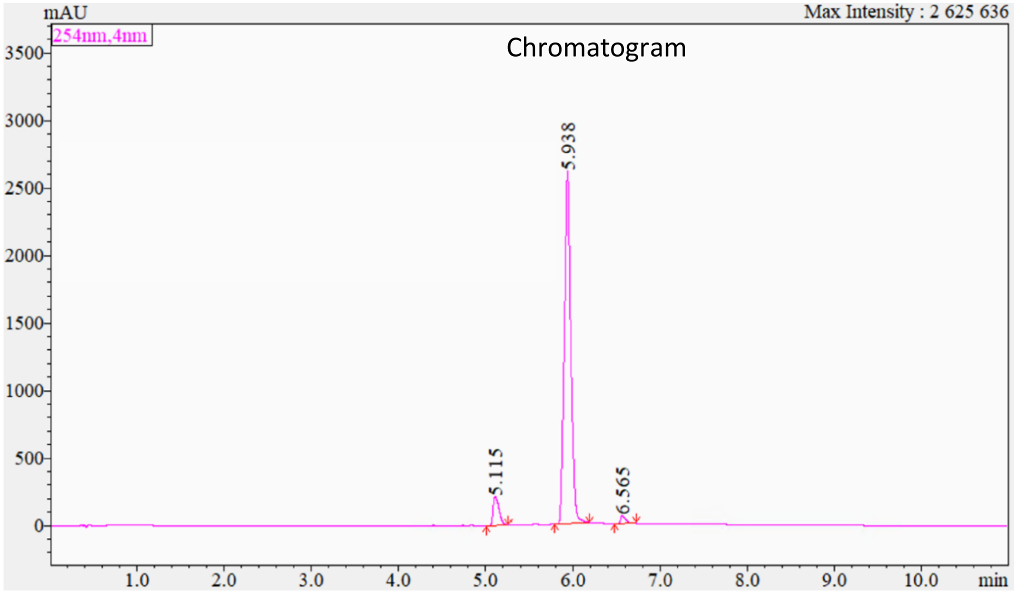

Peak#	Ret. Time	Area	Area%
1	5.115	999078	6.841
2	5.938	13374671	91.584
3	6.565	230031	1.575
Total		14603779	100.000

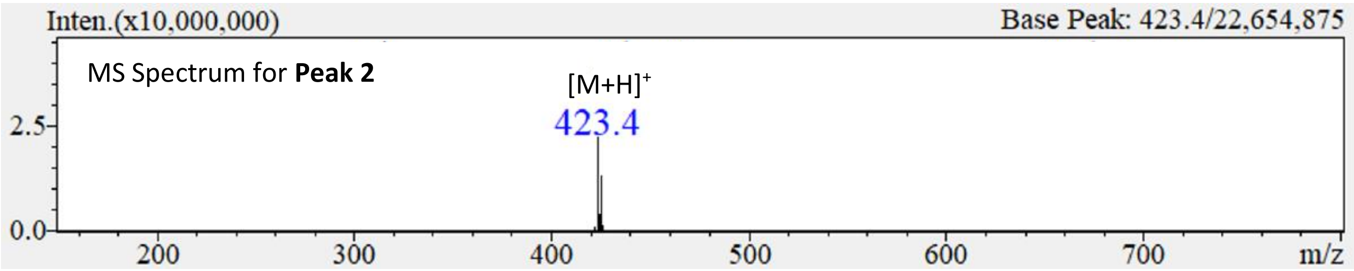

^1H NMR of compound **9** in CD_3OD

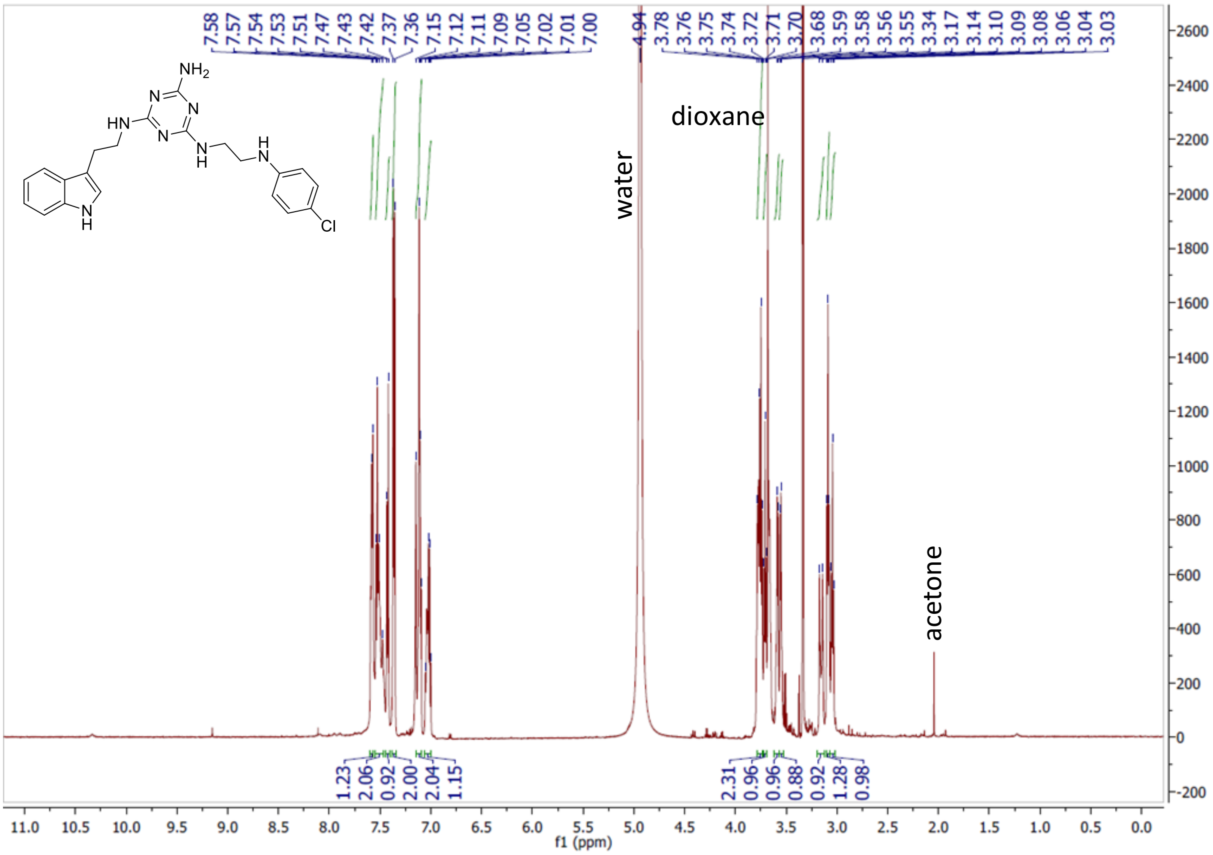

^{13}C NMR of compound **9** in CD_3OD

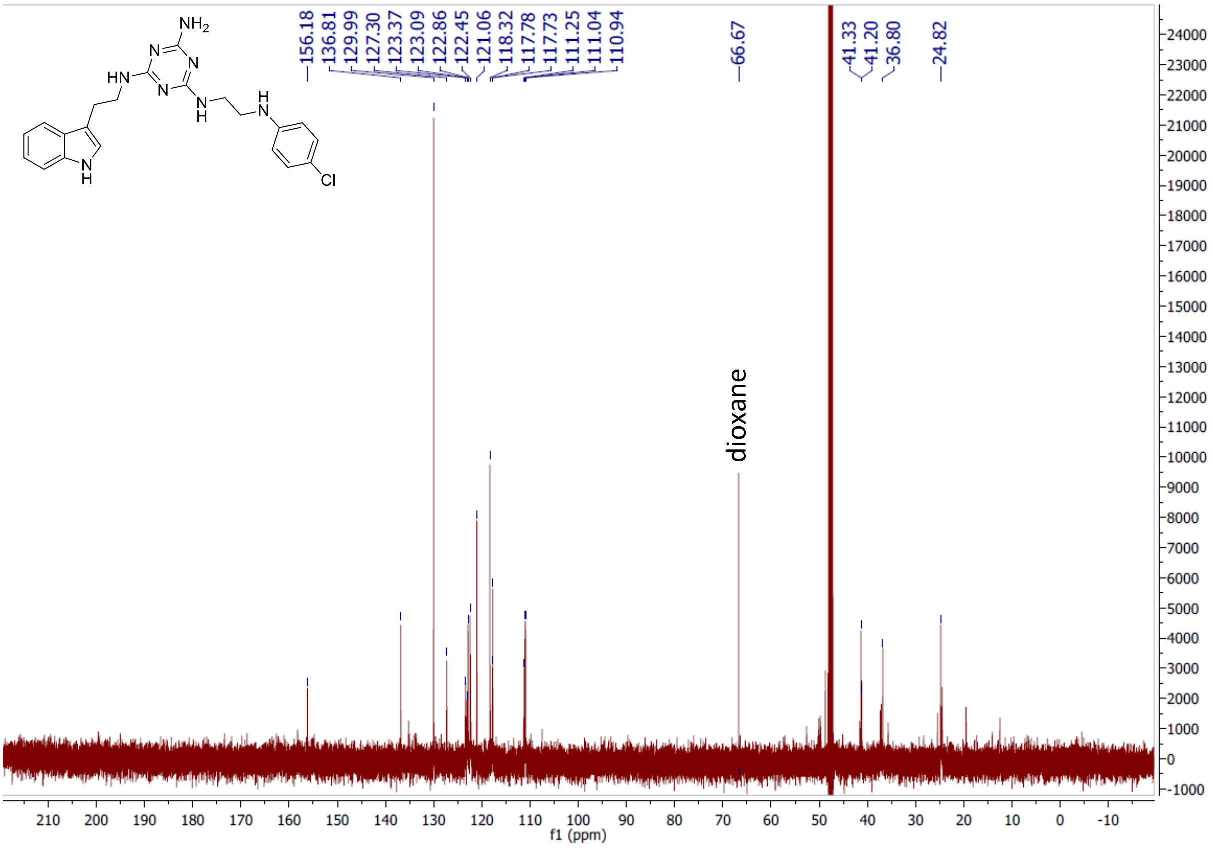

HPLC-MS of compound **10**

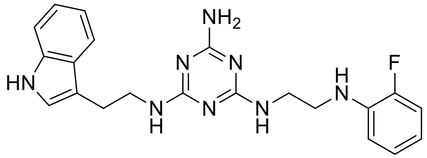

Chemical Formula: $C_{21}H_{23}FN_8$
Exact Mass: 406,2

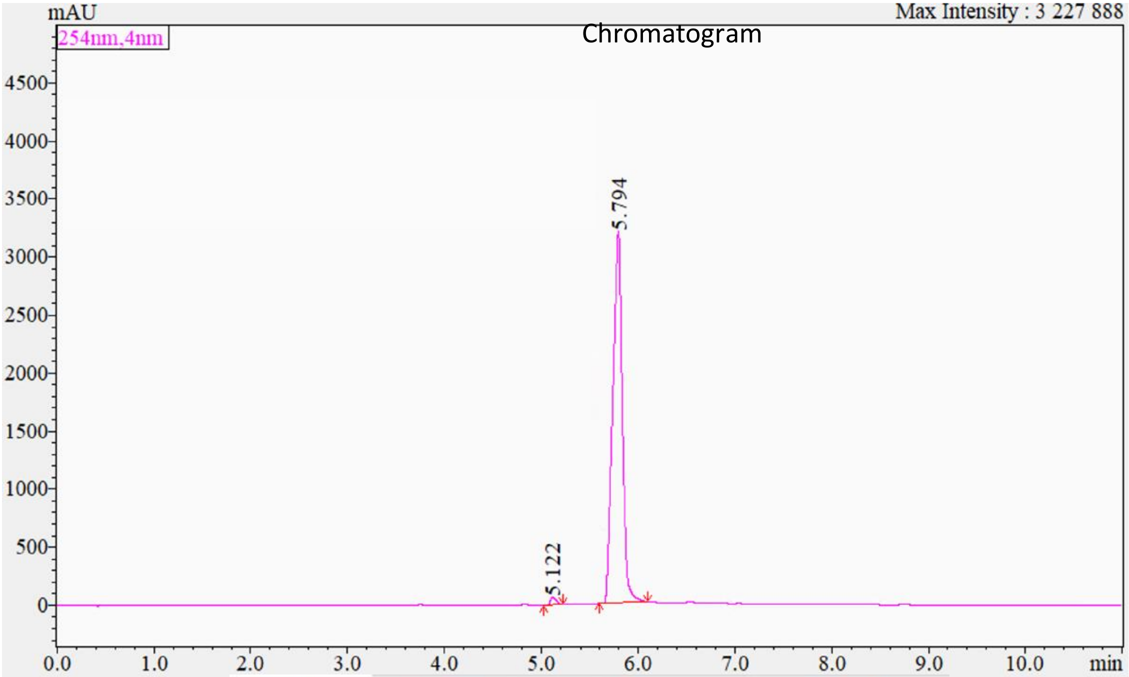

Peak#	Ret. Time	Area	Area%
1	5.122	275139	1.234
2	5.794	22026225	98.766
Total		22301364	100.000

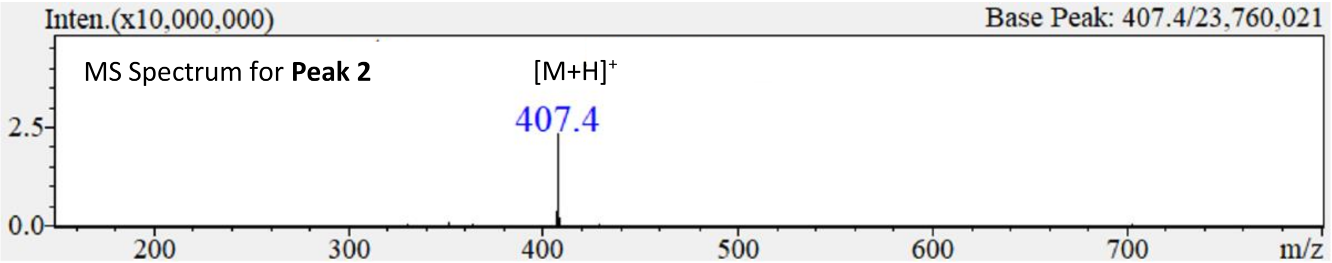

^{1}H NMR of compound **10** in CD_3OD

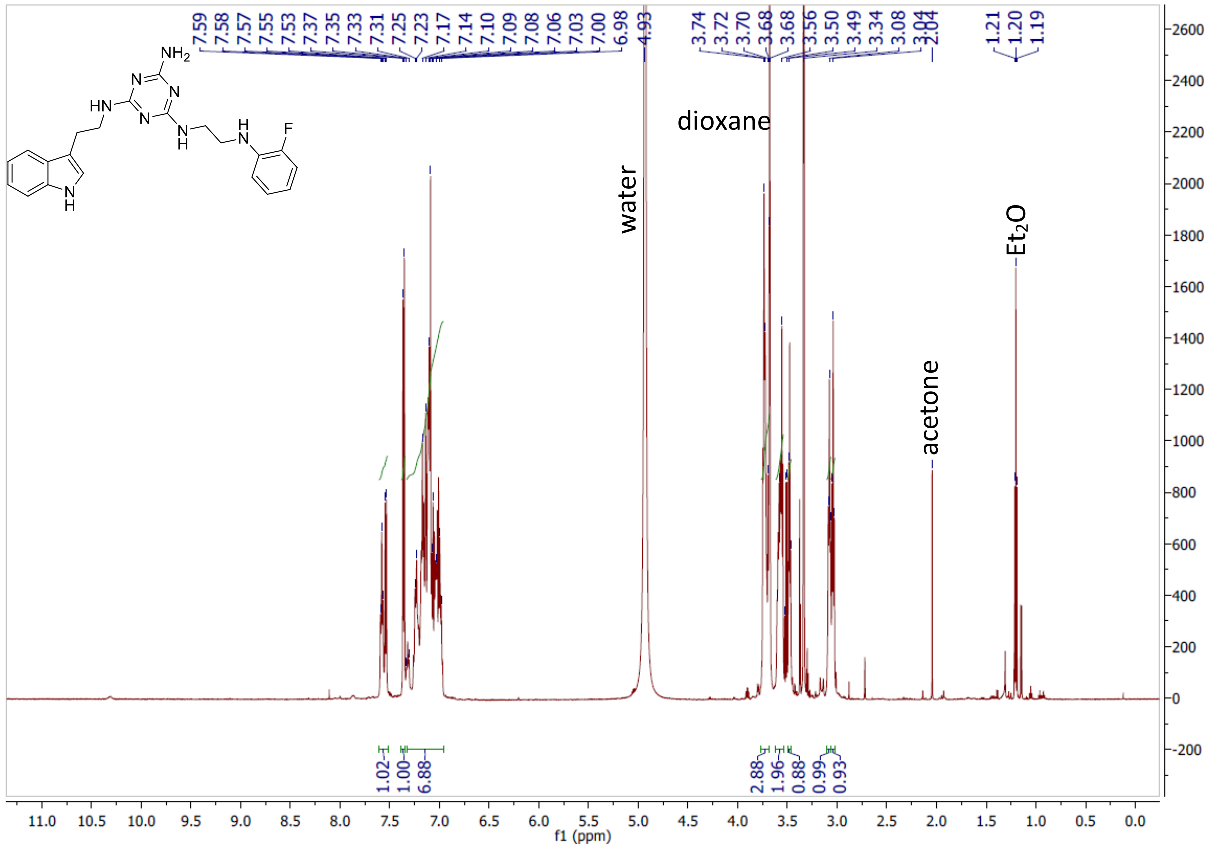

^{13}C NMR of compound **10** in CD_3OD

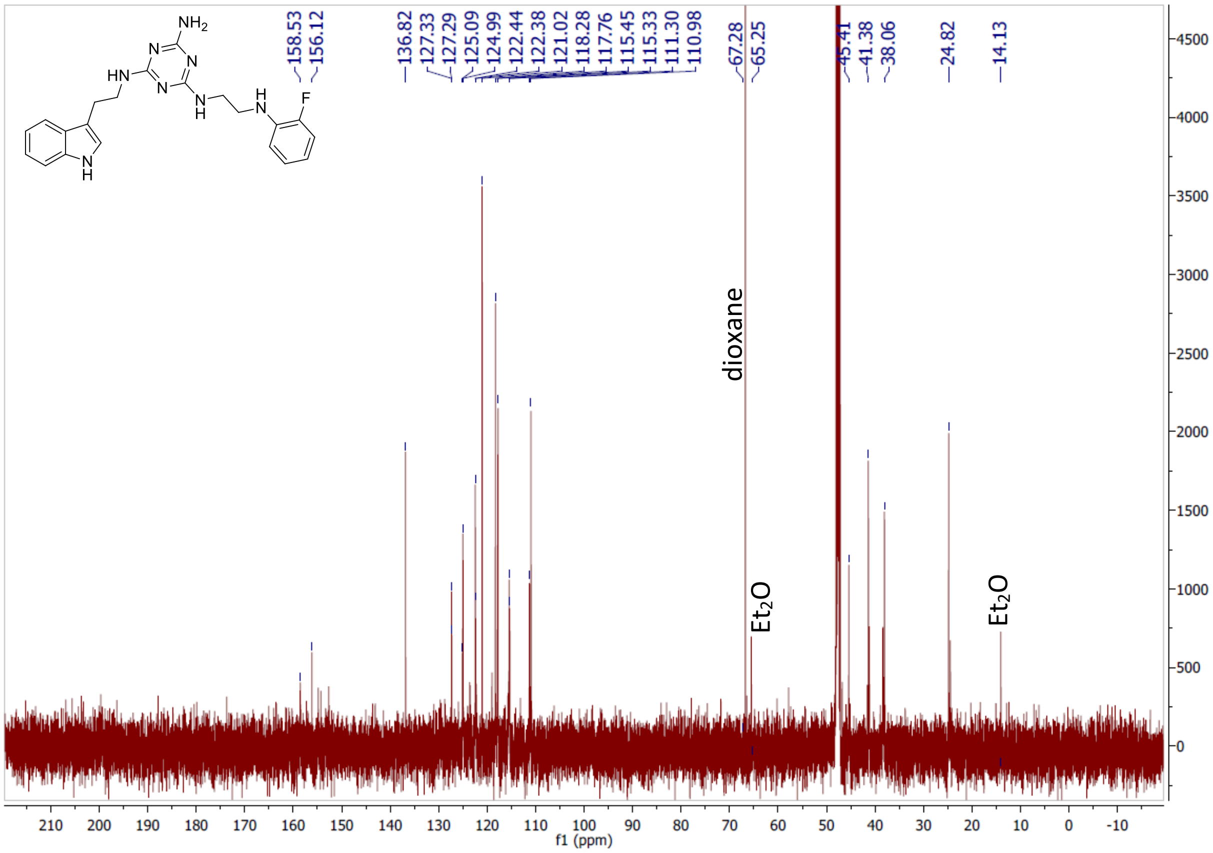

HPLC-MS of compound **11**

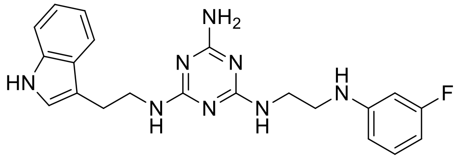

Chemical Formula: $C_{21}H_{23}FN_8$
Exact Mass: 406,2

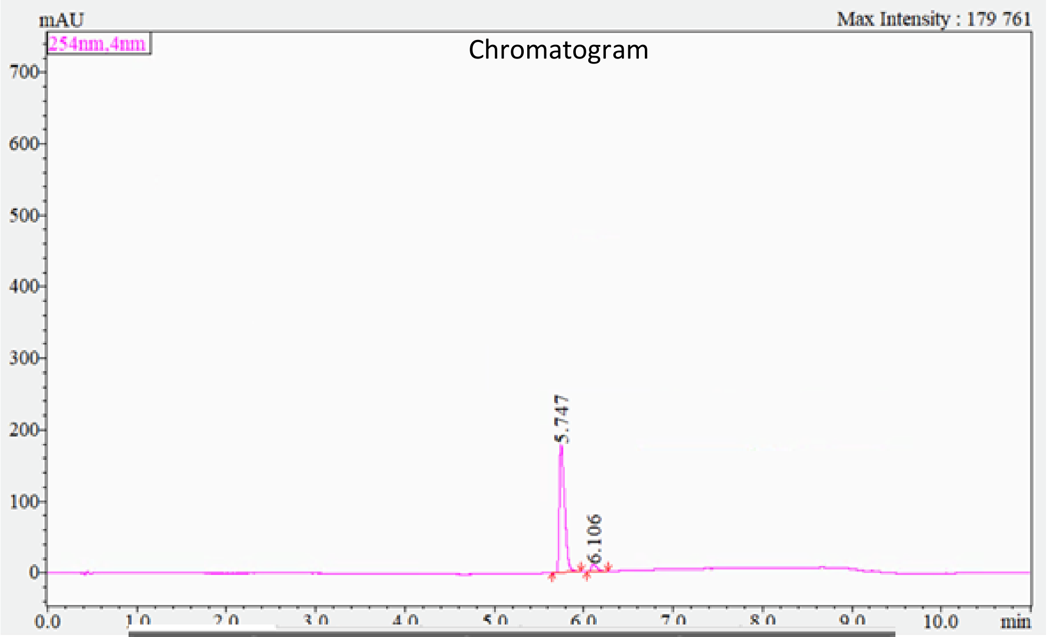

Peak#	Ret. Time	Area	Area%
1	5.747	711555	94.415
2	6.106	42092	5.585
Total		753647	100.000

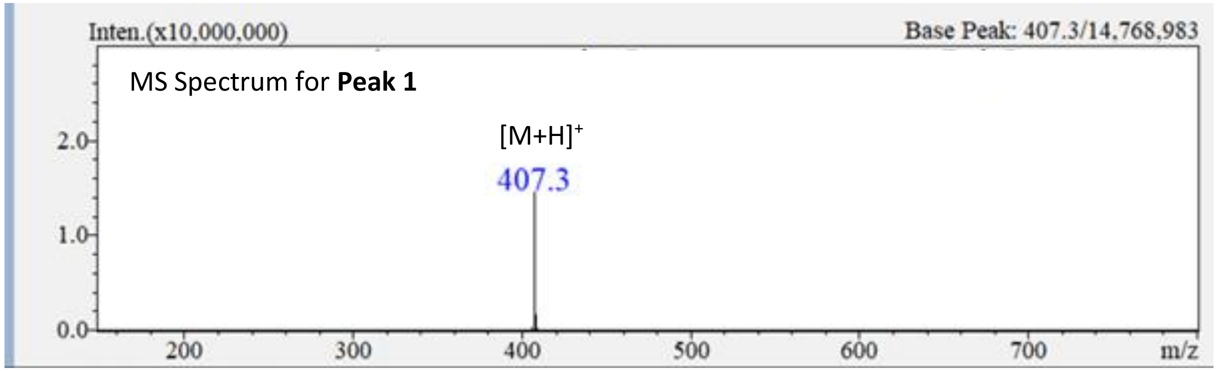

^{1}H NMR of compound **11** in CD_3OD

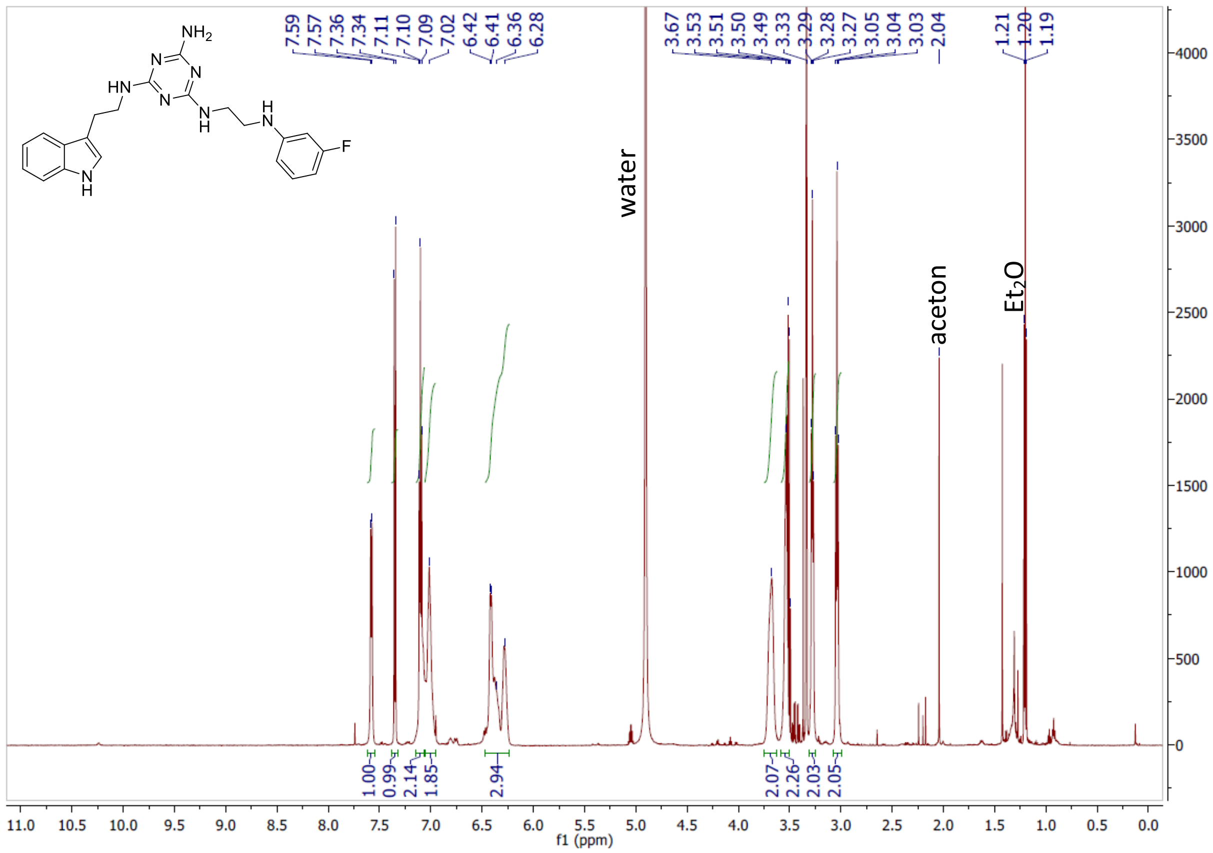

^{13}C NMR of compound **11** in CD_3OD

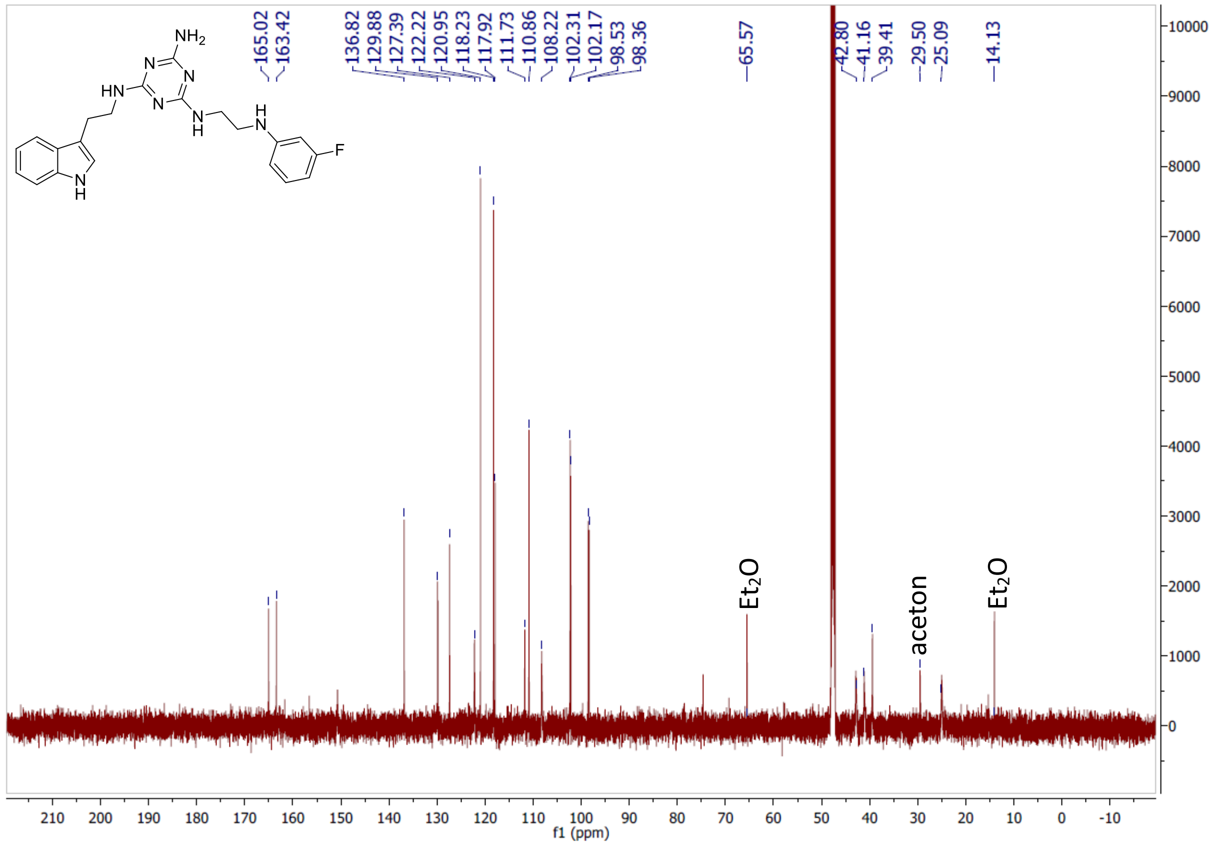

HPLC-MS of compound **12**

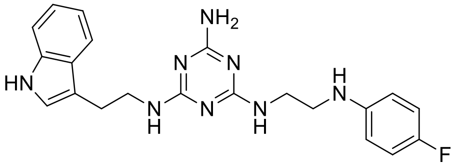

Chemical Formula: $C_{21}H_{23}FN_8$
Exact Mass: 406,2

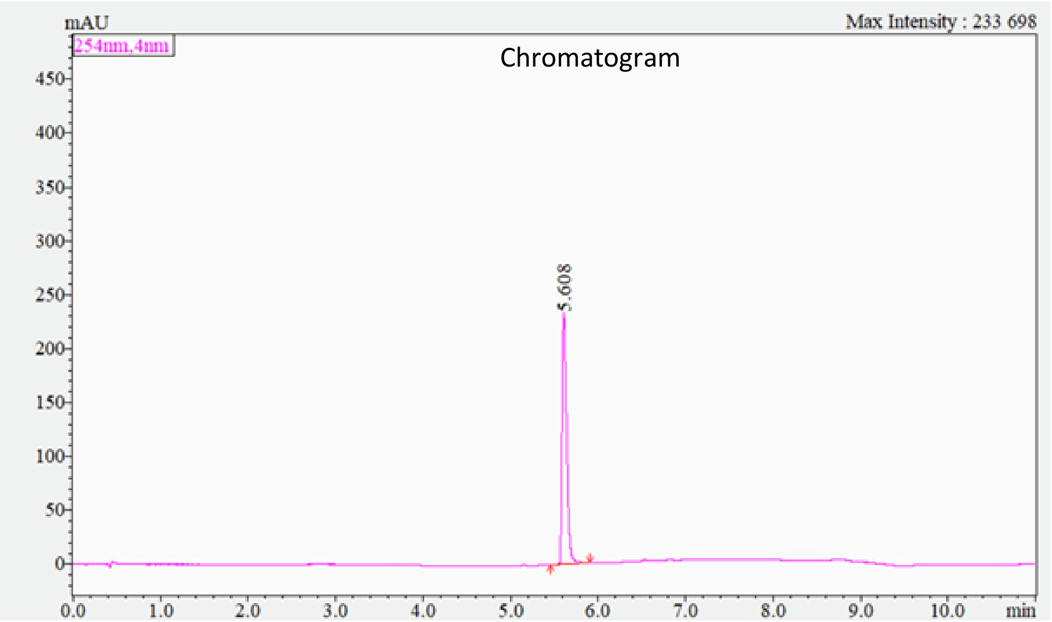

Peak#	Ret. Time	Area	Area%
1	5.608	867759	100.000
Total		867759	100.000

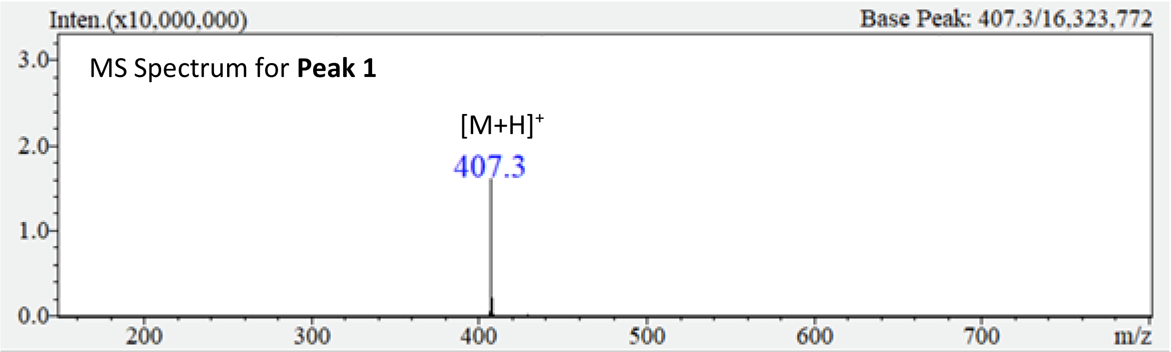

^1H NMR of compound **12** in CD_3OD

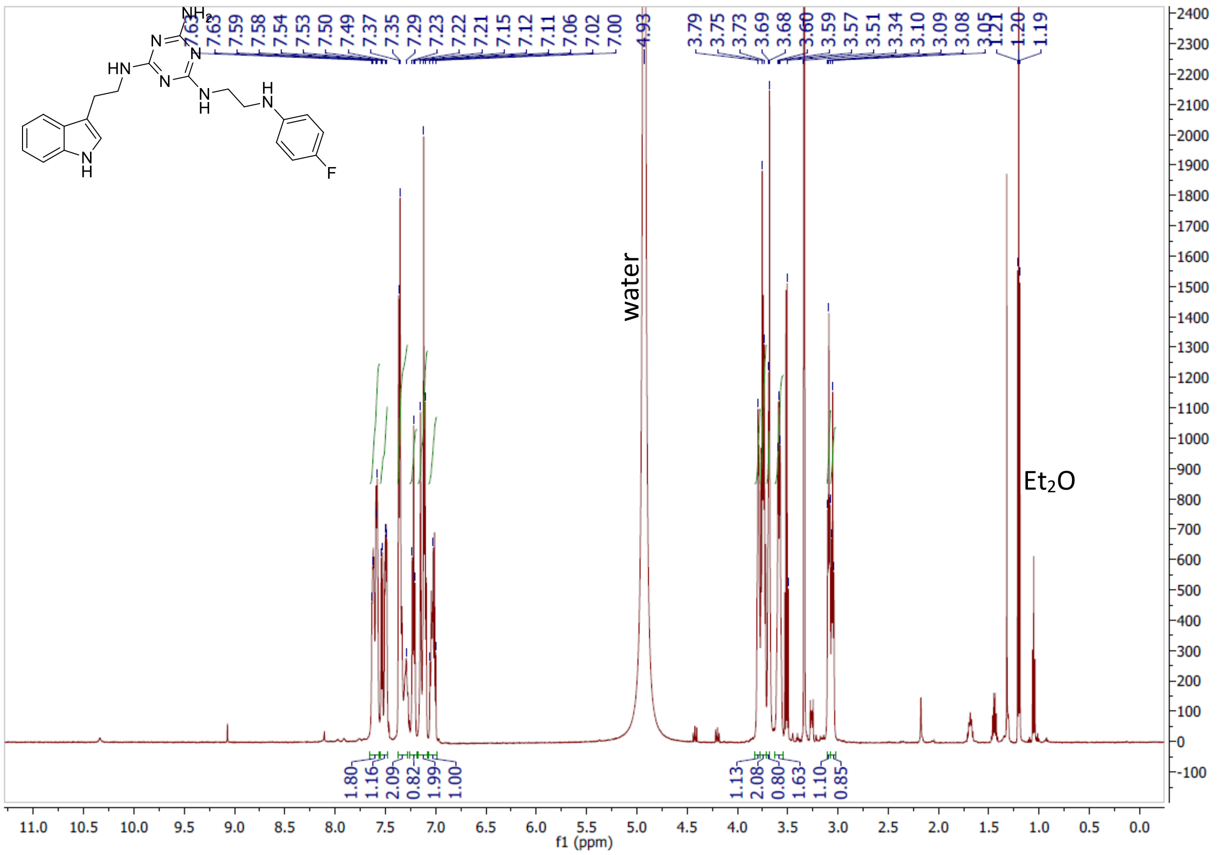

^{13}C NMR of compound **12** in CD_3OD

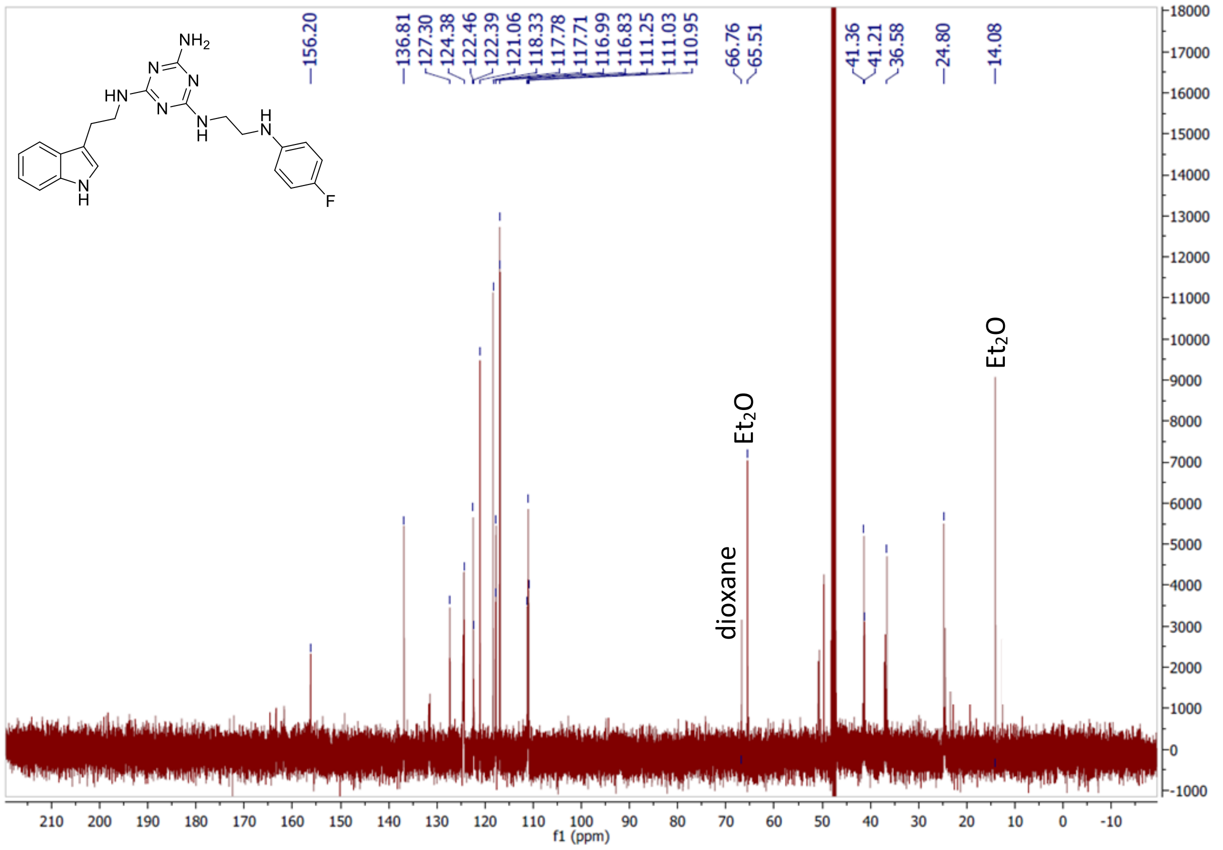

HPLC-MS of compound **14**

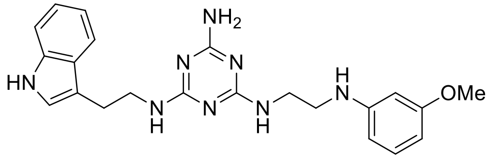

Chemical Formula: $C_{22}H_{26}N_8O$
Exact Mass: 418,2

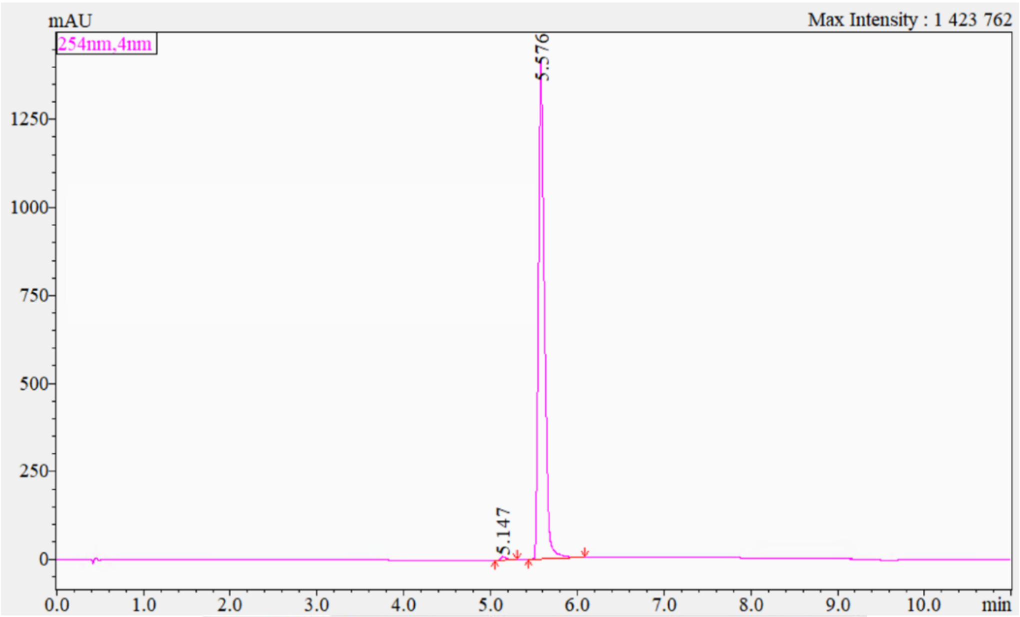

Peak#	Ret. Time	Area	Area%
1	5.147	38162	0.558
2	5.576	6794983	99.442
Total		6833145	100.000

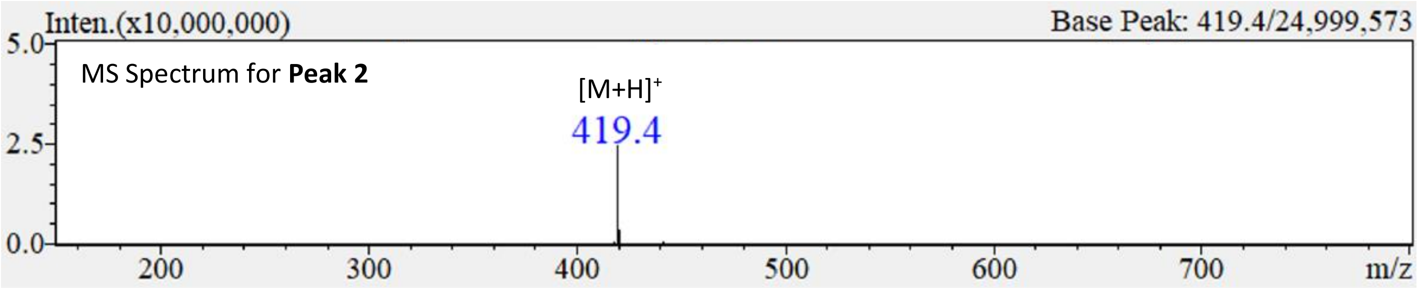

^{1}H NMR of compound **14** in CD_3OD

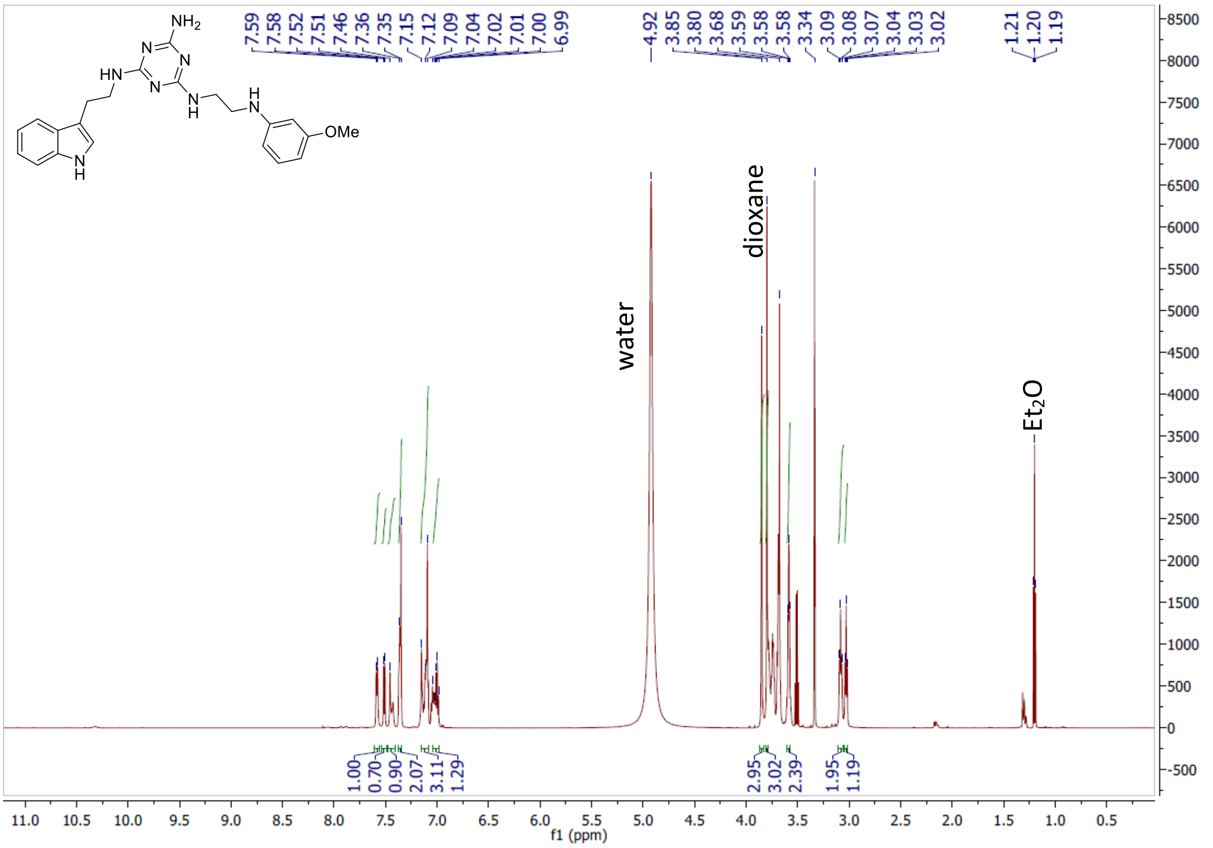

^{13}C NMR of compound **14** in CD_3OD

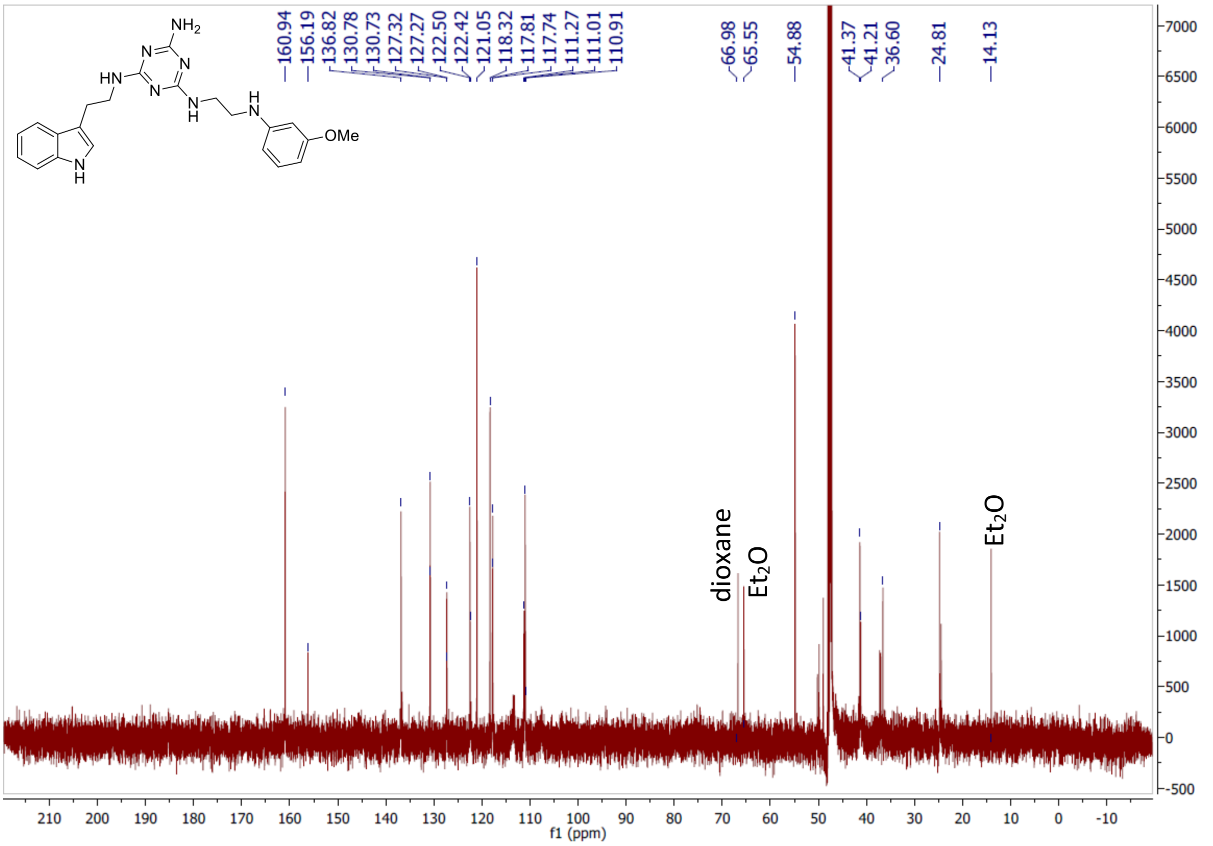

HPLC-MS of compound **15**

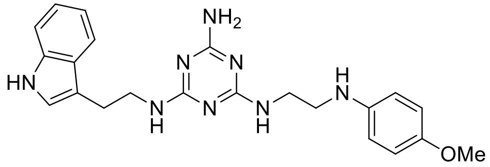

Chemical Formula: $C_{22}H_{26}N_8O$
Exact Mass: 418,2

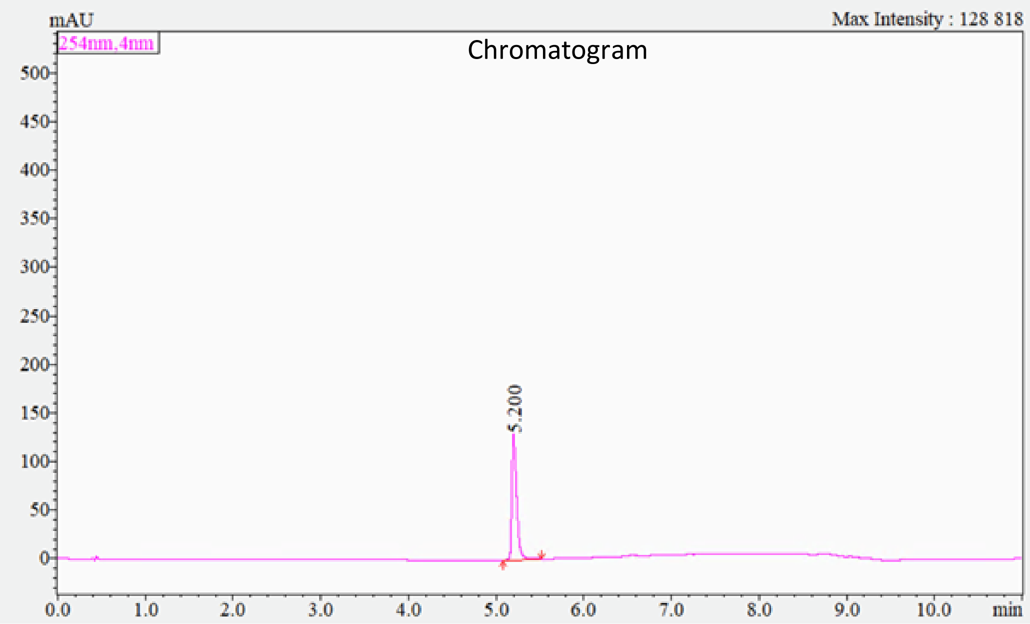

Peak#	Ret. Time	Area	Area%
1	5.200	500861	100.000
Total		500861	100.000

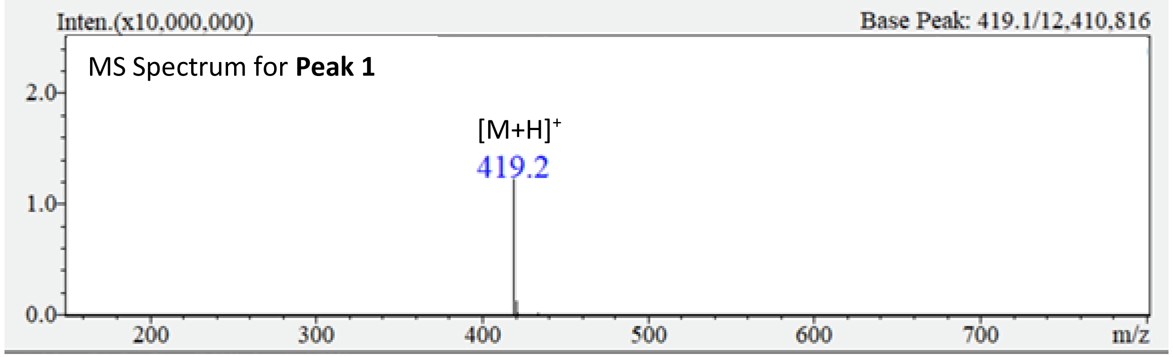

^{1}H NMR of compound **15** in CD_3OD

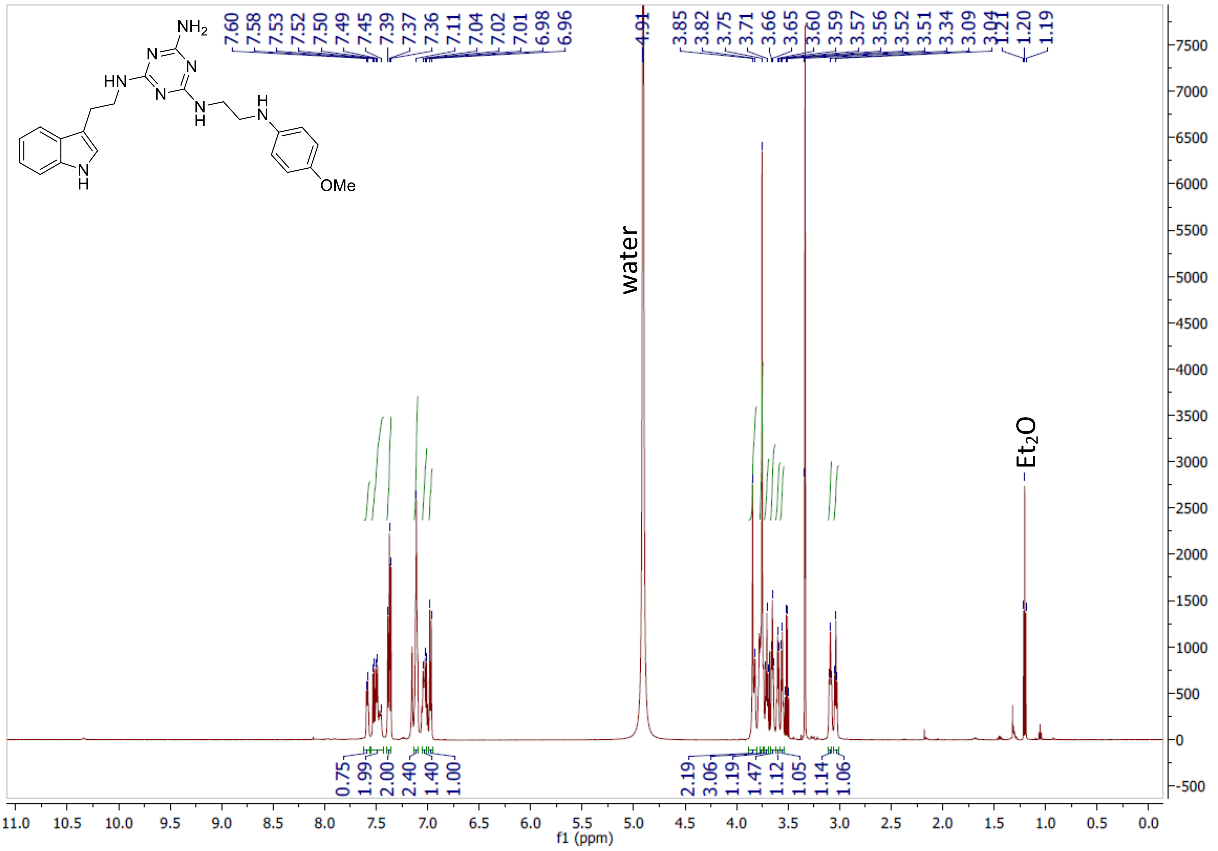

^{13}C NMR of compound **15** in CD_3OD

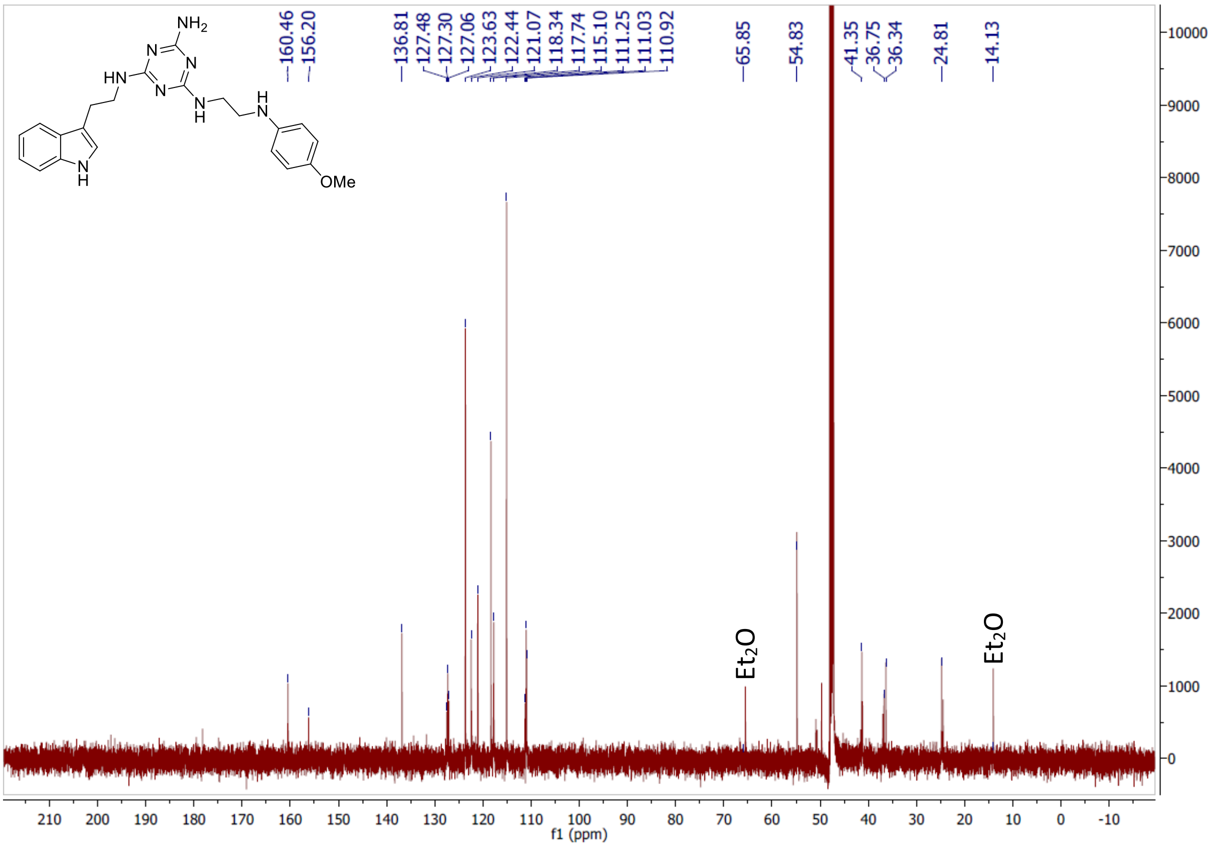

HPLC-MS of compound **16**

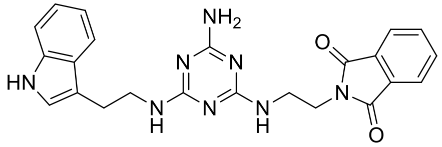

Chemical Formula: $C_{23}H_{22}N_8O_2$
Exact Mass: 442,2

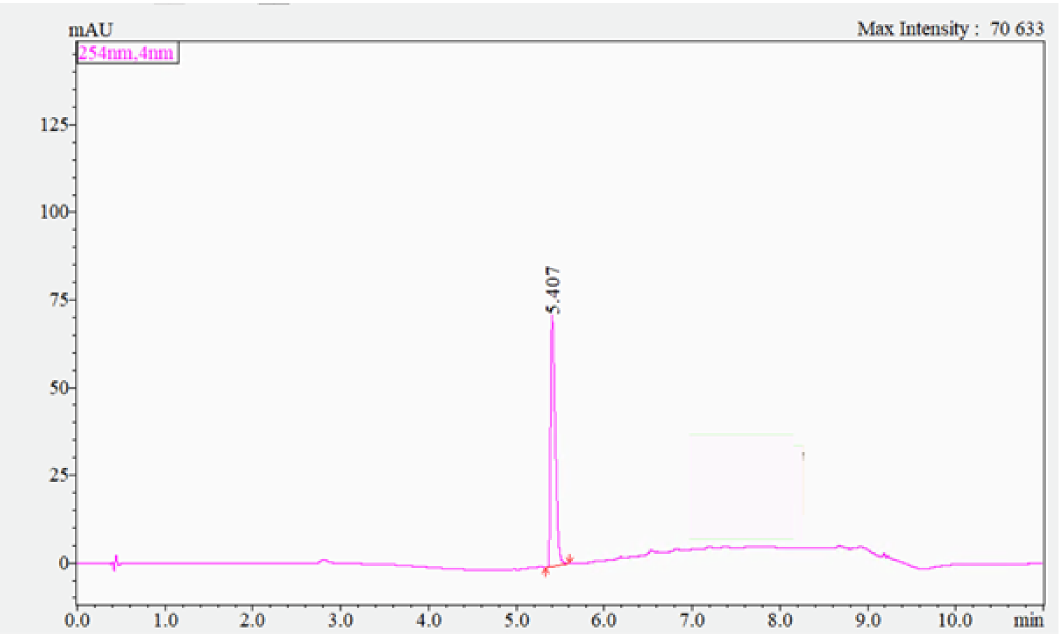

Peak#	Ret. Time	Area	Area%
1	5.407	268024	100.000
Total		268024	100.000

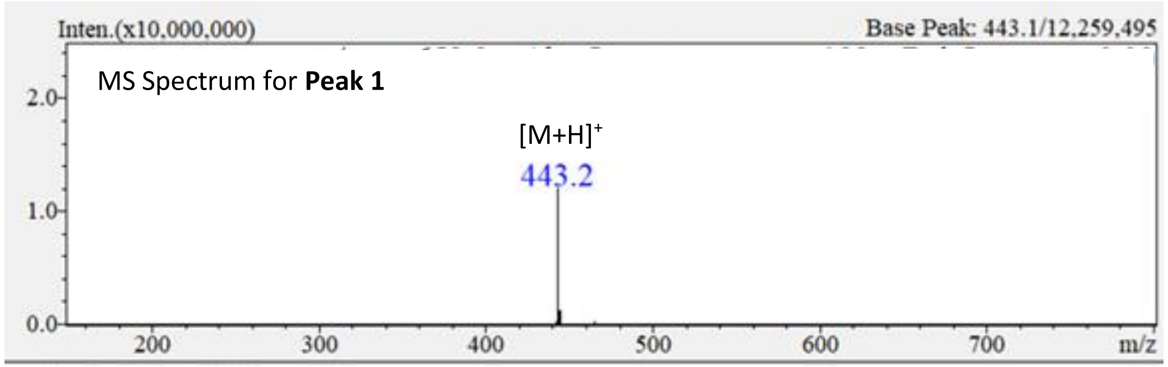

^{1}H NMR of compound **16** in CD_3OD

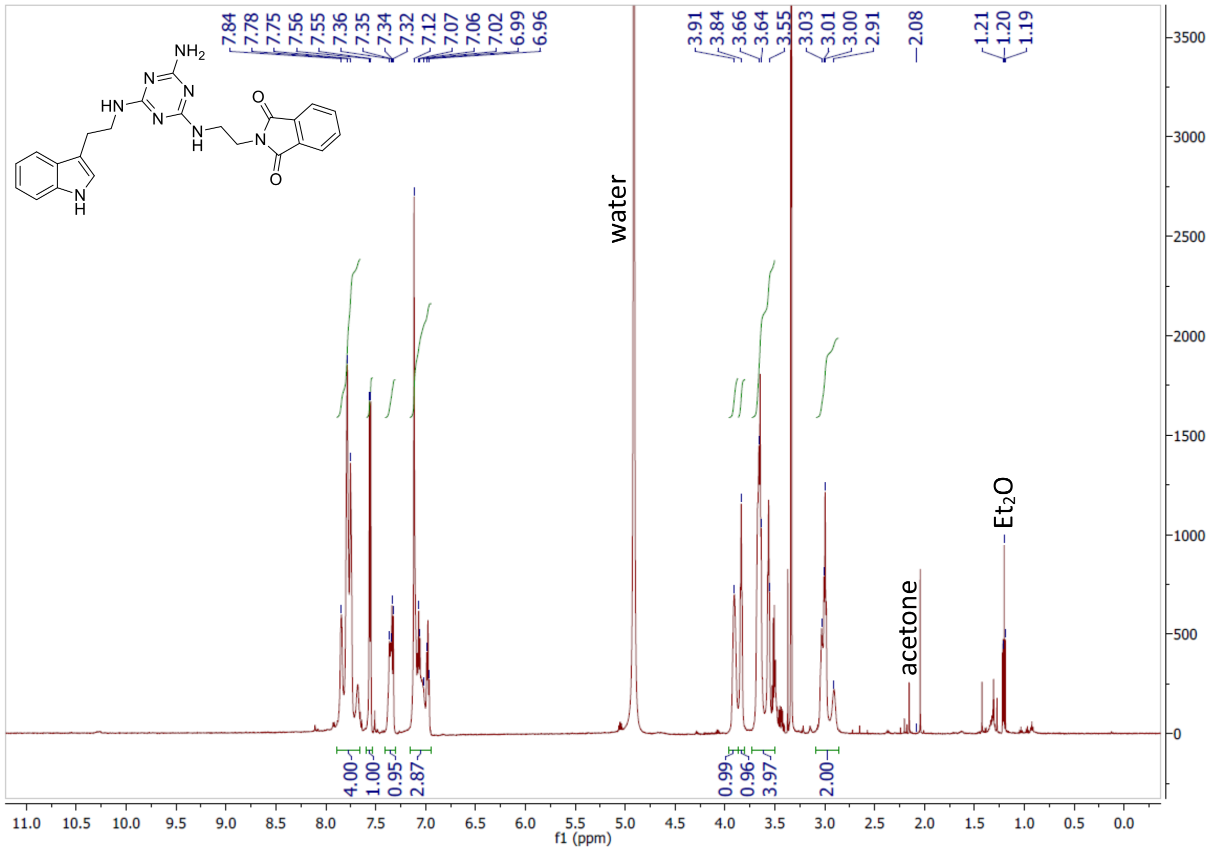

^{13}C NMR of compound **16** in CD_3OD

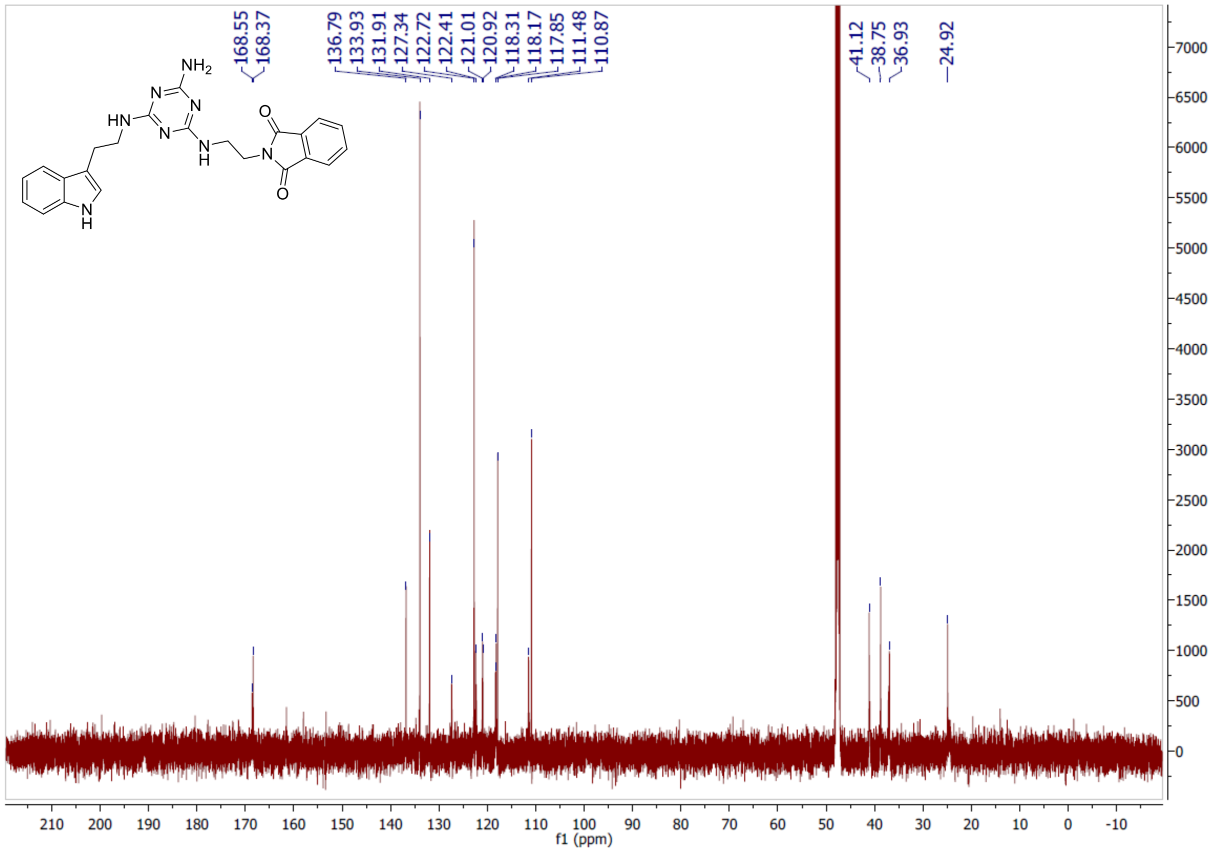

HPLC-MS of compound **17**

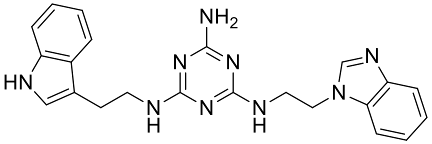

Chemical Formula: $C_{22}H_{23}N_9$
Exact Mass: 413,2

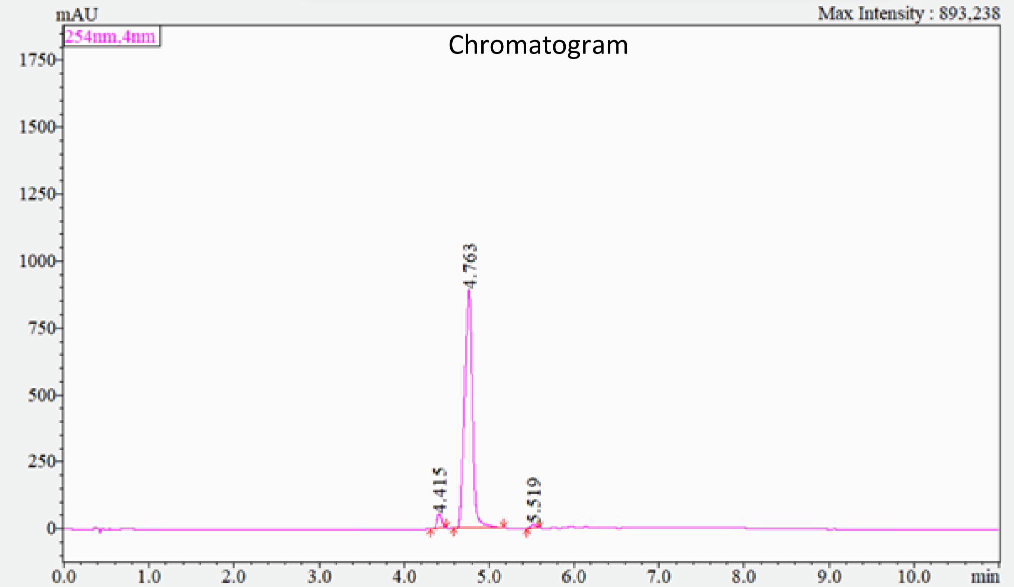

Peak#	Ret. Time	Area	Area%
1	4.415	211077	3.531
2	4.763	5713789	95.589
3	5.519	52615	0.880
Total		5977481	100.000

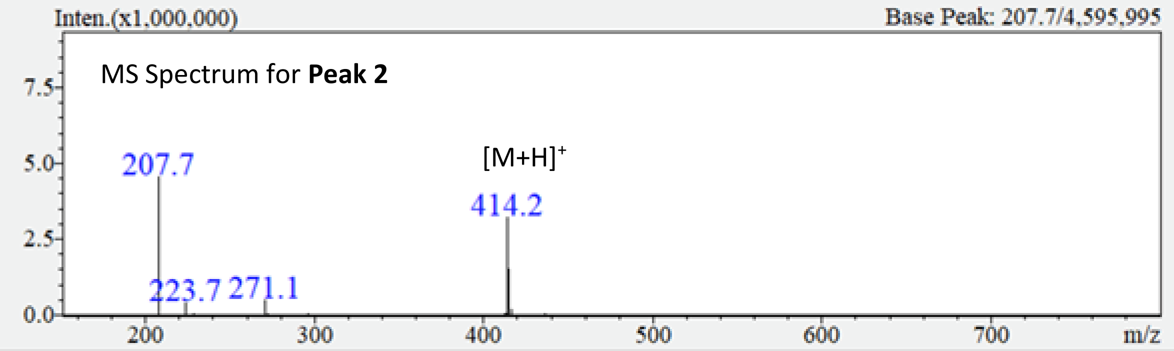

^{1}H NMR of compound **17** in CD_3OD

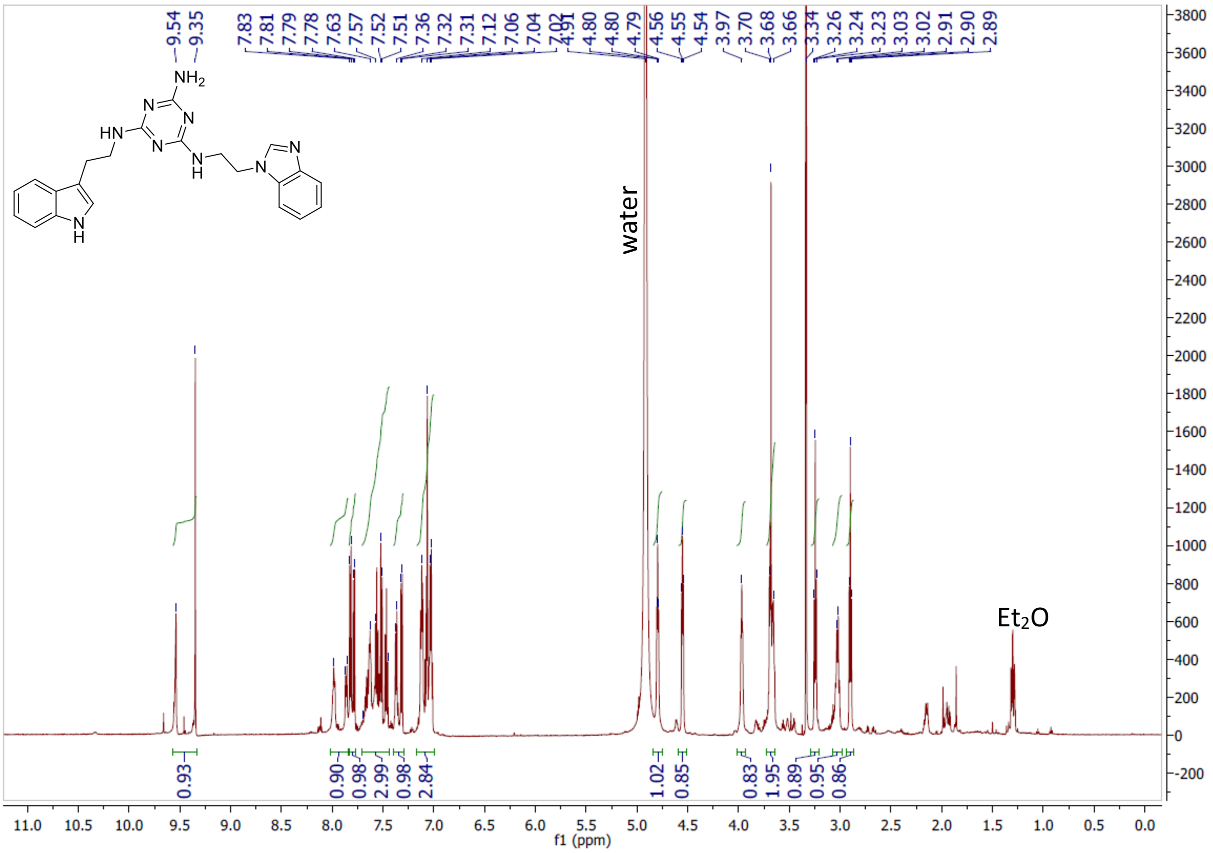

^{13}C NMR of compound **17** in CD_3OD

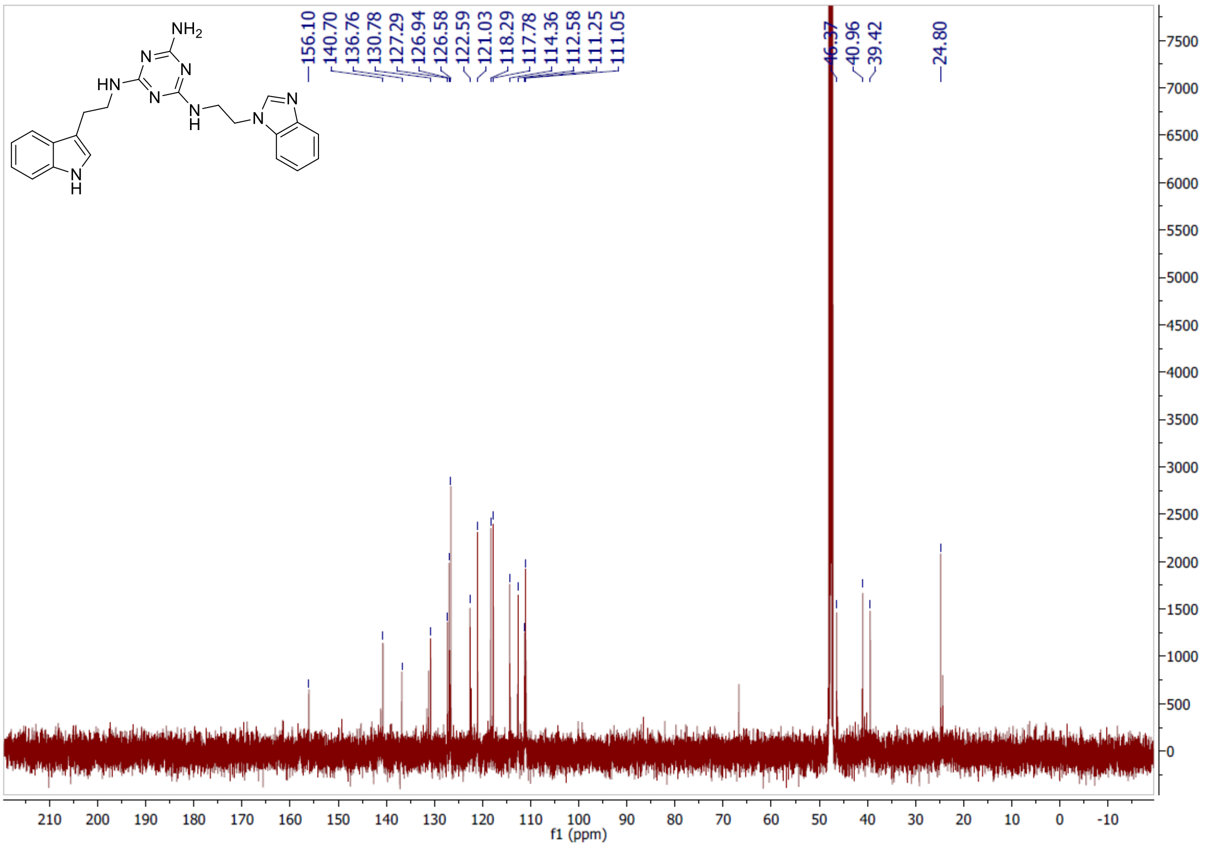

HPLC-MS of compound **18**

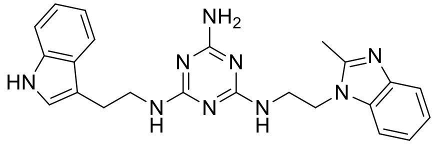

Chemical Formula: $C_{23}H_{25}N_9$
Exact Mass: 427,2

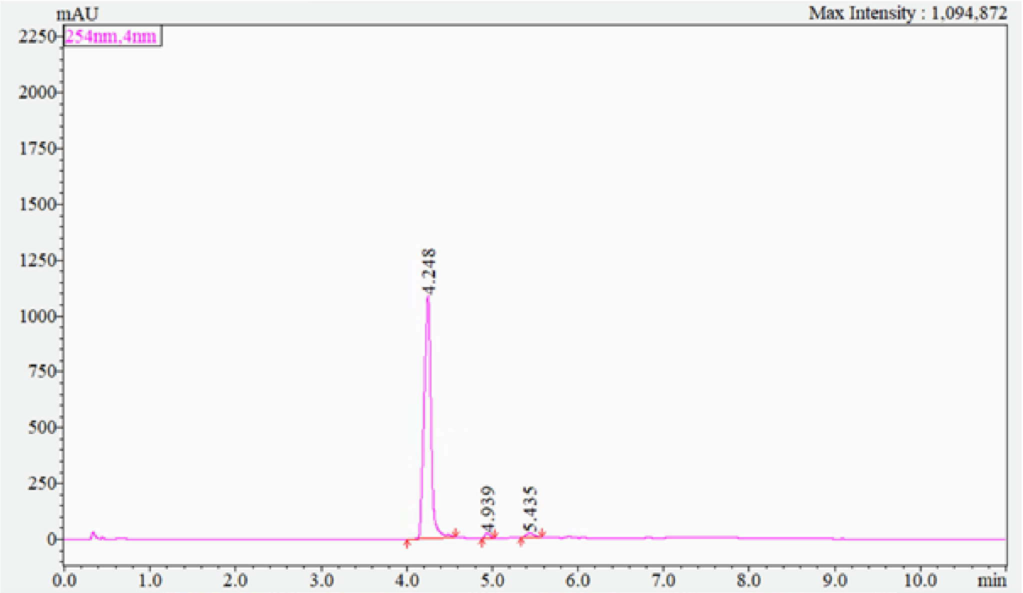

Peak#	Ret. Time	Area	Area%
1	4.248	6154809	96.631
2	4.939	90869	1.427
3	5.435	123691	1.942
Total		6369368	100.000

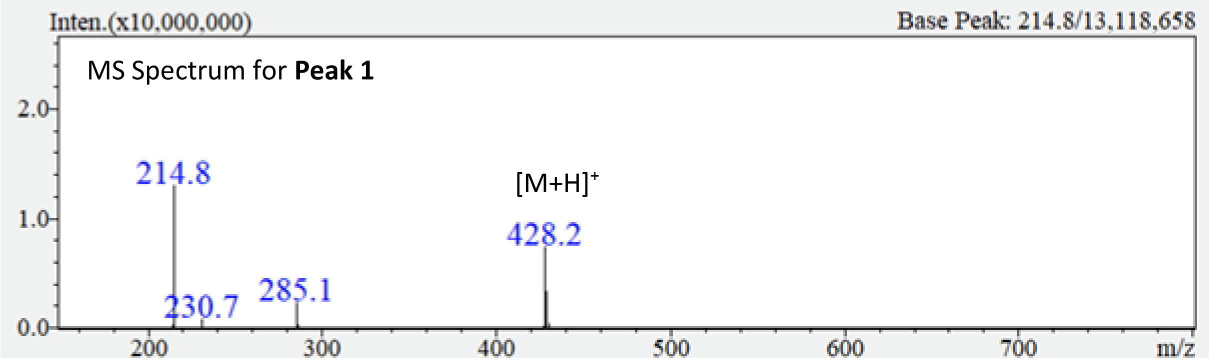

^{1}H NMR of compound **18** in CD_3OD

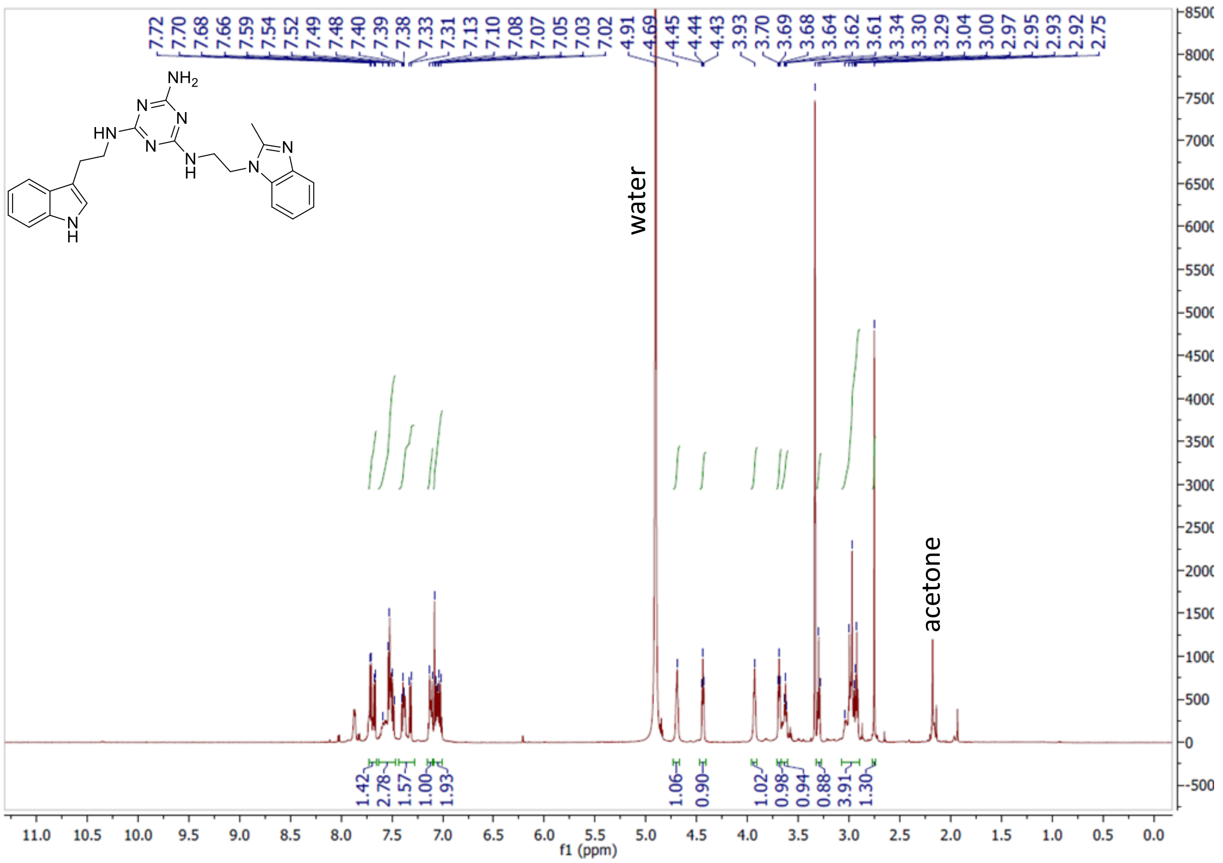

^{13}C NMR of compound **18** in CD_3OD

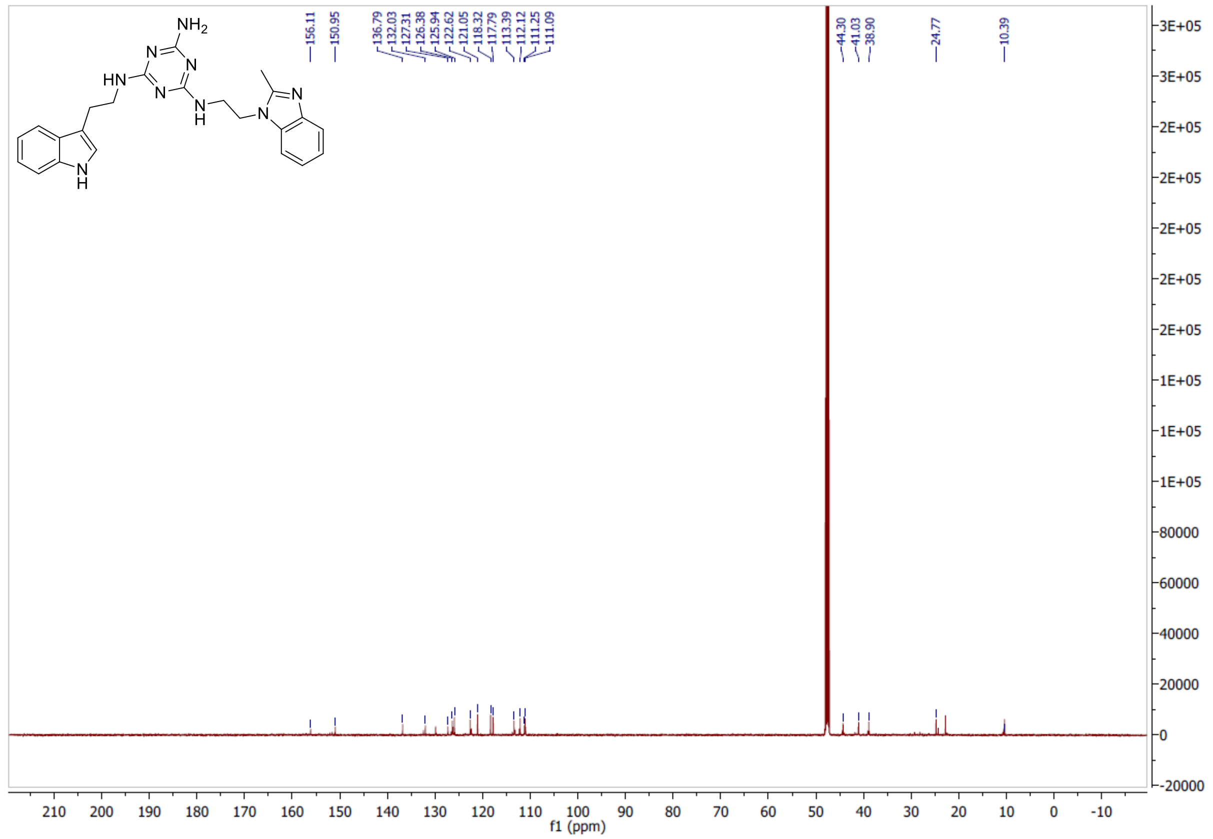

HPLC-MS of compound **19**

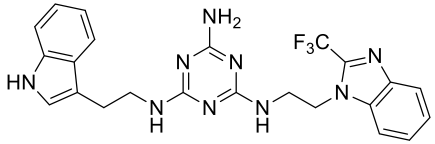

Chemical Formula: $C_{23}H_{22}F_3N_9$
Exact Mass: 481,2

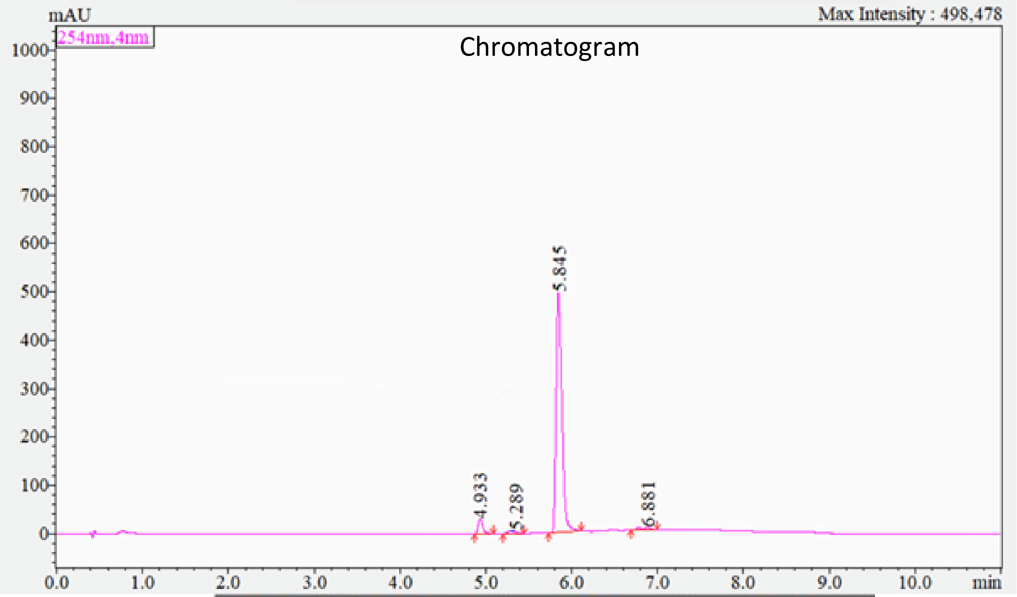

Peak#	Ret. Time	Area	Area%
1	4.933	119016	4.744
2	5.289	37219	1.484
3	5.845	2308676	92.034
4	6.881	43596	1.738
Total		2508507	100.000

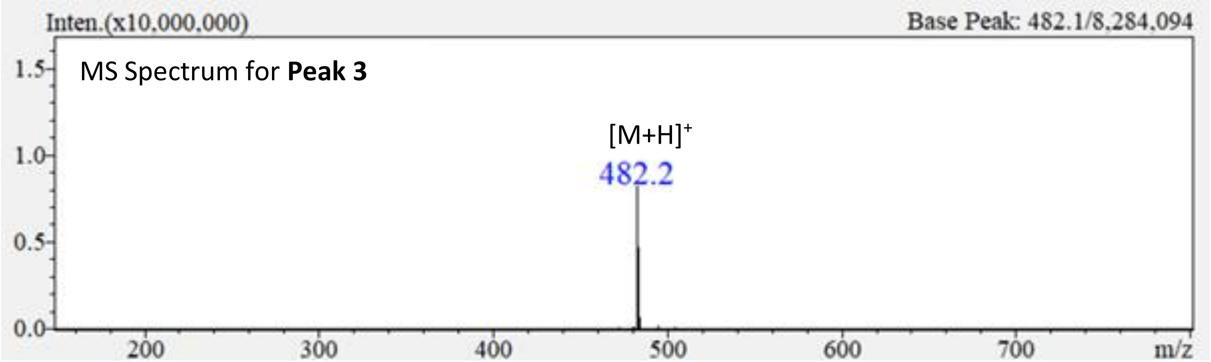

^{1}H NMR of compound **19** in CD_3OD

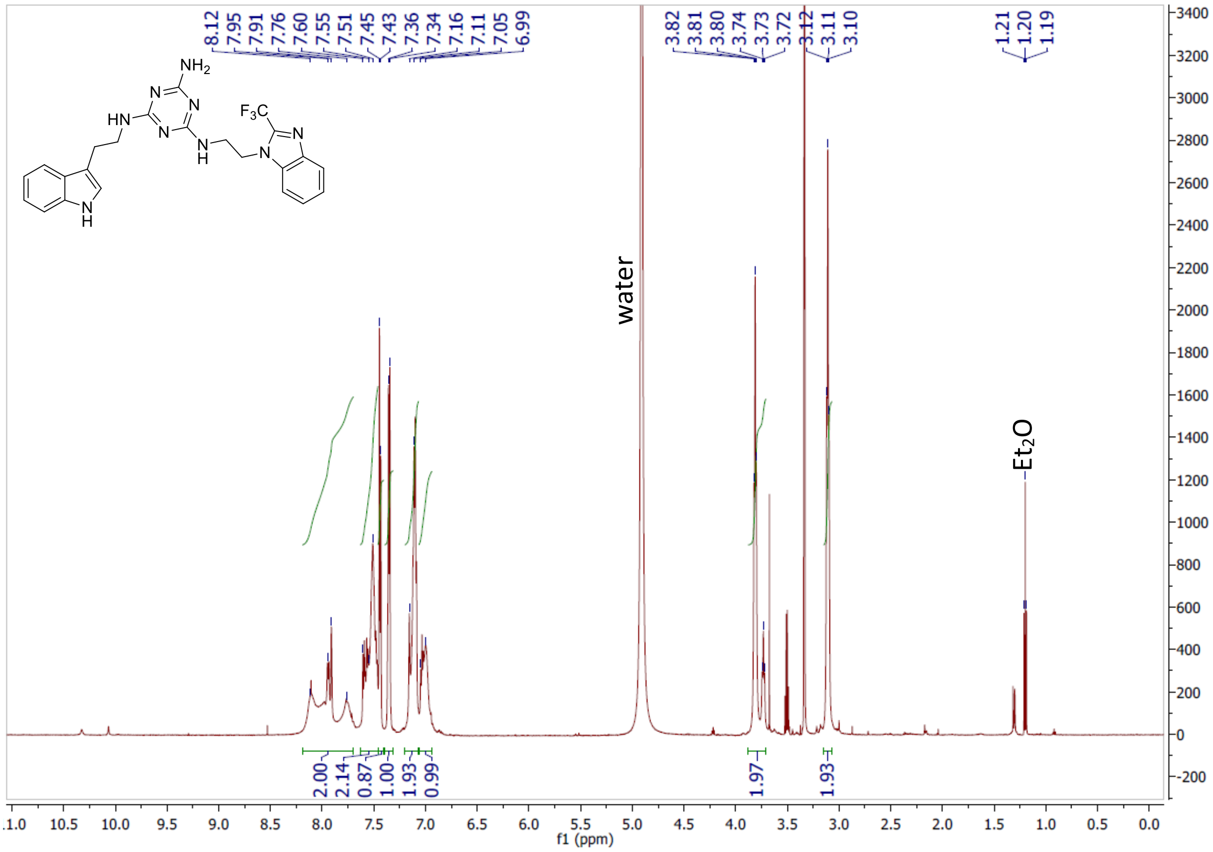

^{13}C NMR of compound **19** in CD_3OD

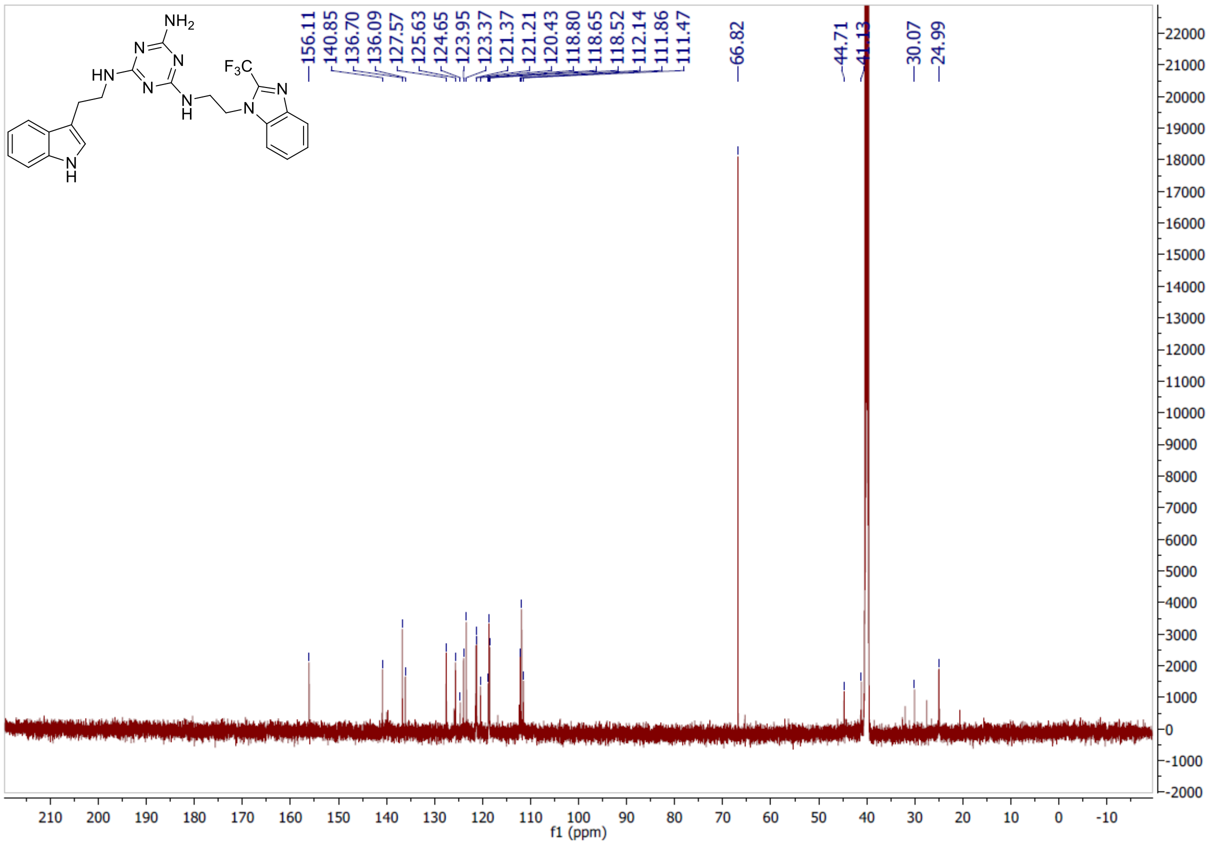

HPLC-MS of compound **20**

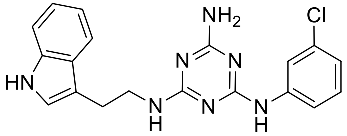

Chemical Formula: $C_{19}H_{18}{}^{35}ClN_7$
Exact Mass: 379,1

Chemical Formula: $C_{19}H_{18}{}^{37}ClN_7$
Exact Mass: 381,1

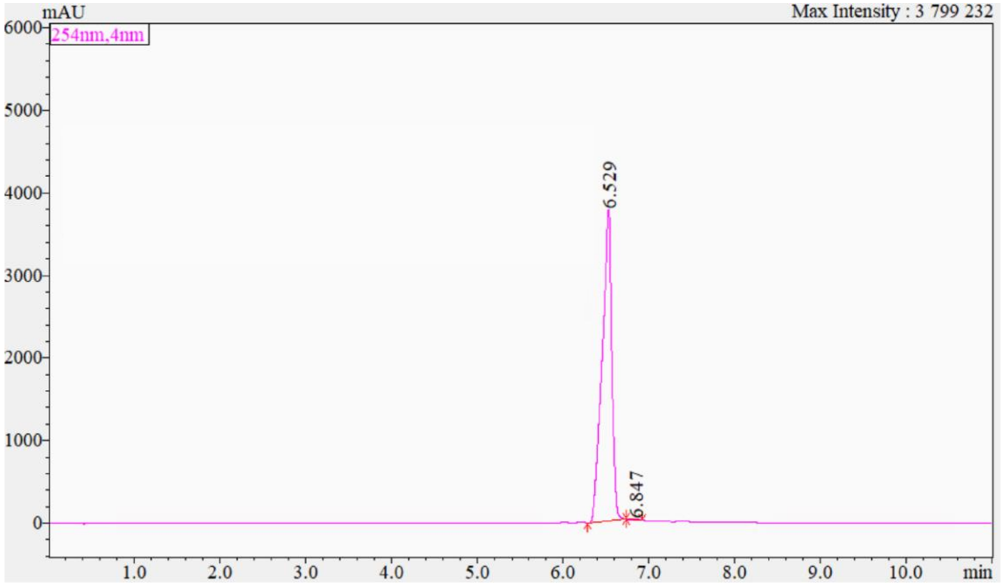

Peak#	Ret. Time	Area	Area%
1	6.529	29524309	99.956
2	6.847	12940	0.044
Total		29537250	100.000

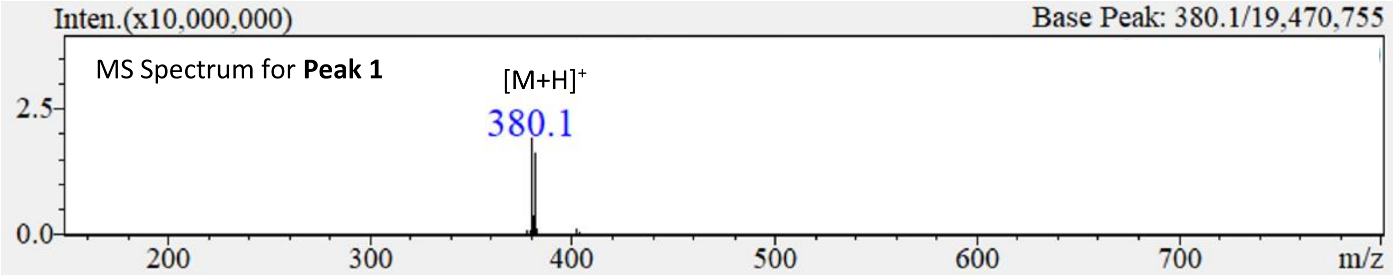

^{1}H NMR of compound **20** in CD_3OD

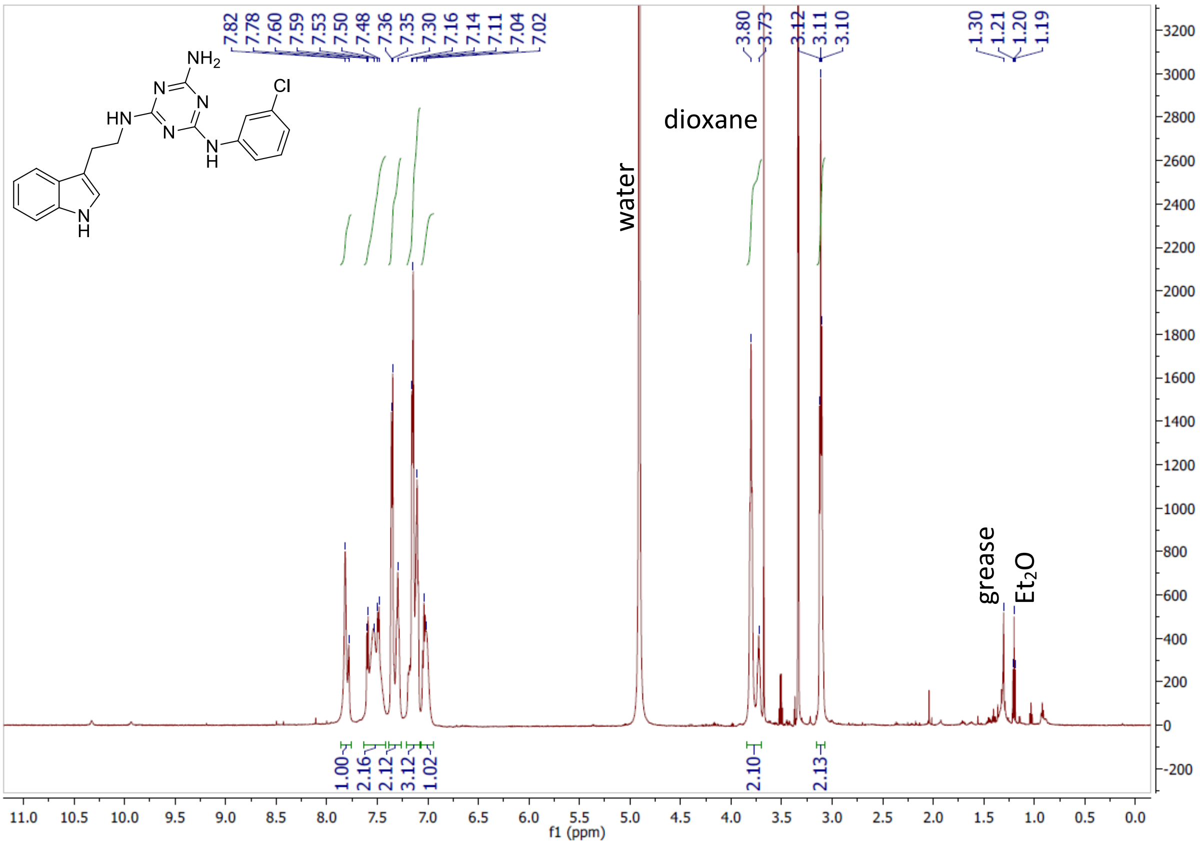

^{13}C NMR of compound **20** in CD_3OD

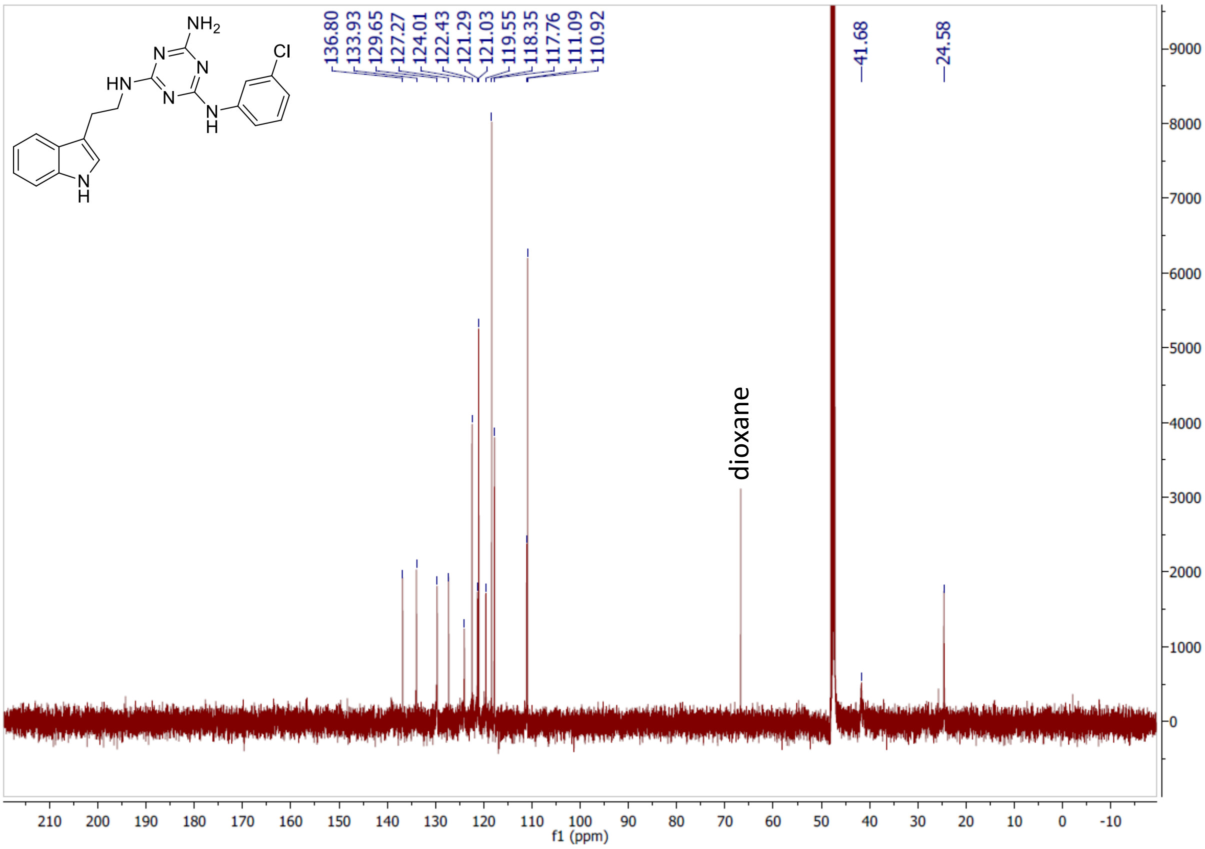

HPLC-MS of compound **21**

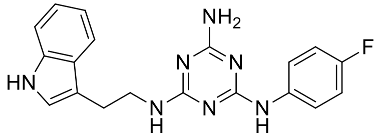

Chemical Formula: $C_{19}H_{18}FN_7$
Exact Mass: 363,2

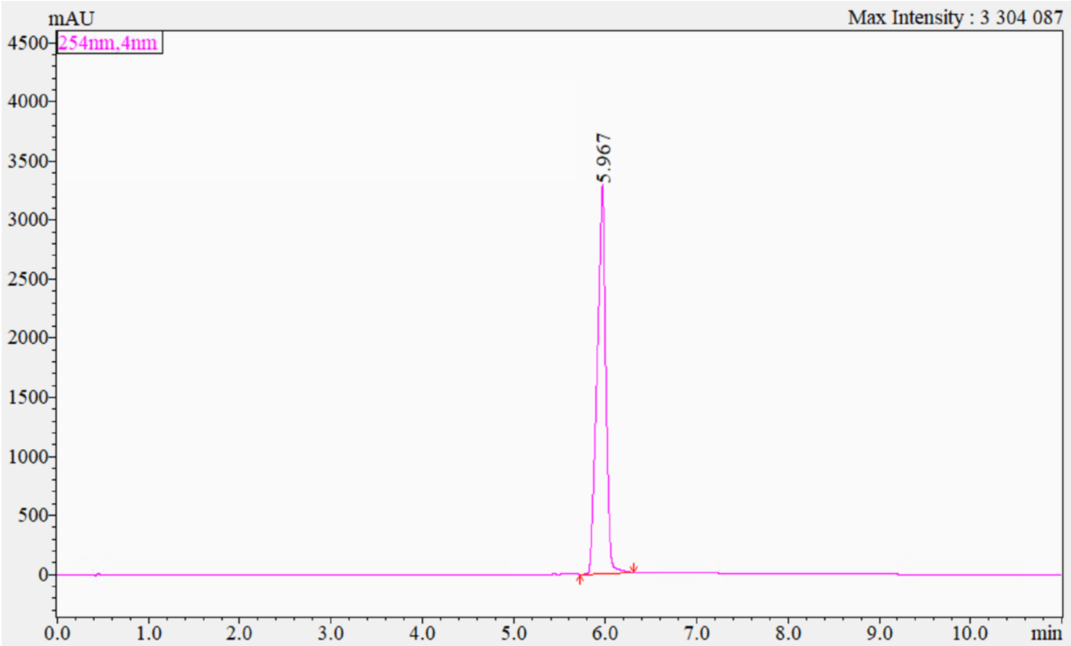

Peak#	Ret. Time	Area	Area%
1	5.967	21860967	100.000
Total		21860967	100.000

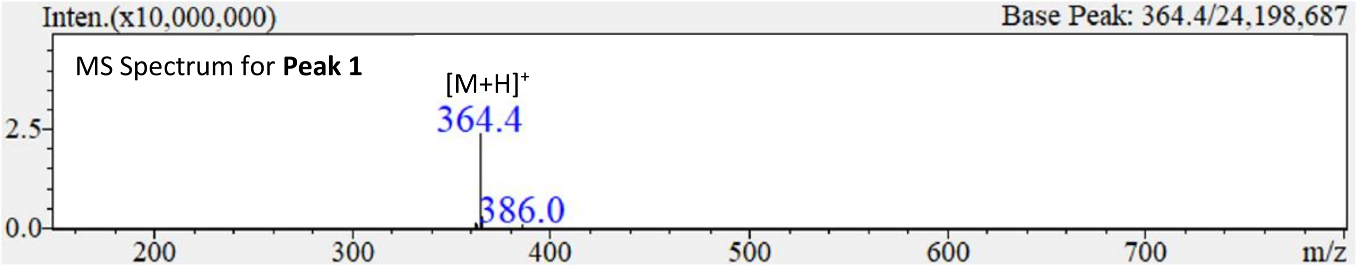

^1H NMR of compound **21** in CD_3OD

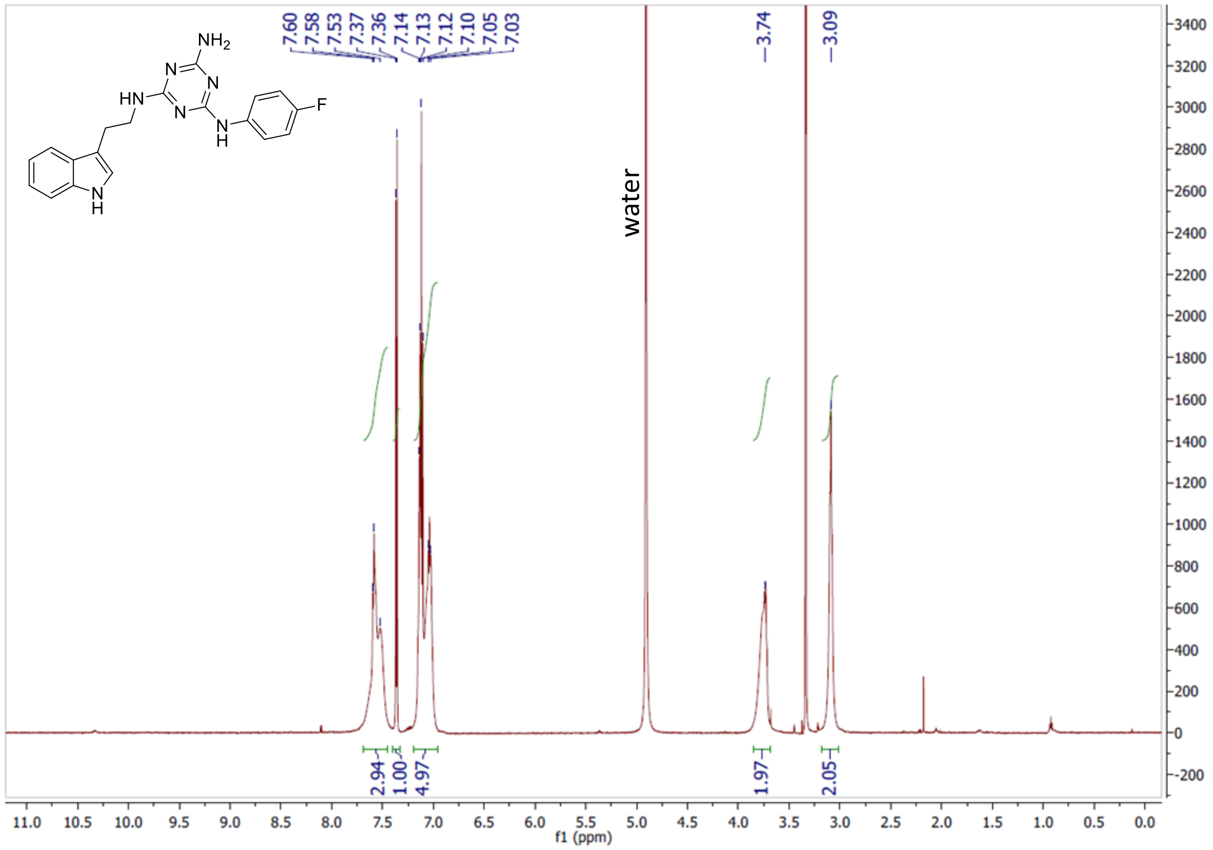

^{13}C NMR of compound **21** in CD_3OD

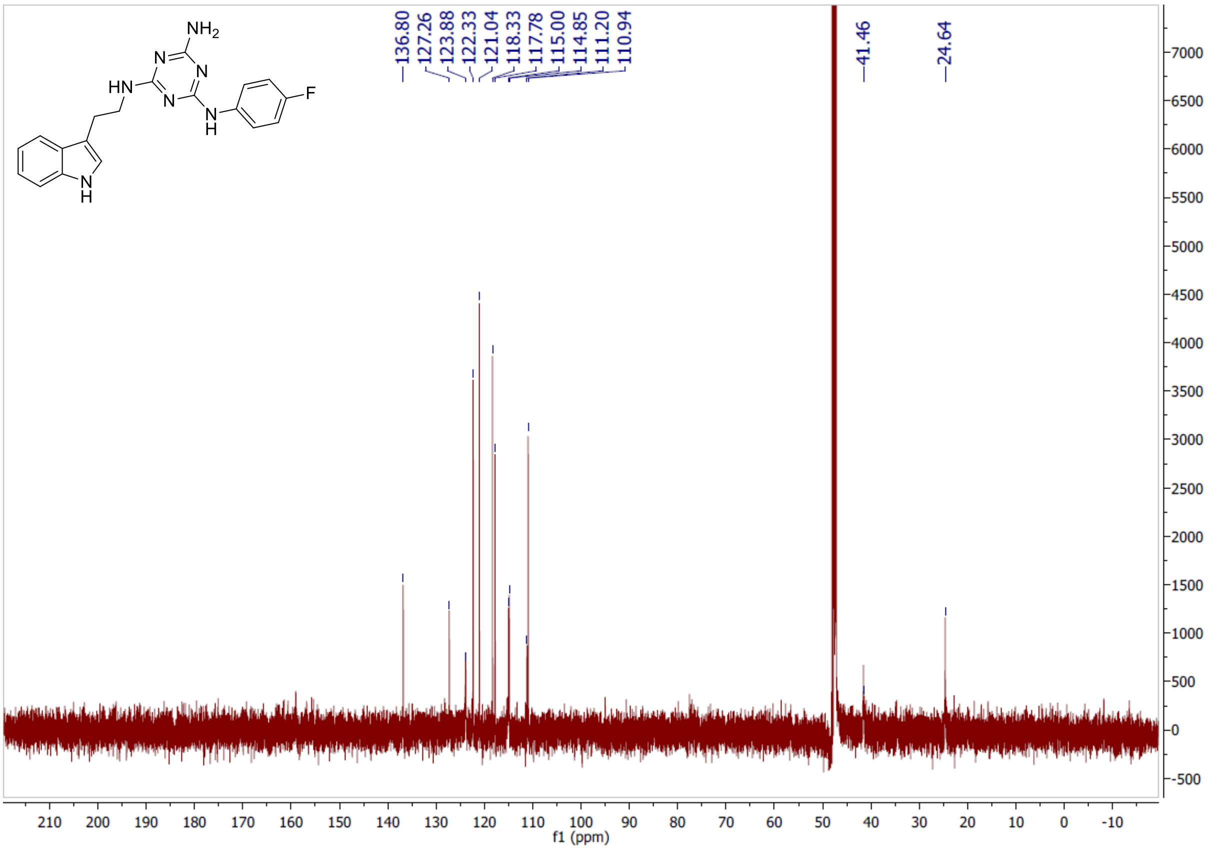

HPLC-MS of compound **22**

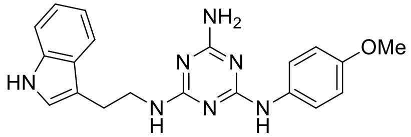

Chemical Formula: $C_{20}H_{21}N_7O$
Exact Mass: 375,2

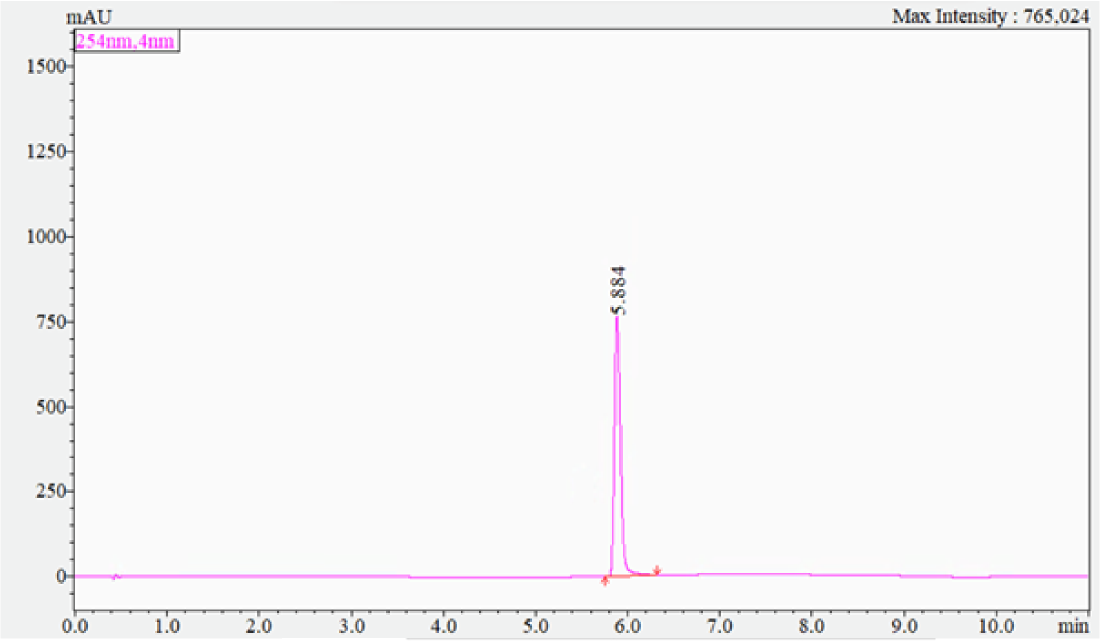

Peak#	Ret. Time	Area	Area%
1	5.884	3551723	100.000
Total		3551723	100.000

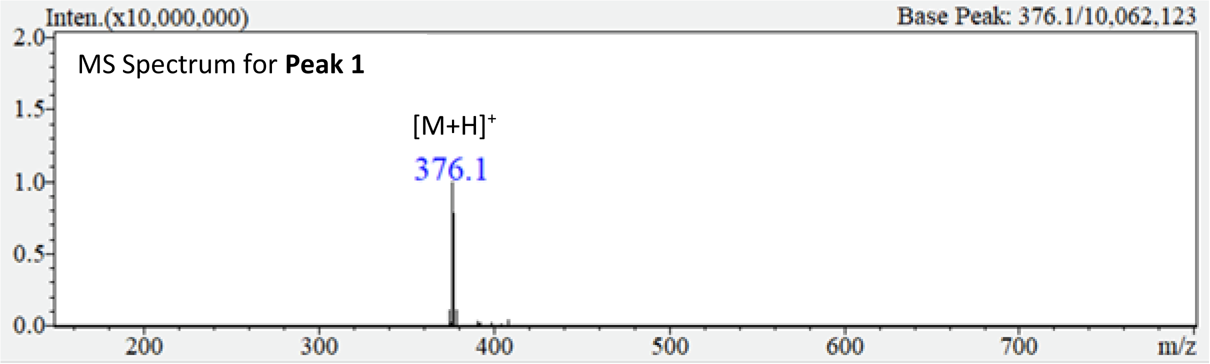

^{1}H NMR of compound **22** in CD_3OD

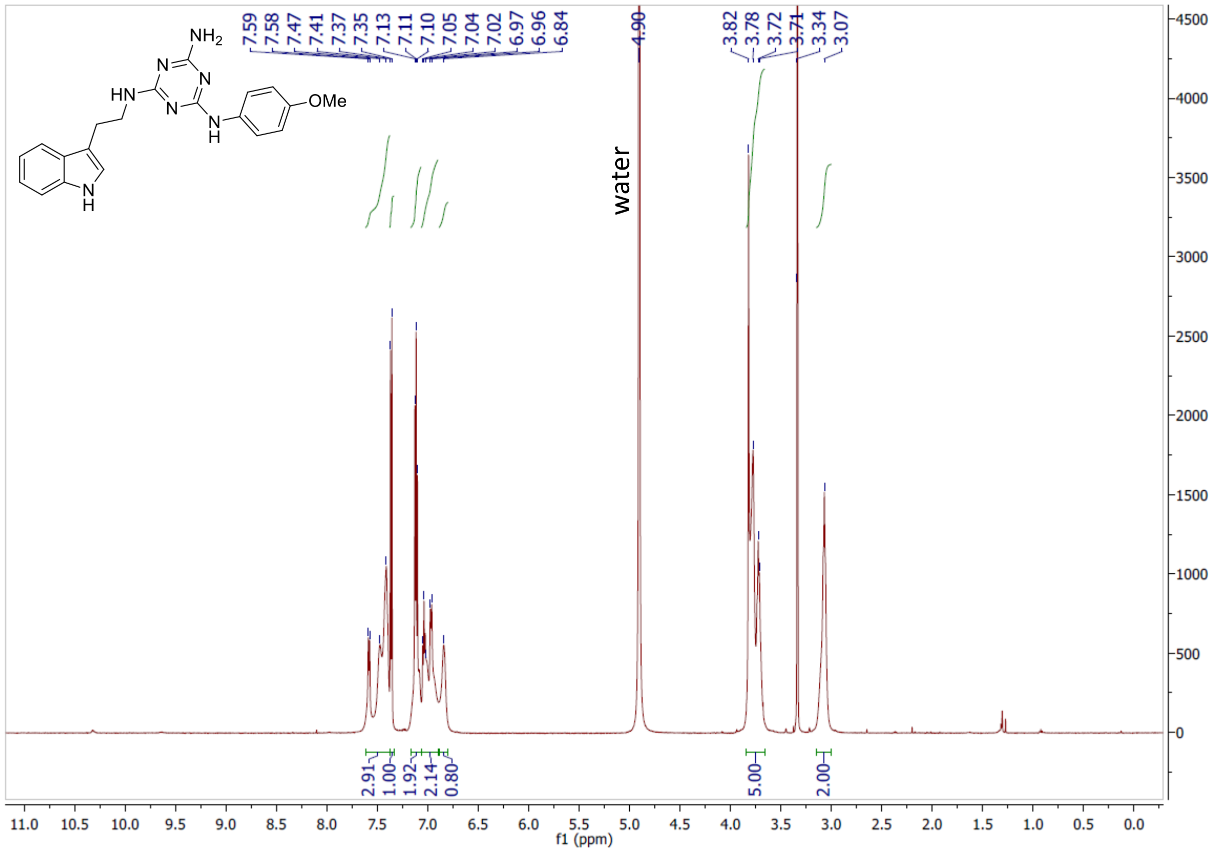

^{13}C NMR of compound **22** in CD_3OD

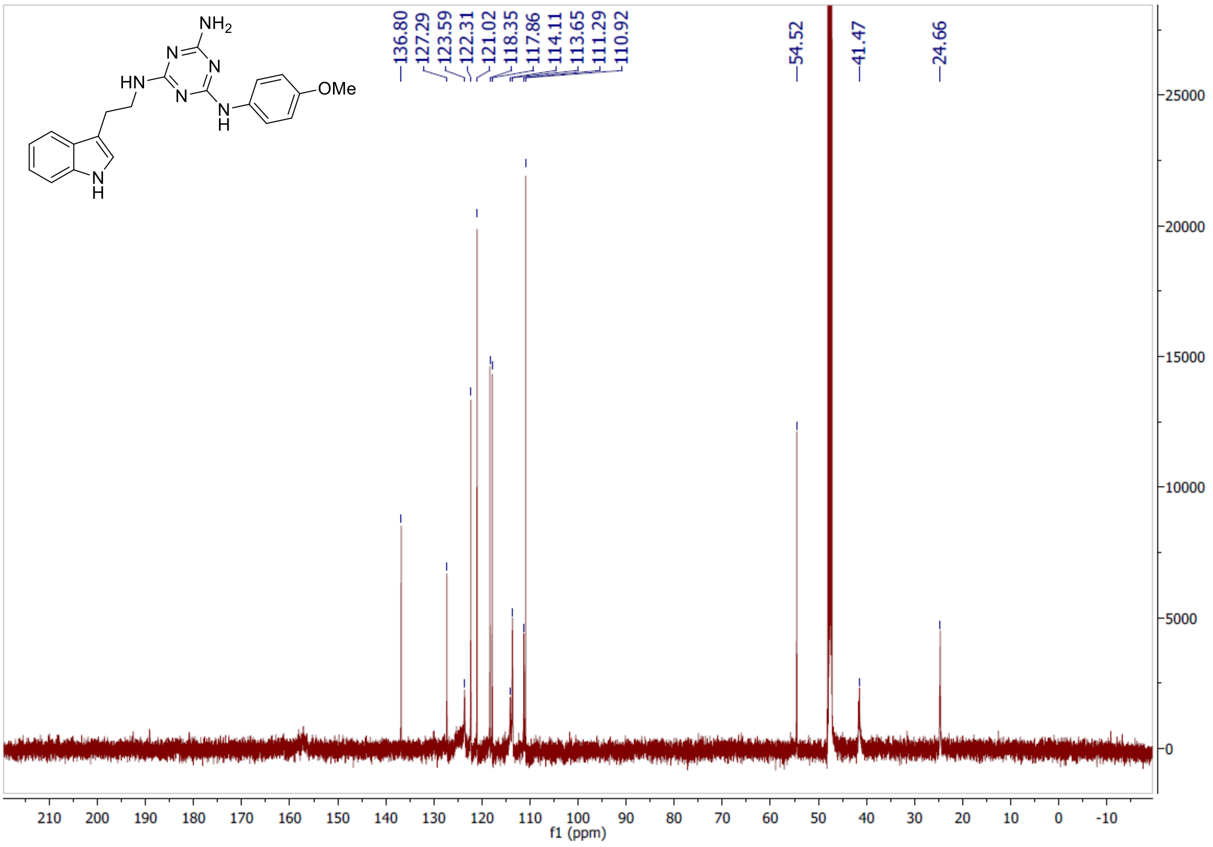

Metabolic stability UPLC-MS Spectra for ligands 2

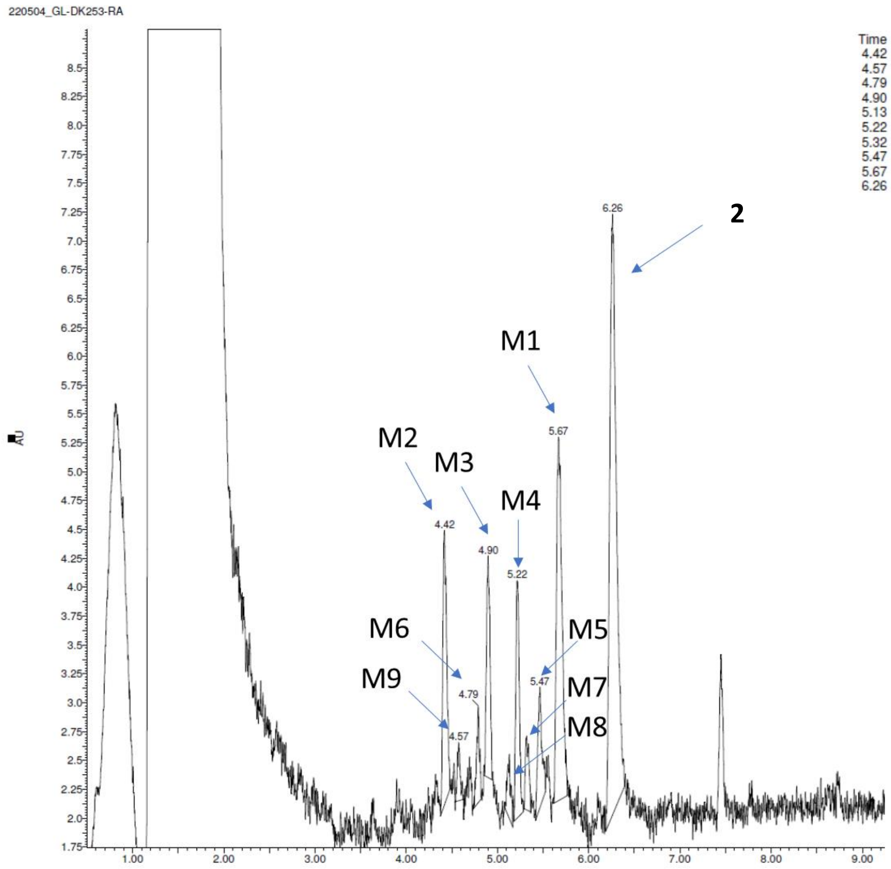

3: Diode Array
Range: 1.108e+2

Time	Height	Area	Area%
4.42	2383334	116826.45	10.54
4.57	506726	17597.71	1.59
4.79	835754	30871.60	2.79
4.90	1920419	85354.09	7.70
5.13	557778	23326.20	2.10
5.22	2057934	91219.90	8.23
5.32	640063	29019.42	2.62
5.47	1046900	54171.64	4.89
5.67	3147246	219372.56	19.79
6.26	5201590	440669.44	39.76

Figure UPLC after 120 min incubation of compound **2** with MLMs.

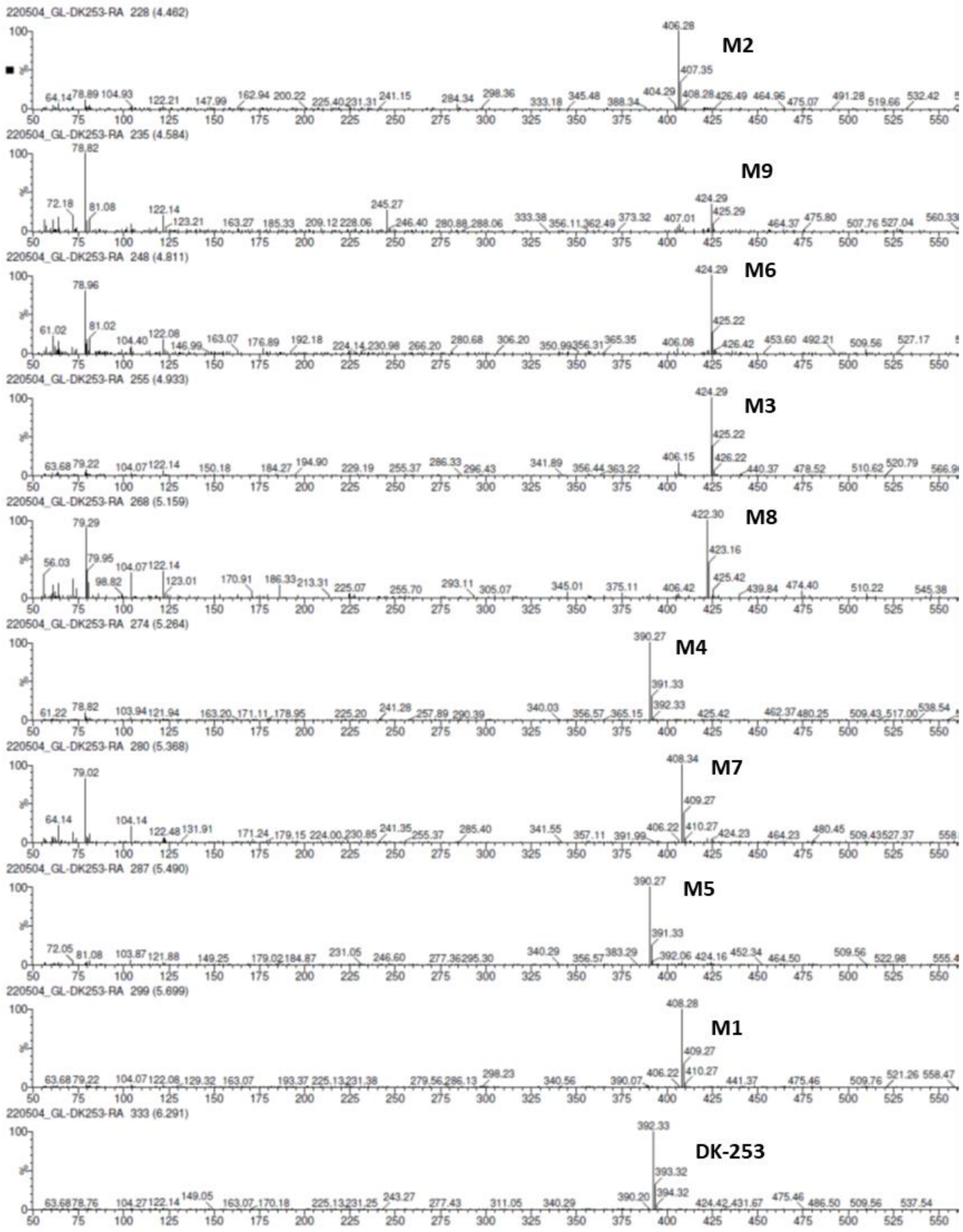

Figure MS analyses of ligand **2** and its metabolites **M1-M9**.

Metabolic stability UPLC-MS Spectra for ligand 12

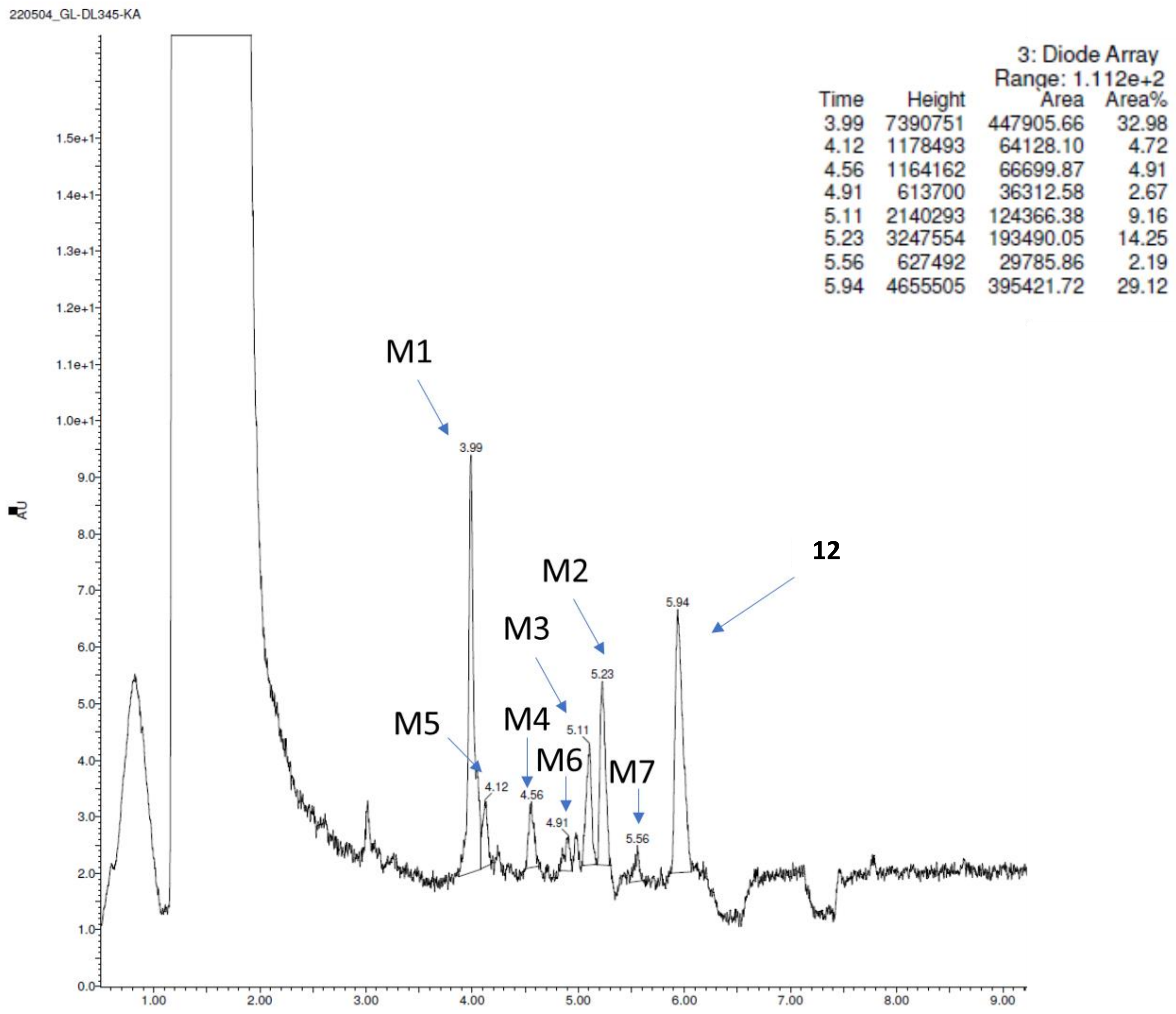

Figure UPLC after 120 min incubation of compound **12** with MLMs.

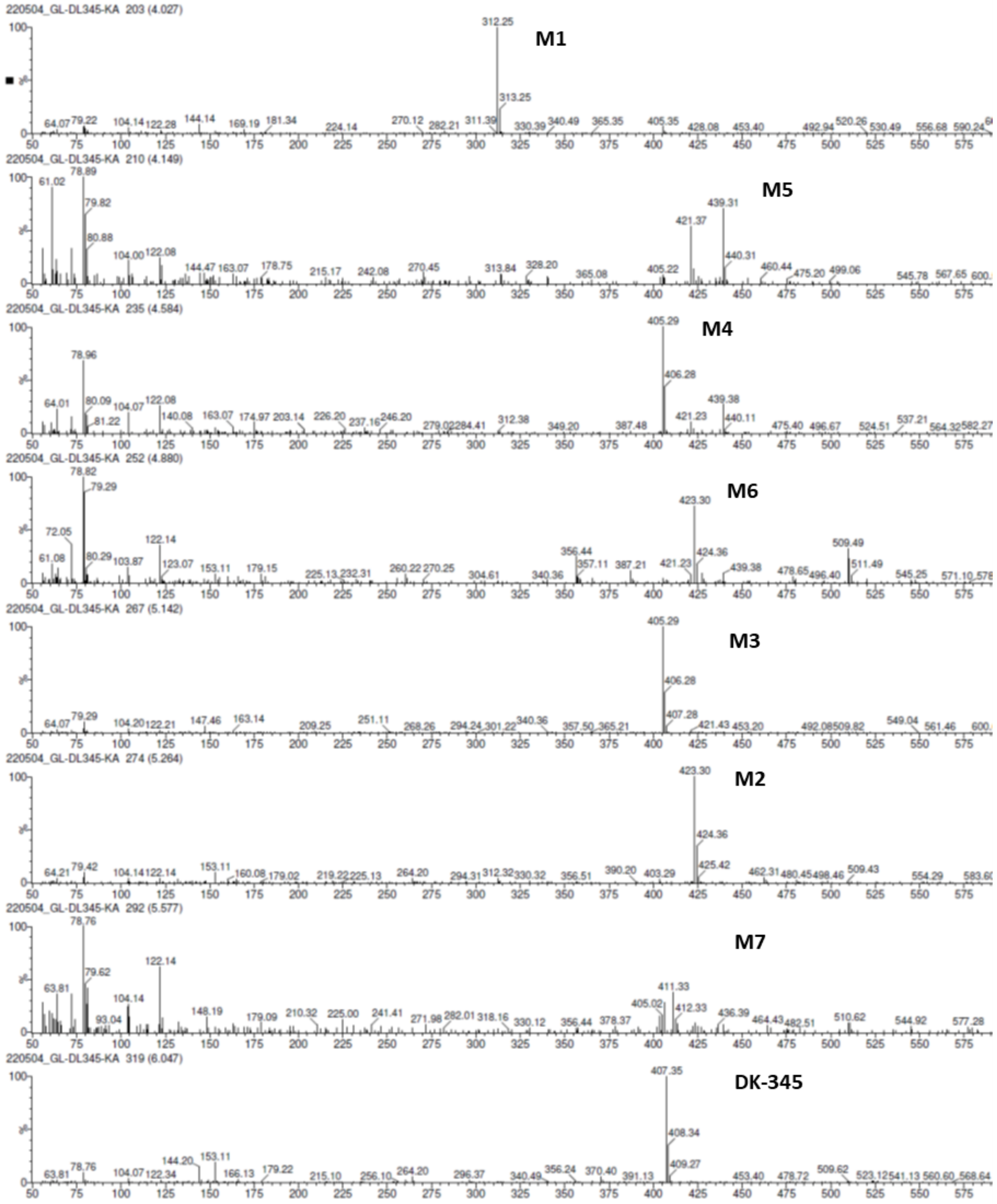

Figure MS analyses of ligand **12** and its metabolites M1-M7.

Ramachandran plot for 5-HT$_7$ homology model

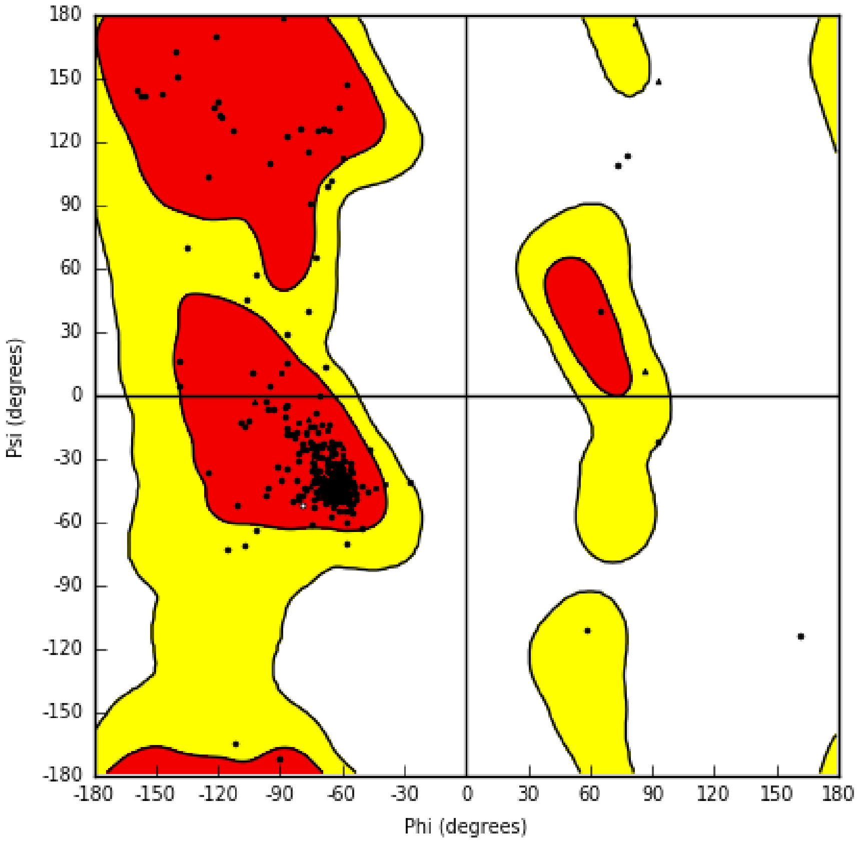

Validation of the5-HT$_7$ homology model

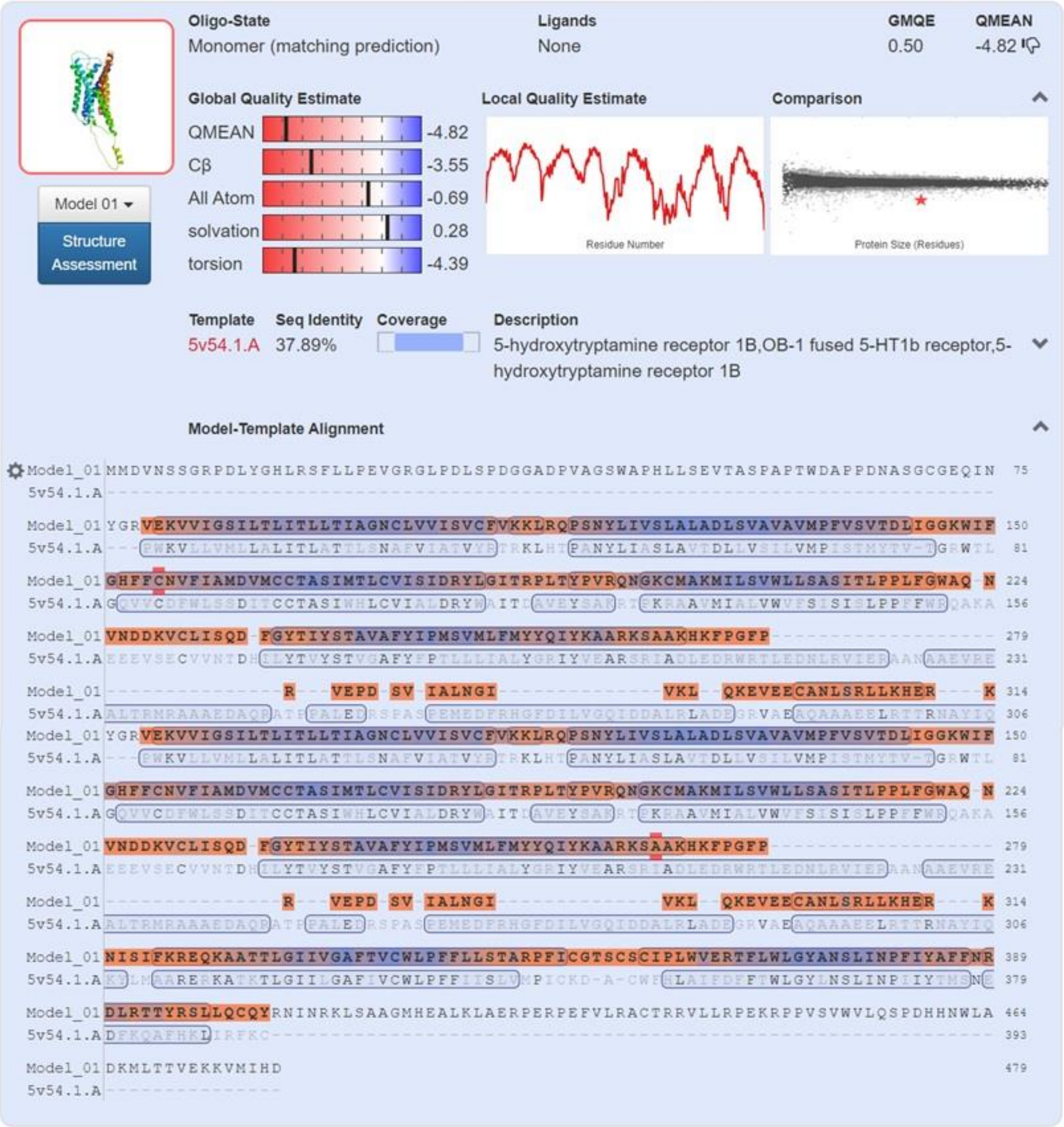

Supplement: Supplementary file 1 [file ijms-23-13308-s001.zip › ijms-1980475-SI.pdf]
